# Carbene catalyzed umpolung of α,β-enals: a reactivity study of diamino dienols *vs.* azolium enolates, and the characterization of advanced reaction intermediates†
†Electronic supplementary information (ESI) available: Experimental procedures, compound characterization data, and X-ray crystallographic data of compounds **4b-Et** and **4b-Me**. CCDC 1014843 and 1014844. For ESI and crystallographic data in CIF or other electronic format see DOI: 10.1039/c5sc01027f


**DOI:** 10.1039/c5sc01027f

**Published:** 2015-04-30

**Authors:** Veera Reddy Yatham, Jörg-M. Neudörfl, Nils E. Schlörer, Albrecht Berkessel

**Affiliations:** a Department of Chemistry , Cologne University , Greinstrasse 4 , 50939 Cologne , Germany . Email: berkessel@uni-koeln.de ; Fax: +49-221-470-5102 ; Tel: +49-221-470-3283

## Abstract

Since their discovery by Bode and Glorius in 2004, N-heterocyclic carbene catalyzed conjugate umpolung reactions of α,β-enals have been postulated to involve the formation of diamino dienols (“homoenolates”) and/or azolium enolates (“enolates”), typically followed by addition to electrophiles, *e.g.* Michael-acceptors. In this article, we provide evidence, for the first time, for the postulated individual and specific reactivity patterns of diamino dienols (γ-C–C-bond formation) *vs.* azolium enolates (β-C–C-bond formation). Our study is based on the pre-formation of well defined diamino dienols and azolium enolates, and the *in situ* NMR monitoring of their reactivities towards enone electrophiles. Additionally, reaction intermediates were isolated and characterized, *inter alia* by X-ray crystallography.

## Abstract 2

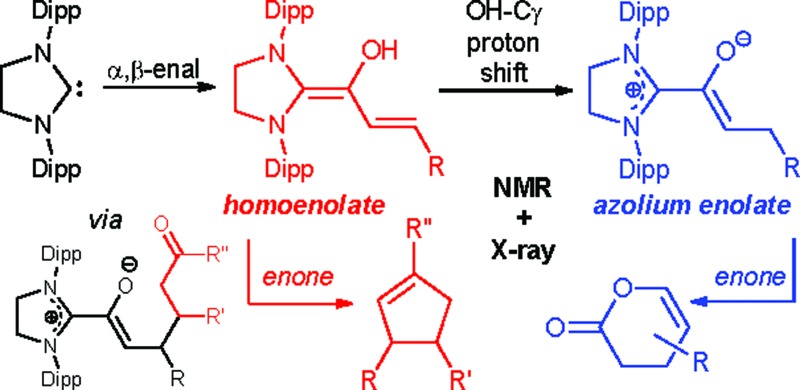
NMR/X-ray evidence is provided for hitherto postulated reactivity patterns of homoenolate *vs.* azolium enolate intermediates in NHC-catalyzed umpolung of enals.

## Introduction

**Scheme 1:**
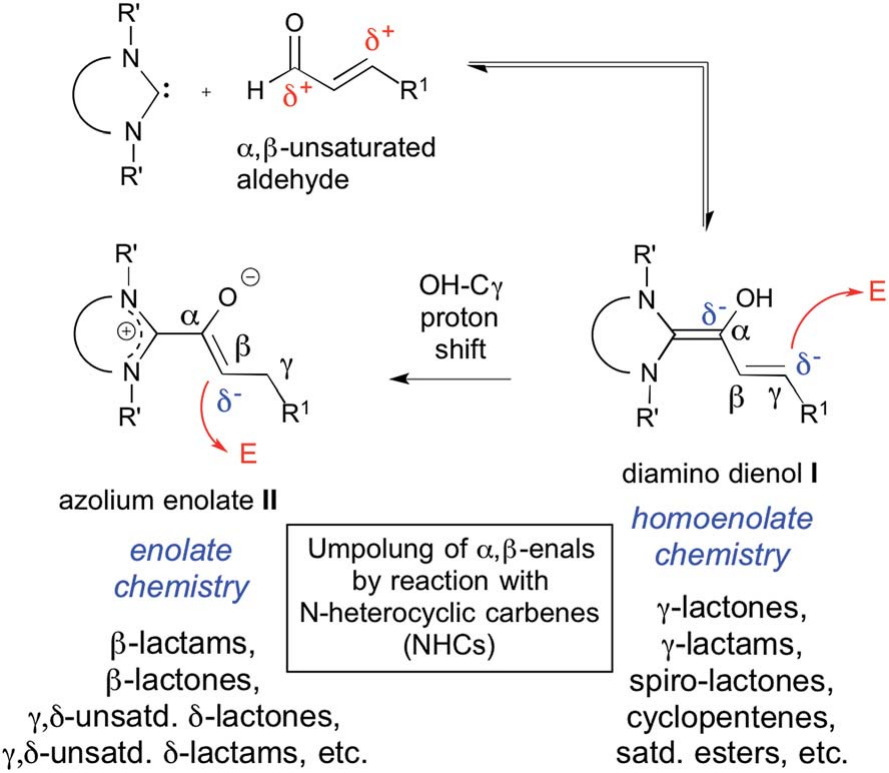
Early intermediates in the NHC-catalyzed umpolung of α,β-unsaturated aldehydes.

In N-heterocyclic carbene (NHC) organocatalysis,^1^ the “conjugate umpolung” of α,β-unsaturated aldehydes is a most thriving and proliferative field. As schematically shown in Scheme 1, interaction of an α,β-enal (a^3^) with an NHC first generates a Breslow-type^2^ intermediate, the diamino dienol **I**. A subsequent proton shift from the diamino dienol's –OH to Cγ leads to the azolium enolate **II**. The diamino dienol **I** carries a partial negative charge on Cγ, and therefore represents a homoenolate equivalent (d^3^). On the other hand, the azolium enolate **II** is nucleophilic at Cβ, and therefore behaves as an enolate equivalent (d^2^). Numerous experimental studies have revealed that the homoenolate *vs.* enolate behaviour of α,β-enals, when exposed to NHCs, can be influenced by the type of catalyst employed, and by the reaction conditions.^3^,^4^ For example, homoenolate chemistry is favoured by imidazolium precatalysts, in combination with strong bases.^3^,^4^ Reactions proceeding *via* the homoenolate pathway have been used to provide γ-lactones,^5^ spiro-lactones,^6^ spiro-bis-lactones,^7^ bicyclic lactones,^8^ γ-lactams,^9^ bicyclic β-lactams,^10^ cyclopentenes,5c,^11^ and saturated esters.^12^ Enolate chemistry, on the other hand, is favoured by triazolium precatalysts in combination with weak bases.^3^,^4^ Azolium enolates have been generated by the combination of NHCs with ketenes,^13^ aldehydes,3a,^14^ and esters.^15^ Reactions proceeding *via* the azolium enolate pathway have been used to provide β-lactams,13b,c β-lactones,13d,e unsaturated δ-lactams,14b,f,15b,c and unsaturated δ-lactones.14a,14e–g,15c

As outlined in Scheme 1, it is generally believed that diamino dienols **I** and the tautomeric azolium enolates **II** are the starting points of divergent reaction pathways, leading to different (isomeric) products when exposed to one and the same electrophilic reaction partner. This divergent reactivity is interpreted in the sense that diamino dienols **I** add electrophiles at Cγ, whereas the tautomeric azolium enolates **II** react at Cβ. In stark contrast to their pivotal importance in α,β-enal umpolung, no investigations of the reaction modes of pre-formed diamino dienols **I** and azolium enolates **II** (*i.e*. C–C bond formation with C-electrophiles at Cβ *vs.* Cγ) appear to have been reported to date.^16^ Several azolium enolates **II** are described in the literature. However, they were accessed by addition of carbenes to ketenes,^16^,^17^ and not by reaction of α,β-unsaturated aldehydes with N-heterocyclic carbenes (NHCs). With this in mind, we set out to investigate the reactivity patterns of pre-formed diamino dienols **I** and azolium enolates **II** with enone Michael acceptors. The first successful generation of both diamino dienols **I** and azolium enolates **II** from α,β-unsaturated aldehydes and carbenes, and their characterization by NMR and X-ray, was recently reported by our group.^18^

## Results and discussion

### Reactivity studies of diamino dienols

#### Cyclopentene formation with enones

**Scheme 2:**
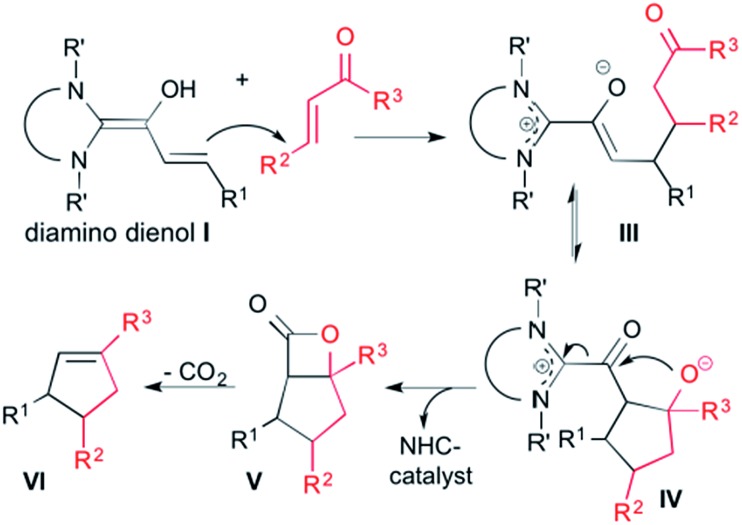
Proposed mechanism for cyclopentene (**VI**) formation from diamino dienol **I** and an enone Michael acceptor.

In 2006, Nair *et al.* reported that the NHC-catalyzed reaction of cinnamic aldehydes with enones affords 1,3,4-trisubstituted cyclopentenes.11*a* As schematically shown in Scheme 2, this transformation was interpreted by homoenolate addition to the Michael acceptor, giving rise to the intermediate **III**.^19^ Aldol ring closure leads to intermediate **IV**. From there, the β-lactone **V** is formed, with concomitant regeneration of the NHC catalyst. Decarboxylation of the β-lactone **V** finally gives the cyclopentene product **VI**.

**Scheme 3:**
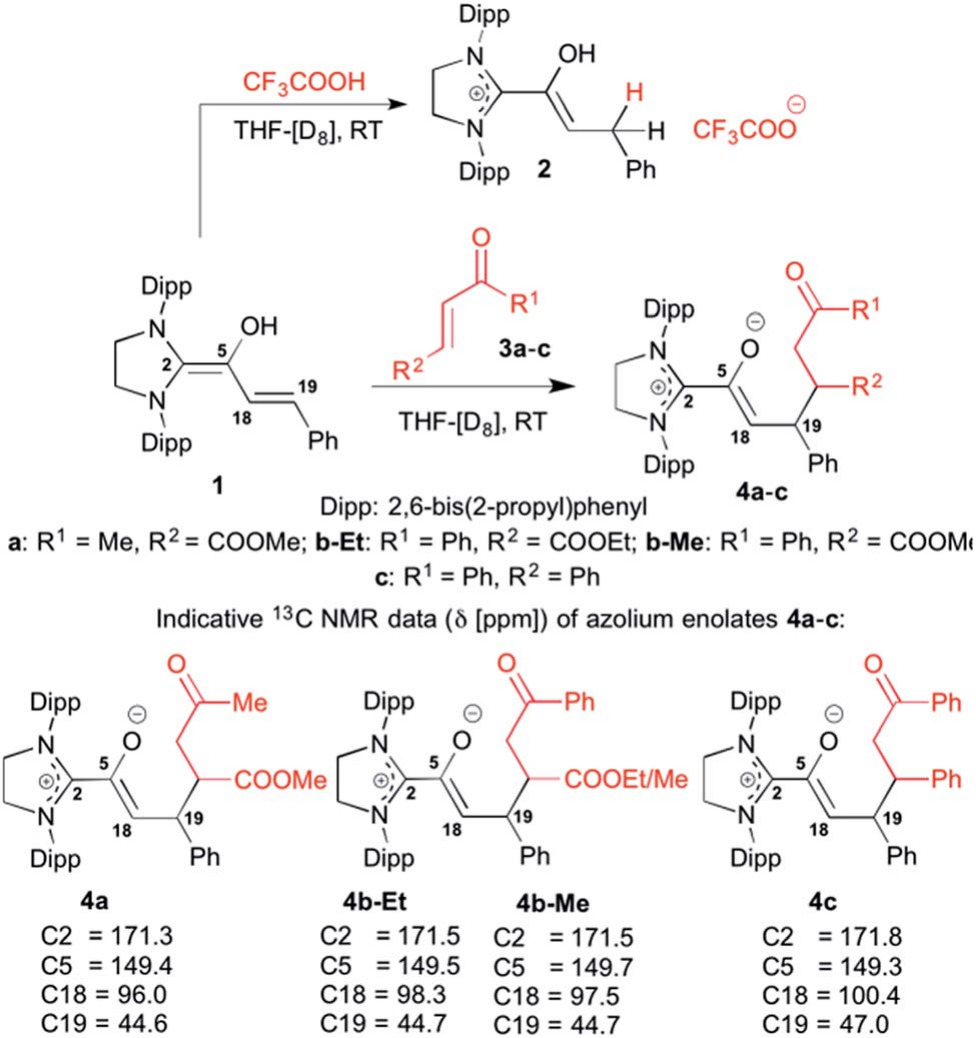
Top: diamino dienol **1** reacts with TFA to the azolium enol **2**, and (middle) with the enone electrophiles **3a–c** to afford the Michael addition adducts **4a–c**; bottom: characteristic ^13^C NMR shifts [ppm] of C2, C5, C18 and C19 of the Michael addition products **4a–c** ([D_8_]THF, 25 °C); Dipp = 2,6-bis(2-propyl)phenyl.

**Fig. 1:**
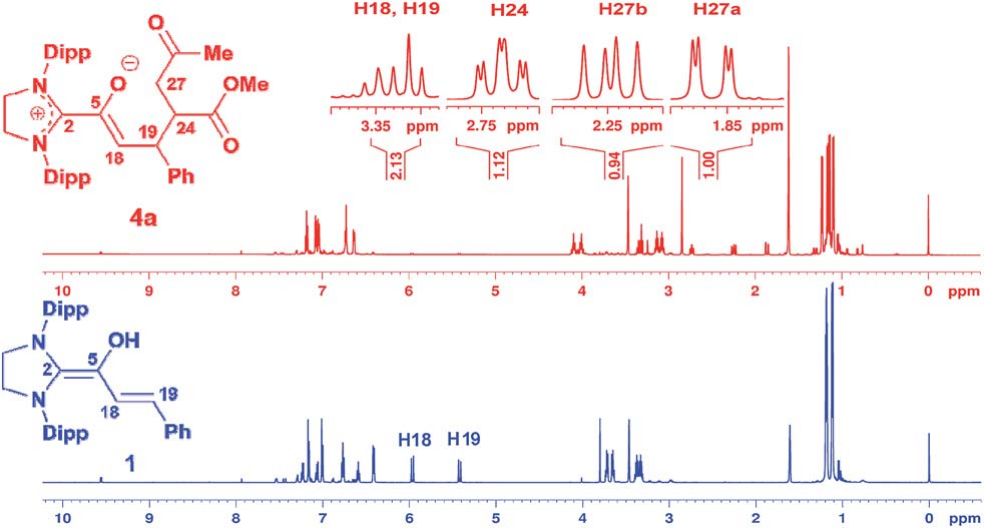
Top: ^1^H NMR spectrum ([D_8_]THF, 600 MHz) obtained upon addition of methyl *E*-4-oxo-2-pentenoate (**3a**) to the diamino dienol **1**, indicating the formation of Michael addition product **4a**; bottom: ^1^H NMR of the starting diamino dienol **1**; Dipp = 2,6-bis(2-propyl)phenyl.

We had reported earlier^18^ that under strictly oxygen-free conditions, the saturated imidazolidinylidene SIPr (1,3-bis[2,6-di-(2-propylphenyl)]imidazolidin-2-ylidene) reacts smoothly with *E*-cinnamic aldehyde in THF at room temperature to the diamino dienol **1** (Scheme 3). Protonation of the latter exclusively gives the Cγ-protonation product **2** (an azolium enol), and thus nicely proves Cγ-nucleophilicity (Scheme 3, top). When the pre-formed and stable diamino dienol **1** was exposed to an equimolar amount of methyl-*E*-4-oxo-2-pentenoate **3a** (Scheme 3, middle) under ^1^H NMR monitoring at room temperature, we observed the instantaneous disappearance of the signals characteristic of the diamino dienol **1** (Fig. 1, bottom: doublets at *δ* = 5.96 ppm, ^3^*J*_*HH*_ = 15.2 Hz, 1H, H18, and *δ* = 5.42 ppm, ^3^*J*_*HH*_ = 15.2 Hz, 1H, H19), with concomitant formation of a new species (Fig. 1, top). The newly formed sets of signals are consistent with the formation of the Michael addition product, the azolium enolate **4a** that results from C–C bond formation at Cγ of the diamino dienol **1**. For example, characteristic ^1^H NMR signals of **4a** are a multiplet at *δ* = 3.36–3.30 ppm (2H, H18, H19), a triplet of doublets at *δ* = 2.73 ppm (^3^*J*_*H*24–*H*27*a*_ = 2.9 Hz, ^3^*J*_*H*24–*H*27*b*_ = 11.4 Hz, ^3^*J*_*H*24–*H*19_ = 11.4 Hz, 1H, H24), a doublet of doublets at *δ* = 2.25 ppm (^3^*J*_*H*27*b*–*H*24_ = 11.4 Hz, ^2^*J*_*H*27*b*–*H*27*a*_ = 17.4 Hz, 1H, H27b), and a doublet of doublets at *δ* 1.86 ppm (^2^*J*_*H*27*a*–*H*27*b*_ = 17.4 Hz, ^3^*J*_*H*27*a*–*H*24_ = 2.9 Hz, 1H, H27a). Similarly indicative, in the ^13^C NMR spectrum, the signals of C2, C5, C18 and C19 shifted from 145.0 to 171.3 ppm, 114.0 to 149.4 ppm, 125.3 to 96.0 ppm, and 110.0 to 44.6 ppm, respectively (see ESI† for 1D and 2D NMR characterization of **4a**).

**Fig. 2:**
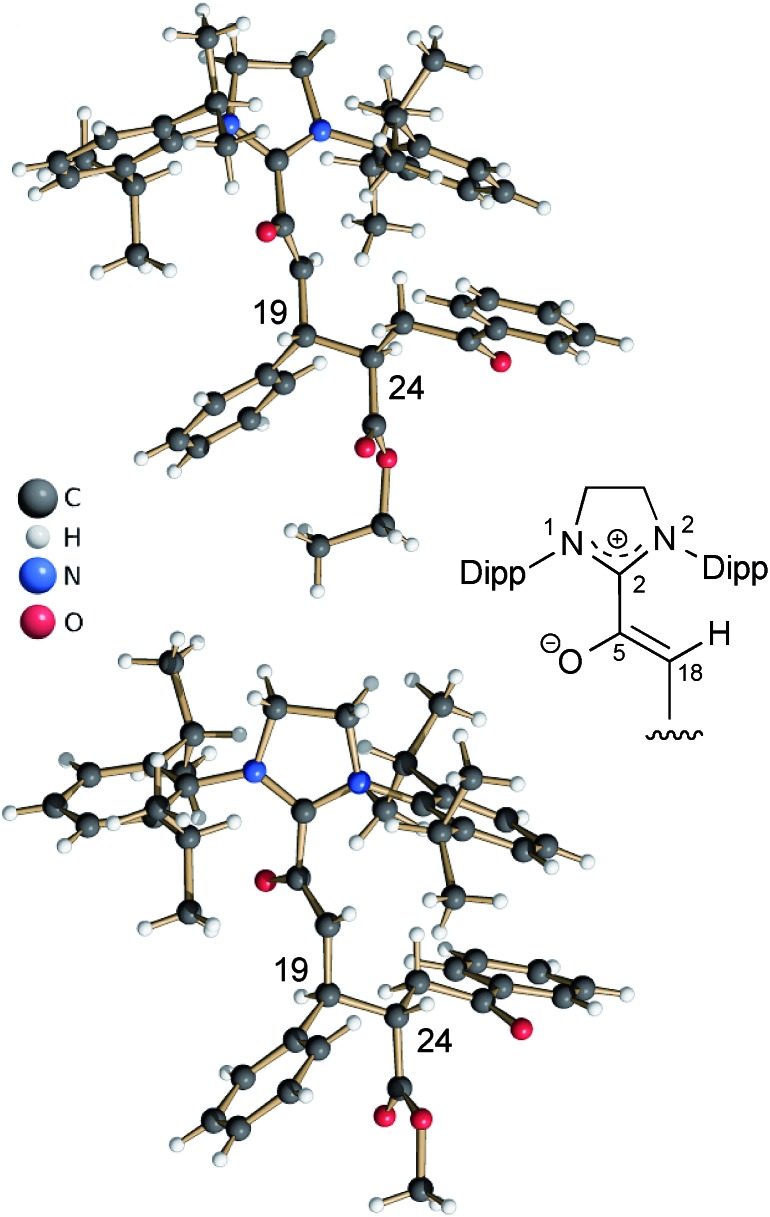
Top: X-ray crystal structure of the Michael product **4b-Et**, obtained from the addition of ethyl *E*-3-benzoylacrylate (**3b-Et**) to the diamino dienol **1**; bottom: X-ray crystal structure of the Michael product **4b-Me** obtained from diamino dienol **1** and methyl *E*-3-benzoylacrylate (**3b-Me**).

In the same manner, we exposed the diamino dienol **1** to an equimolar amount of ethyl *E*-3-benzoylacrylate (**3b-Et**). Again, NMR monitoring revealed the instantaneous disappearance of diamino dienol **1**, with concomitant formation of the corresponding Michael product **4b-Et** (Scheme 3, middle; see ESI† for the full 1D and 2D NMR characterization of **4b-Et**). In addition, crystallization of this Michael product **4b-Et** and of its methyl analogue, **4b-Me** [obtained from methyl 3-benzoylacrylate (**3b-Me**)], was achieved from benzene and THF solution, respectively, by slow addition of *n*-hexane at room temperature, and under strictly anaerobic conditions. The X-ray crystal structures of the azolium enolates **4b-Et** and **4b-Me** are shown in Fig. 2. First of all, the X-ray structures provide unambiguous proof for the formation and the constitution of the Michael addition products **4b-Et/Me**. Furthermore, they nicely reveal the almost orthogonal arrangement of the imidazolium ring and the enolate moiety, as evidenced by the dihedral angles O–C5–C2–N1 = 44.5(4)° and O–C5–C2–N2 = –132.3(3)° for **4b-Et**, and [O–C5–C2–N1 = –128.7(4)° and O–C5–C2–N2 = 47.8(5)° for **4b-Me**. Along the 5-oxy-4-pentenoate chain of the Michael products **4b-Et/Me**, the substituents at C19 (phenyl) and at C24 (phenacetyl) occupy *anti*-positions.

**Scheme 4:**
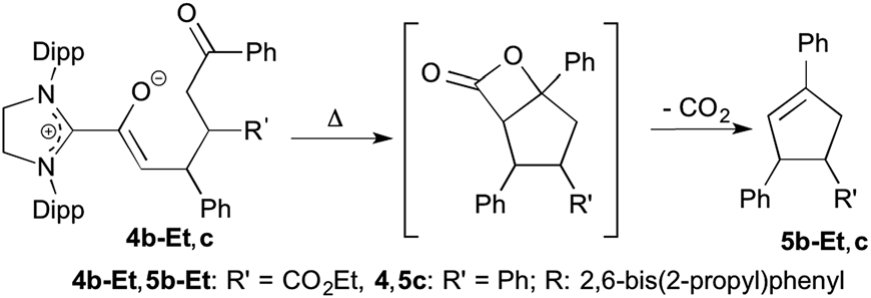
Heating-induced conversion of the Michael addition products **4b-Et** and **4c** to the cyclopentenes **5b-Et** and **5c**, respectively; Dipp = 2,6-bis(2-propyl)phenyl.

When the diamino dienol **1** was exposed to *E*-chalcone (**3c**) in an analogous manner, the slow formation of the Michael addition product **4c** was observed (Scheme 3, middle; *ca.* 80% conversion at room temperature after *ca.* 12 h; see ESI† for full NMR characterization of **4c**). In summary, in all four cases studied (diamino dienol **1** + enones **3a**, **3b-Et/Me**, **3c**), C–C bond formation had indeed occured at C-γ, of the diamino dienol and gave the azolium enolate intermediates **4a**, **4b-Et/Me** and **4c** postulated for cyclopentene formation.^11^ The further conversion of the azolium enolate intermediates such as **4a**, **4b-Et/Me**, and **4c** is typically formulated as an aldol addition of the enolate to the ketone moiety, followed by β-lactone formation and decarboxylation (*vide supra*, Scheme 2). Note that intermediate azolium enolates such as **4a**, **4b-Et/Me** and **4c***en route* to β-lactones/cyclopentenes had not been observed before. By employing the saturated NHC SIPr, we achieved sufficient stabilization of these intermediates such that the subsequent intramolecular aldol addition to the 5-membered carbocycles does not occur spontaneously at room temperature. However, as studied exemplarily with the Michael addition adducts **4b-Et** and **4c**, heating to 80 °C for 12 h in THF or toluene indeed resulted in the formation of the expected cyclopentene derivatives **5b-Et** and **5c**, along with the disappearance of the starting azolium enolates **4b-Et**,**c** (Scheme 4; see ESI† for NMR spectra).

#### γ-Butyrolactone formation with aldehydes

**Scheme 5:**
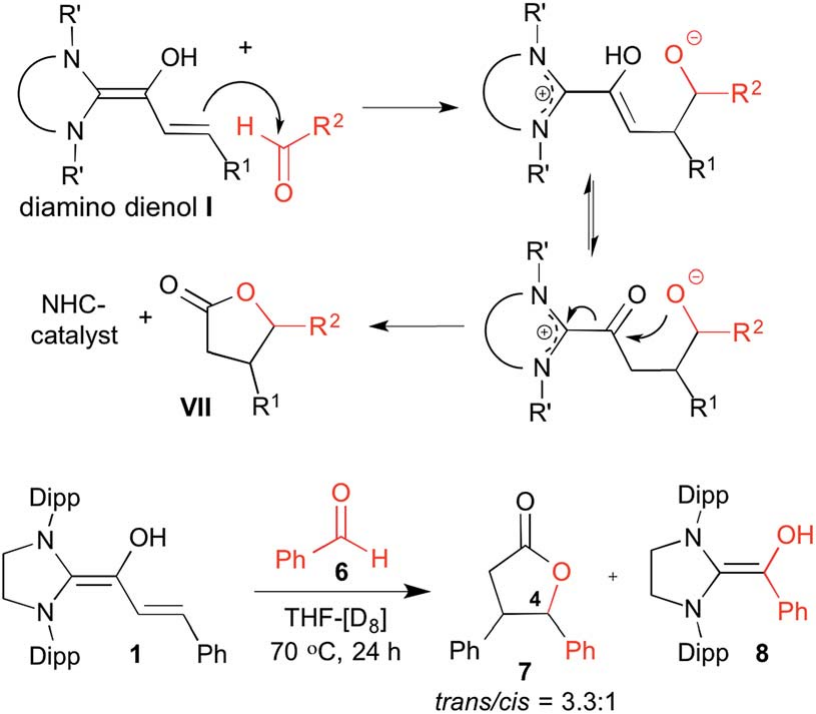
Top: general reaction scheme for the NHC-catalyzed formation of γ-butyrolactones **VII** from enals and aldehydes: bottom: diamino dienol **1** reacts with benzaldehyde (**6**) to afford the saturated lactone **7** and the diamino enol **8**; Dipp = 2,6-bis(2-propyl)phenyl.

Diamino dienols **I** have been postulated as intermediates in γ-butyrolactone (**VII**) formation from enals and aldehydes (Scheme 5, top).^5^ Exposition of the diamino dienol **1** to benzaldehyde (**6**) in THF at 70 °C indeed resulted in a slow conversion (*ca.* 50% after 24 h) to the saturated lactone **7** (*trans* : *cis* 3.3 : 1; Scheme 5, bottom). The most characteristic ^1^H NMR signals of **7** are a doublet at *δ* = 5.44 ppm [^3^*J*_*HH*_ = 9.0 Hz, 1H, H4 (*trans*)] and a doublet at *δ* = 5.85 ppm [^3^*J*_*HH*_ = 6.6 Hz, 1H, H4 (*cis*)]. In line with our earlier experience,^18^ the liberated NHC SIPr reacted with benzaldehyde to cleanly afford the diamino enol **8** (see ESI† for the NMR identification of lactone **7** and diamino enol **8**).

### Reactivity studies of azolium enolates

#### Formation of γ,δ-unsaturated δ-lactones with enones

**Scheme 6:**
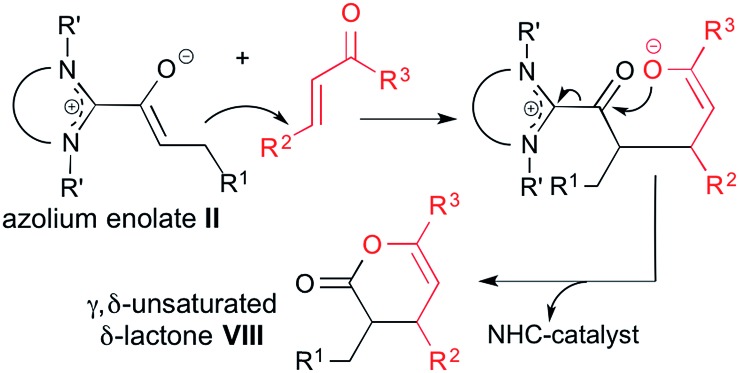
Reaction scheme for the NHC-catalyzed conversion of α,β-enals, *via* azolium enolates **II**, to γ,δ-unsaturated δ-lactones **VIII**.

As discussed above, the conversion of α,β-unsaturated aldehydes to cyclopentenes **VI** proceeds *via* initial diamino dienol formation and subsequent reaction of the latter with an enone electrophile (Scheme 2). In contrast, the conversion of α,β-unsaturated aldehydes with enones to γ,δ-unsaturated δ-lactones **VIII** (*i.e.* same starting materials, but different products) is assumed to involve additional tautomerization of the diamino dienol **I** to an azolium enolate **II** (see Scheme 1). The latter then reacts with the enone Michael acceptor, ultimately affording the γ,δ-unsaturated δ-lactone **VIII** (Scheme 6).

**Fig. 3:**
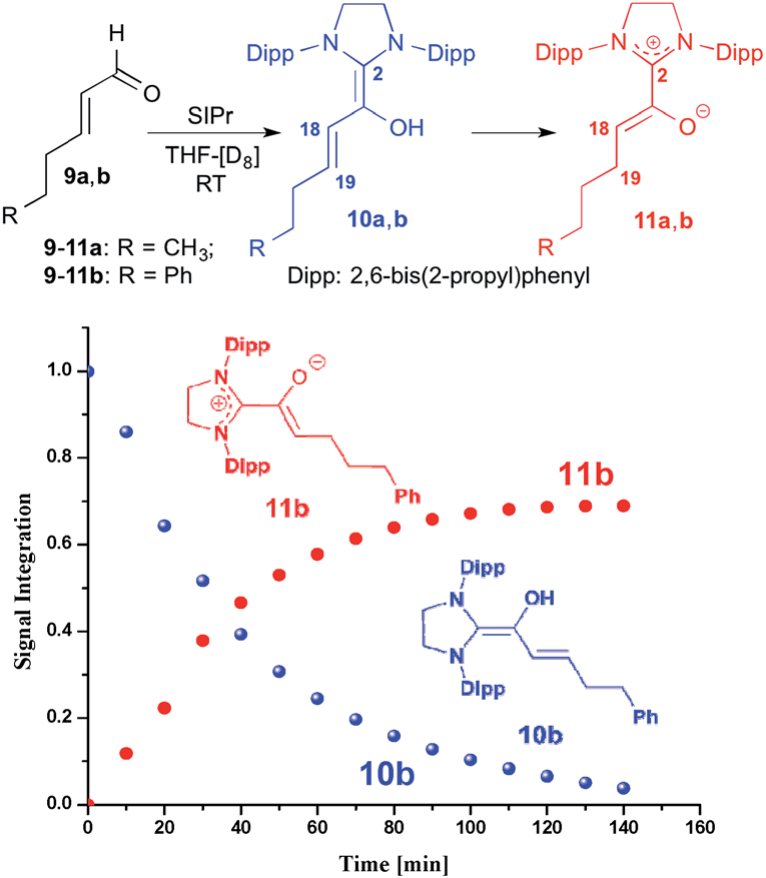
Top: generation of the azolium enolates **11a**,**b** from the enals **9a**,**b***via* diamino dienols **10a**,**b**: bottom: time course of the tautomerization of **10b** to the azolium enolate **11b**.

For studying the reactivity of preformed azolium enolates, we chose the two stable representatives **11a** and **11b** shown in Fig. 3 (top). Upon addition of *n*-hexenal (**9a**) to SIPr in THF-[D_8_] at room temperature, we observed the instantaneous disappearance of the aldehyde signal characteristic of **9a**, and the appearance of diamino dienol **10a**, as evidenced by a doublet at *δ* = 5.32 ppm (^3^*J*_*HH*_ = 12.0 Hz, 1H, H18), a multiplet at *δ* = 4.71–4.66 ppm (1H, H19) and singlet at *δ* = 3.40 ppm (OH). At room temperature, the diamino dienol **10a** tautomerized to the azolium enolate **11a** within *ca.* 20 min.^20^ The latter shows a characteristic ^1^H NMR triplet at *δ* = 3.46 ppm (^3^*J*_*HH*_ = 7.0 Hz, 1H, H18), and a multiplet at *δ* = 1.82–1.78 ppm, (2H, H19). Indicative ^13^C NMR resonances are those of C2 and C18, appearing at *δ* = 172.5 ppm and 100.5 ppm, respectively (see ESI† for further NMR data of **11a**).

In a similar manner, when we exposed *E*-5-phenylpent-2-enal (**9b**) to SIPr, ^1^H NMR monitoring first revealed the instantaneous formation of the diamino dienol **10b**, characterized by a doublet at *δ* = 5.39 ppm (^3^*J*_*HH*_ = 14.9 Hz, H18), a multiplet at *δ* = 4.80–4.75 ppm (H19), and a singlet at *δ* = 3.42 ppm (OH) (see ESI† for further NMR data of **11b**). After 10 min, the formation of the azolium enolate **11b** was noticeable, and its concentration increased over time (Fig. 3, bottom). The azolium enolate **11b** is characterized by a ^1^H NMR triplet at *δ* = 3.55 ppm (^3^*J*_*HH*_ = 7.1 Hz, 1H, H18), and a multiplet at *δ* = 1.89–1.86 ppm (2H, H19). In the ^13^C NMR spectrum, the formation of **11b** is evidenced by the characteristic signals of C2 and C18, appearing at *δ* = 172.4, 99.4 ppm respectively (see ESI† for further NMR data of **11b**). Note that in an earlier report from our laboratory, we had observed that diamino dienols derived from enals with additional conjugation (*e.g. E*-cinnamic aldehyde, sorbic aldehyde) do *not* undergo tautomerization to azolium enolates.18*b* Tautomerization occurs only in the absence of this additional conjugative stabilization of the diamino dienol state, for example with *E*-hexenal (**9a**) and *E*-5-phenylpent-2-enal (**9b**) as reported here, or with *E*-crotonic aldehyde as substrate aldehyde.18*b*

**Fig. 4:**
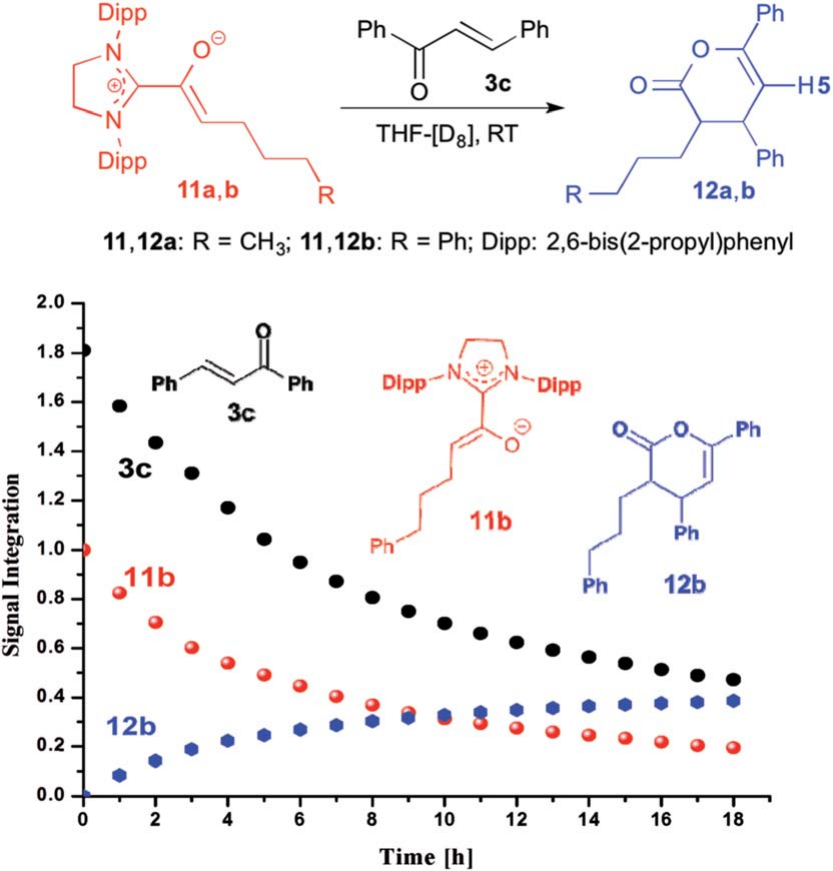
Top: formation of the γ,δ-unsaturated lactones **12a**,**b** from the azolium enolates **11a**,**b** and *E*-chalcone (**3c**); bottom: time course of the conversion of the azolium enolate **11b** to the γ,δ-unsaturated lactone **12b**.

When we added *E*-chalcone (**3c**) to the pre-formed azolium enolate **11b**, the concentrations of both **11b** and **3c** decreased simultaneously over time (Fig. 4), along with the appearance of the unsaturated δ-lactone **12b** (*trans* : *cis* 5.8 : 1). The latter is characterized by a ^1^H NMR doublet at *δ* = 5.97 ppm [^3^*J*_*HH*_ = 4.4 Hz, 1H, H5 (*trans*)] and a doublet at *δ* = 6.21 ppm [^3^*J*_*HH*_ = 6.5 Hz, 1H, H5 (*cis*)] (see ESI† for the NMR identification of the lactone **12b**). In the case of the azolium enolate **11a**, reaction with *E*-chalcone (**3c**) gave the analogous unsaturated δ-lactone **12a** (*trans* : *cis* 11 : 1), characterized by a ^1^H NMR doublet at *δ* = 5.98 ppm [^3^*J*_*HH*_ = 4.4 Hz, 1H, H5 (*trans*)] and a doublet at *δ* = 6.22 ppm [^3^*J*_*HH*_ = 6.6 Hz, 1H, H5 (*cis*)] (see ESI† for the full NMR identification of **12a**).

## Conclusion

We have reported (i) the selective generation and characterization of a number of hitherto postulated diamino dienol and azolium enolate reaction intermediates, by interaction of the N-heterocyclic carbene SIPr with various α,β-unsaturated aldehydes. (ii) The homoenolate and enolate equivalents thus prepared were stable enough for NMR-spectroscopic characterization, but still reactive enough for further transformations when exposed to electrophilic reaction partners: exposure of diamino dienols to Michael acceptors gave hitherto postulated addition products stable enough for NMR and even X-ray crystallographic characterization. Heating of the latter completed the reaction cycle, affording trisubstituted cyclopentenes. (iii) In the same manner, the postulated reaction of diamino dienol intermediates with aldehydes to γ-butyrolactones could be verified experimentally. (iv) The tautomerization of primarily formed diamino dienols to azolium enolates, the postulated precursors of γ,δ-unsaturated δ-lactones, was monitored by ^1^H NMR in two cases. Subsequent exposure of the azolium enolates to *E*-chalcone as Michael acceptor indeed gave the corresponding γ,δ-unsaturated δ-lactones, thus proving the postulated C–C bond formation at Cβ of the azolium enolate intermediate. We are convinced that the mechanistic information disclosed herein will promote the understanding of other existing NHC-catalyzed transformations, and the design of novel ones.

## Supplementary Material

Supplementary information

Crystal structure data
