# Supplementary material for: Carbene catalyzed umpolung of α,β-enals: a reactivity study of diamino dienols vs. azolium enolates, and the characterization of advanced reaction intermediates
Source: Chem Sci. 2015 Apr 30;6(7):3706–11. doi: 10.1039/c5sc01027f (PMC5496186; doi:10.1039/c5sc01027f)
Supplement: Supplementary file 1 [file SC-006-C5SC01027F-s001.pdf]

Electronic Supplementary Material (ESI) for Chemical Science.
This journal is © The Royal Society of Chemistry 2015

Supporting Information

1 **General** 3

2 **Synthesis of *E*-5-phenylpent-2-enal (9b)** 4

3 **Synthesis of methyl *E*-3-benzoylacrylate (3b-Me)** 4

4 ***In situ* generation of azolium enolates (11a-b)** 5

5 **Generation and characterization of Michael addition products** 7

5.1 **Reaction of the 2,2-diamino dienol 1 with an equimolar amount of methyl *E*-4-oxo-2-pentenoate (3a)** 7

5.2 **Reaction of the 2,2-diamino dienol 1 with an equimolar amount of ethyl-*E*-3-benzoylacrylate (3b-Et)** 8

5.3 **Reaction of the 2,2-diamino dienol 1 with an equimolar amount of *E*-chalcone (3c)** 9

5.4 **Reaction of the 2,2-diamino dienol 1 with an equimolar amount of methyl-*E*-3-benzoylacrylate (3b-Me)** 10

6 **NMR studies of cyclopentene formation from the Michael addition products** 11

7 **NMR studies of γ-butyrolactone formation from pre-formed 2,2-diamino dienol** 13

7.1 **Reaction of the 2,2-diamino dienol 1 with an equimolar amount of benzaldehyde (6)** 13

8 **NMR studies of γ,δ-unsaturated δ-lactone formation from pre-formed azolium enolates** 15

8.1 **Reaction of the azolium enolate 11a with an equimolar amount of *E*-chalcone (3c)** 15

8.2 **Reaction of the azolium enolate 11b with *E*-chalcone (3c)** 18

9 **Independent synthesis of cyclopentene products** 19

9.1 **Synthesis of *rac*-ethyl (1*R*,2*R*)-2,4-diphenylcyclopent-3-en-1-carboxylate (5b-Et)** 19

9.2 **Synthesis of *rac*-1,1',1''-[(*1R*,*2R*)-cyclopent-3-ene-1,2,4-triyl]tribenzene (5c)** 20

10 **Independent synthesis of *rac*-(4*R*,5*S*)-4,5-diphenyloxolan-2-one (7)** 20

11 **Isolation of of γ,δ-unsaturated δ-lactone products** 21

11.1 **Synthesis of *rac*-(*3R,4S*)-3-butyl-4,6-diphenyl-3,4-dihydro-2*H*-pyran-2-one (12a)** 21

11.2 **Synthesis of *rac*-(3*R*,4*S*)-4,6-diphenyl-3-(3-phenylpropyl)-3,4-dihydro-2*H*-pyran-2-one (12b)** 22

12 NMR spectra ... 23

12.1 ^{1}H and ^{13}C NMR spectra of the *E*-5-phenylpent-2-enal (9b) ... 23

12.2 ^{1}H and ^{13}C NMR spectra of the methyl *E*-3-benzoylacrylate (3b-Me) ... 24

12.3 1D and 2D NMR spectra of the azolium enolate 11a ... 25

12.4 ^{1}H and ^{13}C NMR spectra of the 2,2-diamino dienol 10b ... 29

12.5 1D and 2D NMR spectra of the azolium enolate 11b ... 30

12.6 ^{1}H NMR spectrum of the 2,2-diamino dienol 1 ... 35

12.7 1D and 2D NMR spectra of the Michael addition product 4a ... 36

12.8 1D and 2D NMR spectra of the Michael addition product 4b-Et ... 42

12.9 1D and 2D NMR spectra of the Michael addition product 4c ... 47

12.10 1D and 2D NMR spectra of the Michael addition product 4b-Me ... 52

12.11 ^{1}H and ^{13}C NMR spectra of *rac*-ethyl (1*R*,2*R*)-2,4-diphenylcyclopent-3-en-1-carboxylate (5b-Et) ... 57

12.12 ^{1}H and ^{13}C NMR spectra of *rac*-1,1',1''-[(*1R*,*2R*)-cyclopent-3-ene-1,2,4-triyl]tribenzene (5c) ... 58

12.13 ^{1}H and ^{13}C NMR spectra of *rac*-(4*R*,5*S*)-4,5-diphenyloxolan-2-one (7) ... 59

12.14 1D and 2D NMR spectra of *rac*-(*3R,4S*)-3-butyl-4,6-diphenyl-3,4-dihydro-2*H*-pyran-2-one (12a) ... 60

12.15 ^{1}H and ^{13}C NMR spectra of *rac*-(3*R*,4*S*)-4,6-diphenyl-3-(3-phenylpropyl)-3,4-dihydro-2*H*-pyran-2-one (12b) ... 65

13 X-ray data of compounds 4b-Et and 4b-Me: Crystal data and structure refinement, selected geometric data, ORTEPs (Oak Ridge Thermal Ellipsoid Plot). ... 66

13.1 X-ray data of 4b-Et ... 66

13.2 X-ray data of 4b-Me ... 69

14 References ... 72

1 General

1,3-Bis(2,6-diisopropylphenyl)imidazolidin-2-ylidene (>98.0 %, SIPr (**9**)) was purchased from TCI. *E*-2-Hexenal, methyl *E*-4-oxo-2-pentenoate, ethyl *E*-3-benzoylacrylate and *E*-chalcone were purchased from Sigma-Aldrich. (Triphenylphosphoranylidene)acetaldehyde and hydrocinnamaldehyde were purchased from Alfa Aesar. Aldehydes were distilled and stored in a glovebox. Benzene was dried over sodium and degassed by several *freeze-pump-thaw* cycles prior to use. [D_8]THF and [D_6]benzene were passed through neutral aluminium oxide (*Brockmann* activity 1), degassed by several *freeze-pump-thaw* cycles and stored over 4 Å molecular sieves in a glovebox. All reactions were performed under argon atmosphere in a glovebox. Nuclear magnetic resonance (NMR) spectra were recorded on a *Bruker* Avance II 600 instrument (^{1}H: 600.20 MHz, ^{13}C: 150.92 MHz). Spectra were recorded at room temperature unless otherwise stated. Chemical shifts (δ) are reported in parts per million relative to tetramethylsilane (TMS) or solvent residual signals. The following abbreviations were used for chemical shift multiplicities in ^{1}H NMR spectra: br s = broad signal, s = singlet, d = doublet, dd = doublet of doublets, t = triplet, td = triplet of doublets, q = quartet, quint = quintet, sept = septet, sext = sextet, m = multiplet. NMR signals were assigned by evaluation of 1D and 2D NMR data (^{1}H,^{1}H COSY, ^{1}H,^{1}H NOESY, ^{1}H,^{13}C HMQC, ^{1}H,^{13}C HMBC).

2 Synthesis of *E*-5-phenylpent-2-enal (9b)

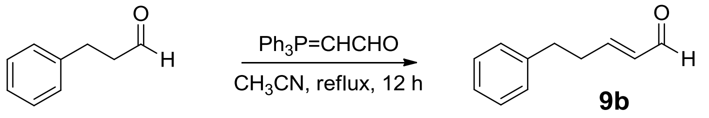

A mixture of 2-(triphenylphosphoranylidene)acetaldehyde (2.0 g, 6.57 mmol, 1.0 equiv) and hydrocinnamaldehyde (882 mg, 6.57 mmol, 1.0 equiv) in $CHCl_3$ (30 ml) was refluxed for 12 h under argon atmosphere. The resulting solution was cooled to room temperature, concentrated and chromatographed on silica gel eluting with cyclohexane/ethyl acetate (3:1) to give *E*-5-phenylpent-2-enal[1] as light yellow liquid (0.526 mg, 3.29 mmol, 50 % yield).

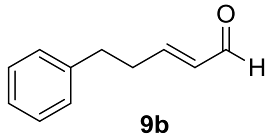

9b

^{1}H NMR (600 MHz, [D$_8$]THF): δ 9.49 (d, 1H, ^{3}J = 7.7 Hz, -CHO), 7.30-7.26 (m, 2H, Ar), 7.24-7.23 (m, 2H, Ar), 7.20-7.17 (m, 1H, Ar), 6.94-6.89 (m, 1H, =CH-), 6.13-6.08 (m, 1H, =CH-), 2.85 (t, 2H, ^{3}J = 7.9 Hz, -CH_2), 2.69-2.66 (m, 2H, -CH_2). **^{13}C NMR (150 MHz, [D$_8$]THF): δ** 192.2, 156.2, 140.8, 133.4, 128.2, 128.1, 125.9, 34.1, 34.0.

3 Synthesis of methyl *E*-3-benzoylacrylate (3b-Me)

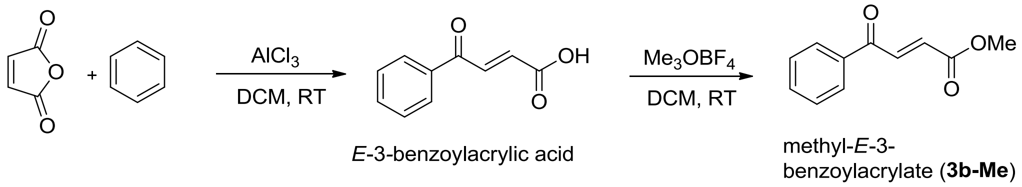

E-3-benzoylacrylic acid was synthesized according to a literature protocol.[2] The addition of Meerwein's reagent (trimethyloxonium tetrafluoroborate) to this acid in DCM afforded methyl *E*-3-benzoylacrylate, and analytical data are consistent with published ones.[3]

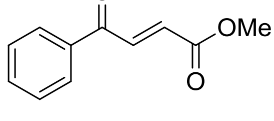

3b-Me

^{1}H NMR (600 MHz, $CDCl_3$): δ 8.02-8.01 (m, 2H, Ar), 7.93 (d, 1H, ^{3}J = 15.6 Hz), 7.65-7.63 (m, 1H, Ar), 7.54-7.52 (m, 2H, Ar), 6.90 (d, ^{3}J = 15.6 Hz), 3.90 (s, 3H). **^{13}C NMR (150 MHz, $CDCl_3$): δ** 189.42, 166.02, 136.61, 133.90, 132.07, 128.91, 128.87, 52.36. **HR-MS** (EI) [M] *m/z* calcd for [$C_{11}H_{10}O_3$] [M] 190.0630 found 190.063.

4 *In situ* generation of azolium enolates (11a-b)

11a: (1*Z*)-1-{1,3-bis[2,6-di(propan-2-yl)phenyl]-4,5-dihydro-1*H*-imidazol-3-ium-2-yl}hex-1-en-1-olate

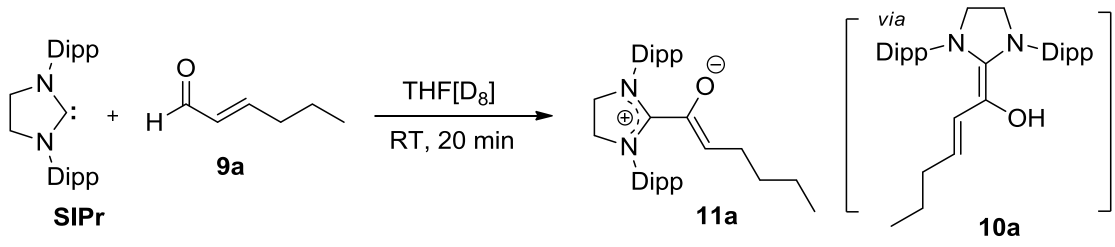

In a glovebox, an NMR tube was charged with 20 mg (51 µmol, 1.0 equiv) of SiPr in [D$_8$]THF and sealed with a septum. 1.0 Equiv of *E*-2-hexenal (5.0 mg, 6.0 µl) was added with a syringe and the reaction was followed by NMR spectroscopy. In this case, first the formation of the 2,2-diamino dienol **10a** (see figure S18 (top) for ^{1}H NMR) was observed. The latter was converted to the azolium enolate **11a** within ca. 20 min (see figure S19-S26 for 1D and 2D NMR of **11a**).

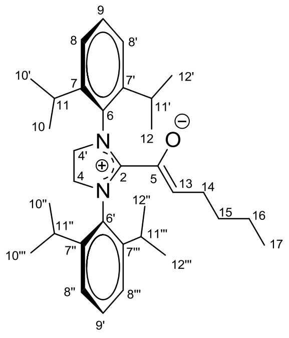

11a

^{1}H NMR (600 MHz, [D$_8$]THF): δ 7.31-7.28 (m, 2H, H9, H9’), 7.22-7.20 (m, 4H, H8, H8’, H8”, H8’”), 4.09 (s, 4H, H4, H4’), 3.46 (t, 1H, H13, ^{3}J = 6.9 Hz), 3.23 (sept, 4H, ^{3}J = 6.7 Hz, H11, H11’, H11”, H11’”), 1.81-1.78 (m, 2H, H14), 1.34 (d, 12H, ^{3}J = 6.9 Hz, H10, H10’, H10”, H10’”), 1.32 (d, 12H, ^{3}J = 6.9 Hz, H12, H12’, H12”, H12’”), 0.91 (quint, 2H, H15, ^{3}J = 7.2 Hz), 0.82 (sext, 2H, H16, ^{3}J = 7.2 Hz), 0.63 (t, 3H, H17, ^{3}J = *7.2* Hz). **^{13}C NMR (150 MHz, [D$_8$]THF):** δ 172.5 (1C, C2), 148.9 (1C, C5), 145.6 (4C, C7, C7’, C7”, C7’”), 135.0 (2C, C6, C6’), 128.4 (2C, C9, C9’), 123.9 (4C, C8, C8’, C8”, C8’”), 100.5 (1C, C13), 52.3 (2C, C4, C4’), 32.6 (1C, C15), 28.9 (4C, C11, C11’, C11”, C11’”), 24.4 (4C, C10, C10’, C10”, C10’”), 24.2 (1C, C14), 23.7 (4C, C12, C12’, C12”, C12’”), 21.8 (1C, C16), 13.5 (1C, C17).

11b: (1*Z*)-1-{1,3-bis[2,6-di(propan-2-yl)phenyl]-4,5-dihydro-1*H*-imidazol-3-ium-2-yl}-5-phenylpent-1-en-1-olate

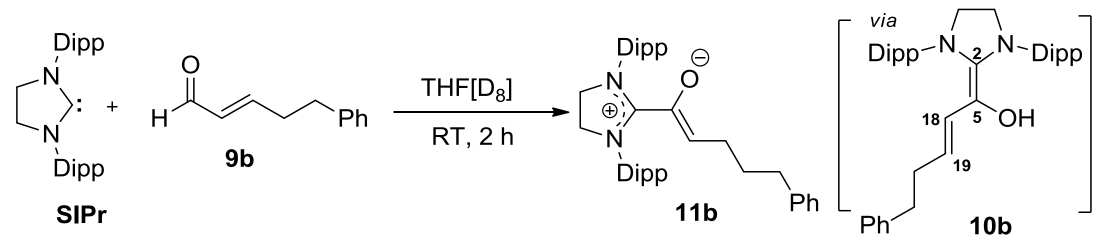

In a glovebox, an NMR tube was charged with 20 mg (51 µmol, 1.0 equiv) of SiPr in [D$_8$]THF and sealed with a septum. 1.0 Equiv of *E*-5-phenylpent-2-enal (**9b**) (8.2 mg, 51 µmol) was added and the reaction was followed by NMR spectroscopy. In this case, first the formation of the 2,2-diamino dienol **10b** (see figure S27-S28 for 1D NMR) was observed. The latter was converted to the azolium enolate **11b** within 2 h (see figure S29-S38 for 1D and 2D NMR of **11b**).

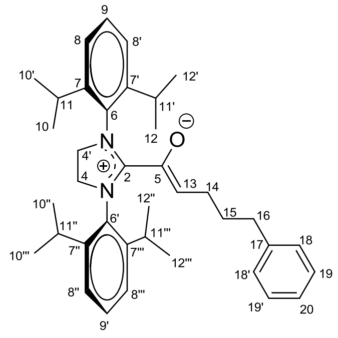

11b

^{1}H NMR (600 MHz, [D$_8$]THF): δ 7.31-7.30 (m, 2H, H9, H9'), 7.29-7.22 (m, 4H, H8, H8', H8'', H8'''), 7.13-7.10 (m, 2H, H19, H19'), 7.03-7.01 (m, 1H, H20), 6.94-6.93 (m, 2H, H18, H18'), 4.12 (s, 4H, H4, H4'), 3.55 (t, 1H, H13, ^3J = 7.0 Hz), 3.26 (sept, 4H, ^3J = 6.7 Hz, H11, H11', H11'', H11'''), 2.05 (t, 2H, ^3J = 7.8 Hz, H16), 1.89-1.86 (m, 2H, H14), 1.40 (d, 12H, ^3J = 6.7 Hz, H12, H12', H12'', H12'''), 1.34 (d, 12H, ^3J = 6.7 Hz, H10, H10', H10'', H10'''), 1.27-1.25 (m, 2H, H15). **^{13}C NMR (150 MHz, [D$_8$]THF): δ** 172.4 (1C, C2), 149.3 (1C, C5), 145.7 (4C, C7, C7', C7'', C7'''), 143.9 (1C, C17), 134.9 (2C, C6, C6'), 128.6 (2C, C9, C9'), 128.3 (2C, C18, C18'), 127.4 (2C, C19, C19'), 124.5 (1C, C20), 124.0 (4C, C8, C8', C8'', C8'''), 99.4 (1C, C13), 52.3 (2C, C4, C4'), 35.3 (1C, C16), 32.7 (1C, C15), 28.9 (4C, C11, C11' C11'', C11'''), 24.5 (1C, C14), 24.4 (4C, C12, C12' C12'', C12'''), 23.7 (4C, C10, C10' C10'', C10''').

5 Generation and characterization of Michael addition products

5.1 Reaction of the 2,2-diamino dienol 1 with an equimolar amount of methyl *E*-4-oxo-2-pentenoate (3a)

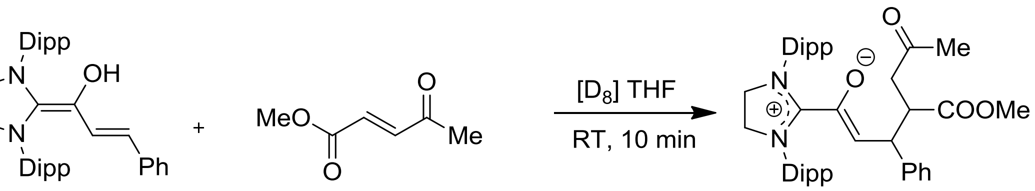

1 **3a** **4a**

In a glovebox, an NMR tube was charged with 15 mg (38 µmol, 1.0 equiv) of SiPr in [D$_8$] THF and sealed with a septum. 1.0 Equiv of *E*-cinnamic aldehyde (5.1 mg, 4.8 µl) was added with a syringe, followed by measuring ^{1}H NMR showing the signals of **1** (see figure S39). Then 1.0 equiv of methyl *E*-4-oxo-2-pentenoate **3a** (4.92 mg, 38 µmol) was added and the reaction was followed by NMR. The reaction was completed within 10 min, affording the Michael addition product **4a** (see figure S40-S50 for 1D and 2D NMR).

4a: ***rac*-(1*Z*,3*R*,4*Ξ*)-1-{1,3-bis[2,6-di(propan-2-yl)phenyl]-4,5-dihydro-1*H*-imidazol-3-ium-2-yl}-6-methyl-4-(methoxycarbonyl)-6-oxo-3-phenylhex-1-en-1-olate**

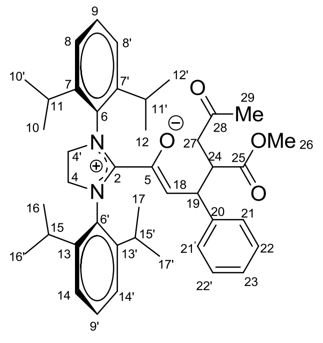

4a

^{1}H NMR (600 MHz, [D$_8$]THF): δ 7.19-7.17 (m, 2H, H9, H9'), 7.08-7.06 (m, 2H, H8, H8'), 7.05-7.03 (m, 2H, H14, H14'), 6.74-6.72 (m, 3H, H22, H22', H23), 6.64-6.63 (m, 2H, H21, H21'), 4.12-4.07 (m, 2H, H4), 4.04-3.98 (m, 2H, H4'), 3.36-3.30 (m, 2H, H18, H19), 3.13 (sept, 2H, ^{3}J = 6.6 Hz, H15, H15'), 3.07 (sept, 2H, ^{3}J = 6.6 Hz, H11, H11'), 2.85 (s, 3H, H26), 2.73 (td, 1H, H24, $^{3}J_{H24\text{-}H19}$ = $^{3}J_{H24\text{-}H27b}$ = 11.4 Hz, $^{3}J_{H24\text{-}H27a}$ = 3.0 Hz), 2.25 (dd, 1H, H27b, $^{3}J_{H27b\text{-}H24}$ = 11.4 Hz, $^{2}J_{H27b\text{-}H27a}$ = 17.4 Hz), 1.86 (dd, 1H, H27a, $^{3}J_{H27a\text{-}H24}$ = 3.0 Hz, $^{2}J_{H27a\text{-}H27b}$ = 17.4 Hz), 1.62 (s, 3H, H29), 1.23 (d, 6H, ^{3}J = 6.6 Hz, H17, H17'), 1.16 (d, 6H, ^{3}J = 6.6 Hz, H16, H16'), 1.14 (d, 6H, ^{3}J = 6.6 Hz, H10, H10'), 1.11 (d, 6H, ^{3}J = 6.6 Hz, H12, H12'). **^{13}C NMR (150 MHz, [D$_8$]THF): δ** 205.7 (1C, C28), 174.7 (1C, C25), 171.3 (1C, C2), 149.4 (1C, C5), 146.6 (1C, C20), 146.3 (2C, C13, C13'), 146.0 (2C, C7, C7'), 133.4 (2C, C6, C6'), 129.2 (2C, C9, C9'), 127.8 (2C, C21, C21'), 126.9 (2C, C22, C22'), 124.3 (2C, C8, C8'), 124.2 (2C, C14, C14'), 124.1 (1C, C23), 96.0 (1C, C18), 52.0 (2C, C4, C4'), 49.3 (1C, C26), 47.8 (1C, C24), 45.2 (1C, C27), 44.6 (1C, C19), 29.1 (2C, C15, C15'), 28.9 (2C, C11, C11'), 28.6 (1C, C29), 24.9 (4C, C10, C10', C16, C16'), 23.4 (2C, C12, C12'), 23.2 (2C, C17, C17').

5.2 Reaction of the 2,2-diamino dienol 1 with an equimolar amount of ethyl *E*-3-benzoylacrylate (3b-Et)

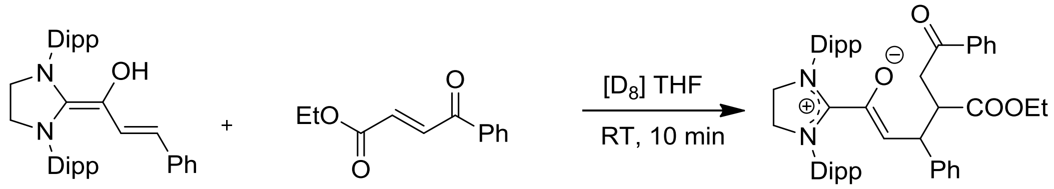

1 **3b-Et** **4b-Et**

In a glovebox, an NMR tube was charged with 12 mg (31 µmol, 1.0 equiv) of SiPr in [D$_8$] THF and sealed with a septum. 1.0 Equiv of *E*-cinnamic aldehyde (4.1 mg, 3.9 µl) was added with a syringe, followed by measuring ^{1}H NMR, showing the signals of **1** (see figure S39). Then 1.0 equiv of ethyl-*E*-3-benzoylacrylate **3b-Et** (6.27 mg, 5.6 µl) was added with a syringe and the reaction was followed by NMR. The reaction was completed within 10 min, affording the Michael addition product **4b-Et** (see figure S51-S60 for 1D and 2D NMR).

4b-Et: *rac*-(1*Z*,3*R*,4*Ξ*)-1-{1,3-bis[2,6-di(propan-2-yl)phenyl]-4,5-dihydro-1*H*-imidazol-3-ium-2-yl}-4-(ethoxycarbonyl)-6-oxo-3,6-diphenylhex-1-en-1-olate

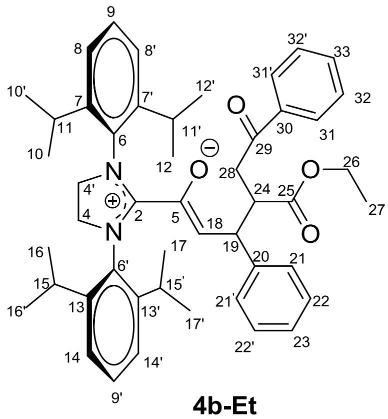

4b-Et

^{1}H NMR (600 MHz, [D$_8$]THF): δ 7.81-7.80 (m, 2H, H31, H31'), 7.55-7.52 (m, 1H, H33), 7.44-7.41 (m, 2H, H32, H32'), 7.11-7.08 (m, 4H, H9, H9', H8, H8'), 7.04-7.02 (m, 2H, H14, H14'), 6.94-6.90 (m, 3H, H22, H22', H23), 6.85-6.84 (m, 2H, H21, H21'), 4.24-4.12 (m, 4H, H4, H4'), 3.70 (dd, 1H, $^3J_{H19\text{-}H18}$ = 8.9 Hz, $^3J_{H19\text{-}H24}$ = 11.2 Hz, H19), 3.52 (d, 1H, ^3J = 8.9 Hz, H18), 3.49-3.42 (m, 2H, H26), 3.28-3.24 (m, 3H, H11, H11', H28b), 3.19 (sept, 2H, ^3J = 6.6 Hz, H15, H15'), 2.98 (td, 1H, H24, $^3J_{H24\text{-}H28b}$ = $^3J_{H24\text{-}H19}$ = 11.2 Hz, $^3J_{H24\text{-}H28a}$ = 2.6 Hz), 2.62 (dd, 1H, $^3J_{H28a\text{-}H24}$ = 2.6 Hz, $^3J_{H28a\text{-}H28b}$ = 18.1 Hz, H28a), 1.37 (d, 6H, ^3J = 6.6 Hz, H17, H17'), 1.28 (d, 6H, ^3J = 6.6 Hz, H10, H10'), 1.25 (d, 6H, ^3J = 6.6 Hz, H16, H16'), 1.22 (d, 6H, ^3J = 6.6 Hz, H12, H12'), 0.61 (t, 3H, H27, ^3J = 7.2 Hz).**^{13}C NMR (150 MHz, [D$_8$]THF): δ** 198.4 (1C, C29), 174.1 (1C, 25), 171.5 (1C, C2), 149.5 (1C, C5), 146.2 (2C, C13, C13'), 145.9 (2C, C7, C7'), 145.8 (1C, C20), 137.5 (1C, C30), 133.5 (2C, C6, C6'), 131.8 (1C, C33), 129.0 (2C, C9, C9'), 128.2 (2C, C31, C31'), 128.1 (2C, C21, C21') 127.8 (2C, C32, C32'), 126.9 (2C, C22, C22'), 124.3 (1C, C23), 124.1 (2C, C8, C8'), 124.0 (C14, C14'), 98.3 (1C, C18), 58.2 (1C, C26), 52.0 (2C, C4, C4'), 48.1 (1C, C24), 44.7 (1C, C19), 40.8 (1C, C28), 29.0 (2C, C15, C15'), 28.9 (2C, C11, C11'), 24.83 (2C, C16, C16'), 24.79 (2C, C10, C10'), 23.4 (2C, C17, C17'), 23.3 (2C, C12, C12'), 13.0 (1C, C27).

5.3 Reaction of the 2,2-diamino dienol 1 with an equimolar amount of *E*-chalcone (3c)

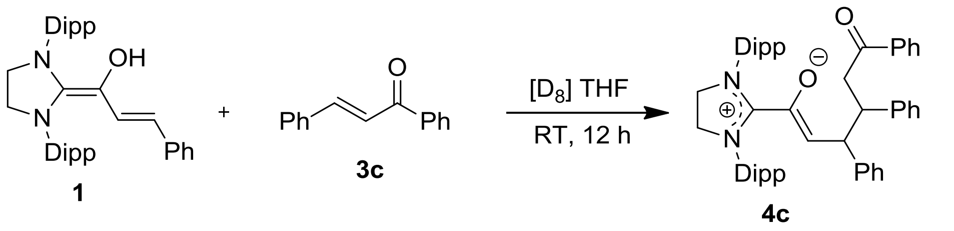

In a glovebox, an NMR tube was charged with 15 mg (38 µmol, 1.0 equiv) of SiPr in [D$_8$] THF and sealed with a septum. 1.0 Equiv of *E*-cinnamic aldehyde (5.08 mg, 4.8 µl) was added with a syringe, followed by measuring ^{1}H NMR, showing the signals of **1** (see figure S39). Then 1.0 equiv of *E*-chalcone **3c** (8.0 mg, 38 µmol) was added and the reaction was followed by NMR. After 12 h, 80% of *E*-chalcone was consumed, affording the Michael addition product **4c** (see figure S61-S70 for 1D and 2D NMR).

4c: ***rac*-(1*Z*,3*R*,4*Ξ*)-1-{1,3-bis[2,6-di(propan-2-yl)phenyl]-4,5-dihydro-1*H*-imidazol-3-ium-2-yl}-6-oxo-3,4,6-triphenylhex-1-en-1-olate**

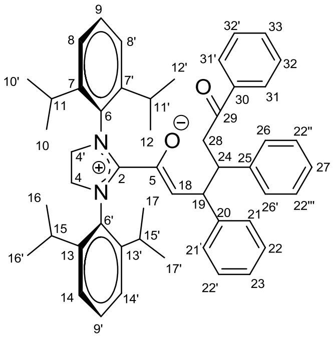

4c

^{1}H NMR (600 MHz, [D$_8$]THF): δ 7.74-7.72 (m, 2H, H31, H31'), 7.47-7.45 (m, 1H, H33), 7.36-7.34 (m, 2H, H32, H32'), 7.14-7.13 (m, 2H, H8, H8'), 7.08-7.06 (m, 2H, H9, H9'), 7.0-6.99 (m, 2H, H14, H14'), 6.89-6.88 (m, 2H, H26, H26'), 6.79-6.76 (m, 2H, H22'', H22'''), 6.74-6.73 (m, 2H, H22, H22'), 6.70-6.69 (m, 2H, H23, H27), 6.6-6.65 (m, 2H, H21, H21'), 4.23-4.17 (m, 2H, H4), 4.15-4.11 (m, 2H, H4'), 3.96 (dd, 1H, H19, $^3J_{H19\text{-}H18}$ = 9.0 Hz, $^3J_{H19\text{-}H24}$ = 11.4 Hz), 3.67 (d, 1H, H18, $^3J_{H18\text{-}H19}$ = 9.0 Hz), 3.39 (td, 1H, H24, $^3J_{H24\text{-}H19}$ = $^3J_{H24\text{-}H28b}$ = 11.4 Hz, $^3J_{H24\text{-}H28a}$ = 2.4 Hz), 3.32 (sept, 2H, $^3J_{HH}$ = 6.6 Hz, H11, H11'), 3.23-3.17 (m, 3H, H15, H15', H_{28b}), 2.77 (dd, 1H, H28a, $^3J_{H28a\text{-}H24}$ = 2.4 Hz, $^3J_{H28a\text{-}H28b}$ = 17.4 Hz), 1.47 (d, 6H, $^3J_{HH}$= 6.6 Hz, H12, H12'), 1.31 (d, 6H, $^3J_{HH}$= 6.6 Hz, H16, H16'), 1.24 (d, 6H, $^3J_{HH}$= 6.6 Hz, H10, H10'), 1.17 (d, 6H, $^3J_{HH}$= 6.6 Hz, H17, H17').**^{13}C NMR (150 MHz, [D$_8$]THF): δ** 198.6 (1C, C29), 171.8 (1C, C2), 149.3 (1C, C5), 147.0 (1C, C20), 146.2 (2C, C7, C7'), 145.9 (2C, C13, C13'), 145.2 (1C, C25), 138.1 (1C, C30), 133.6 (2C, C6, C6'), 131.4 (1C, C33), 129.0 (2C, C9, C9'), 128.4 (2C, C26, C26'), 128.2 (2C, C21, C21'), 128.0 (2C, C31, C31'), 127.6 (C32, C32'), 126.7 (4C, C22, C22', C22'', C22'''), 124.3 (2C, C14, C14'), 124.1 (1C, C27), 124.0 (2C, C8, C8'), 123.4 (1C, C23), 100.4 (1C, C18), 52.0 (2C, C4, C4'), 48.0 (1C, C24), 47.0 (1C, C19), 44.4 (1C, C28), 29.1 (2C, C11, C11'), 28.9 (2C, C15, C15'), 24.8 (2C, C10, C10'), 24.78 (2C, C16, C16'), 23.35 (2C, C17, C17'), 23.33 (2C, C12, C12').

5.4 Reaction of the 2,2-diamino dienol 1 with an equimolar amount of methyl *E*-3-benzoylacrylate (3b-Me)

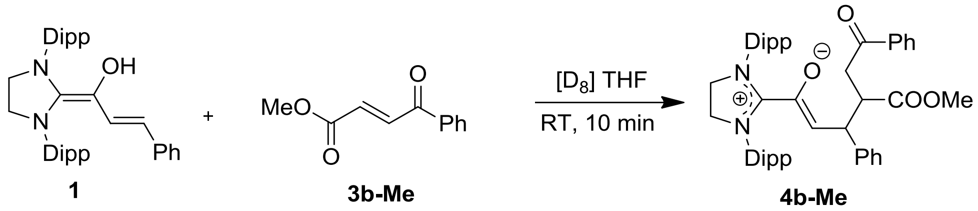

In a glovebox, an NMR tube was charged with 16 mg (41 µmol, 1.0 equiv) of SiPr in [D_8] THF and sealed with a septum. 1.0 Equiv of *E*-cinnamic aldehyde (5.4 mg, 5.2 µl) was added with a syringe, followed by measuring ^{1}H NMR, showing the signals of **1** (see figure S39). 1.0 Equiv of methyl-*E*-3-benzoylacrylate **3b-Me** (7.8 mg, 41 µmol) was added, and the reaction was followed by NMR. The reaction was completed within 10 min, affording the Michael addition product **4b-Me** (see figure S71-80 for 1D and 2D NMR).

4b-Me: *rac*-(1*Z*,3*R*,4*Ξ*)-1-{1,3-bis[2,6-di(propan-2-yl)phenyl]-4,5-dihydro-1*H*-imidazol-3-ium-2-yl}-4-(methoxycarbonyl)-6-oxo-3,6-diphenylhex-1-en-1-olate

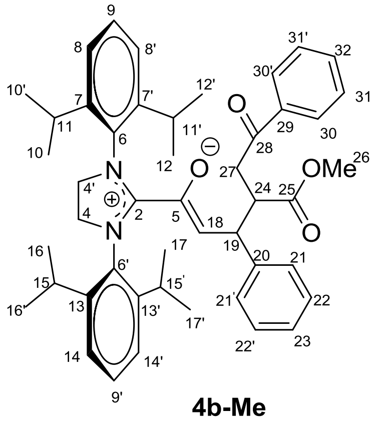

4b-Me

^{1}H NMR (600 MHz, [D_8]THF): δ 7.80-7.79 (m, 2H, H30, H30'), 7.56-7.52 (m, 1H, H32), 7.44-7.41 (m, 2H, H31, H31'), 7.11-7.09 (m, 4H, H9, H9', H8, H8'), 7.05-7.03 (m, 2H, H14, H14'), 6.94-6.90 (m, 3H, H22, H22', H23), 6.82-6.81 (m, 2H, H21, H21'), 4.22-4.12 (m, 4H, H4, H4'), 3.71-3.68 (m, 1H, H19), 3.52 (d, 1H, H18, $^3J_{HH}$ = 8.7 Hz), 3.30-3.20 (m, 5H, H11, H11', H15, H15' H27b), 3.0 (td, 1H, H24, $^3J_{H24\text{-}H27b}$ = $^3J_{H24\text{-}H19}$ = 11.0 Hz, $^3J_{H24\text{-}H27a}$ = 2.5 Hz), 2.97 (s, 3H, H26), 2.60 (dd, 1H, $^3J_{H27a\text{-}H24}$ = 2.5 Hz, $^3J_{H27a\text{-}H27b}$ = 18.0 Hz, H27a,), 1.37 (d, 6H, ^3J = 6.6 Hz, H17, H17'), 1.29 (d, 6H, ^3J = 6.6 Hz, H10, H10'), 1.26 (d, 6H, ^3J = 6.6 Hz, H16, H16'), 1.24 (d, 6H, ^3J = 6.6 Hz, H12, H12'). **^{13}C NMR (150 MHz, [D_8]THF): δ** 198.4 (1C, C28), 174.62 (1C, 25), 171.45 (1C, C2), 149.65 (1C, C5), 146.2 (2C, C13, C13'), 145.98 (2C, C7, C7'), 145.81 (1C, C20), 137.43 (1C, C29), 133.48 (2C, C6, C6'), 131.8 (1C, C32), 129.05 (2C, C9, C9'), 128.19 (2C, C30, C30'), 127.91 (2C, C21, C21'), 127.78 (2C, C31, C31'),126.90 (2C, C22, C22'), 124.32 (1C, C23), 124.12 (2C, C8, C8'), 124.02 (C14, C14'), 97.5 (1C, C18), 52.02 (2C, C4, C4'), 49.25 (1C, C26), 48.23 (1C, C24), 44.69 (1C, C19), 40.78 (1C, C27), 28.99 (2C, C11, C11'), 28.91 (2C, C15, C15'), 24.82 (2C, C16, C16'), 24.78 (2C, C10, C10'), 23.37 (2C, C17, C17'), 23.28 (2C, C12, C12').

6 NMR studies of cyclopentene formation from the Michael addition products

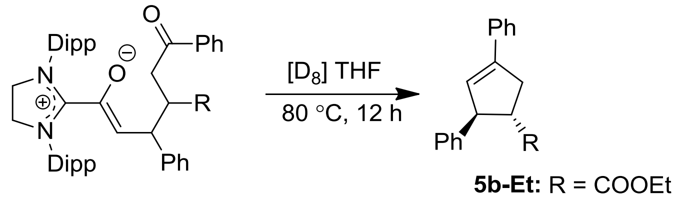

4b-Et, 4c

5b-Et: R = COOEt
5c: R = Ph

The Michael addition products **4b-Et** and **4c** were prepared in an NMR tube according to the previously described procedure (see page S8, S9). These NMR tubes were heated in an oil bath to 80 °C, and the course of the reaction was followed by NMR spectroscopy. The formation of the cyclopentenes **5b-Et** (see figure S1-S2 for characteristic signals of **5b-Et**) and **5c** (see figure S3-S4 for characteristic signals of **5c**) were observed.

The identity of the **5b-Et** and **5c** was proven by independent synthesis (see page S19-S20).

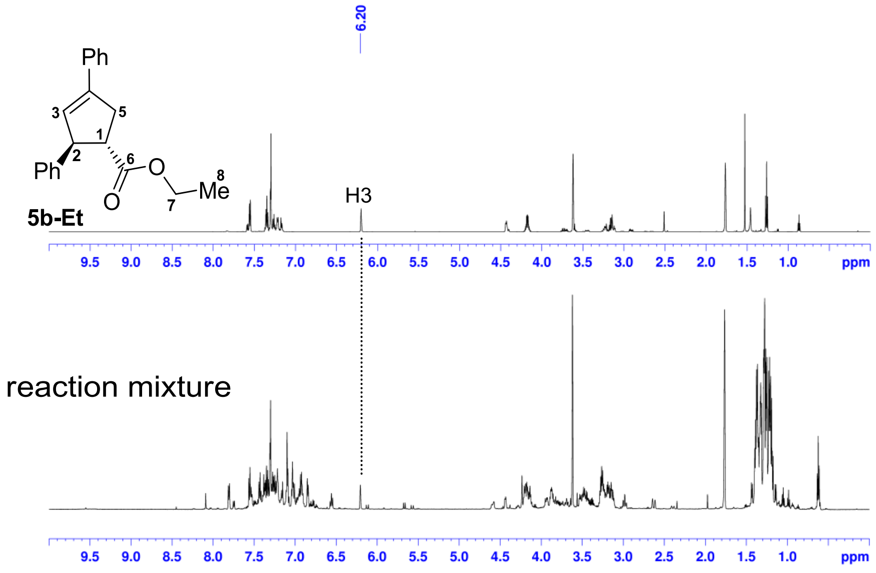

Figure S1. Top: ^{1}H NMR spectrum of **5b-Et** (trans:cis 4.3:1) ([D$_8$]THF, 150 MHz, 298 K). Bottom: ^{1}H NMR spectrum ([D$_8$]THF, 600 MHz, 298 K) resulting from the transformation of the Michael addition product **4b-Et** upon heating to 80 °C for 12 h, formation of **5b-Et**.

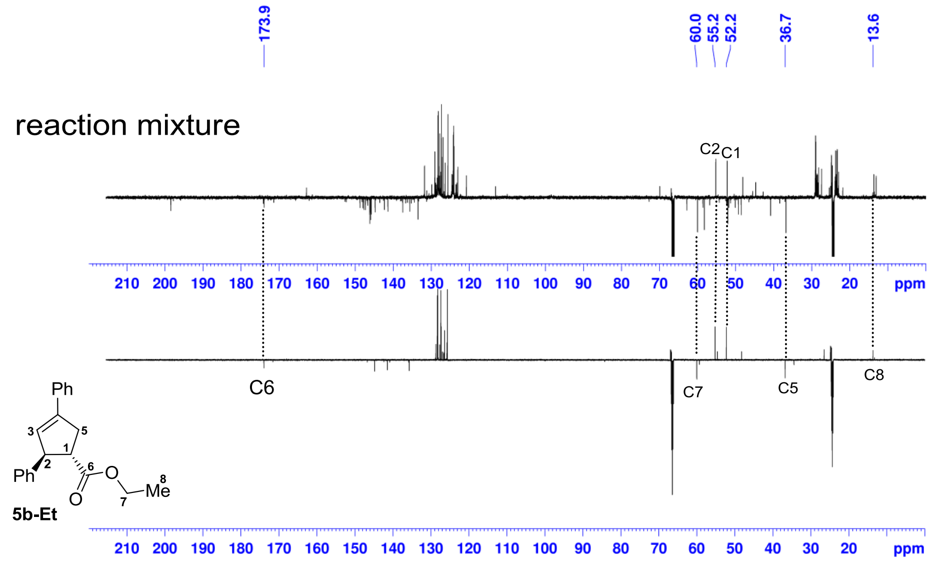

Figure S2. Top: ^{13}C NMR spectrum ([D$_8$]THF, 600 MHz, 298 K) resulting from the transformation of the Michael addition product **4b-Et** upon heating to 80 °C for 12 h, formation of **5b-Et**. Bottom: ^{13}C DEPTQ NMR spectrum of **5b-Et** (trans:cis 4.3:1) ([D$_8$]THF, 150 MHz, 298 K).

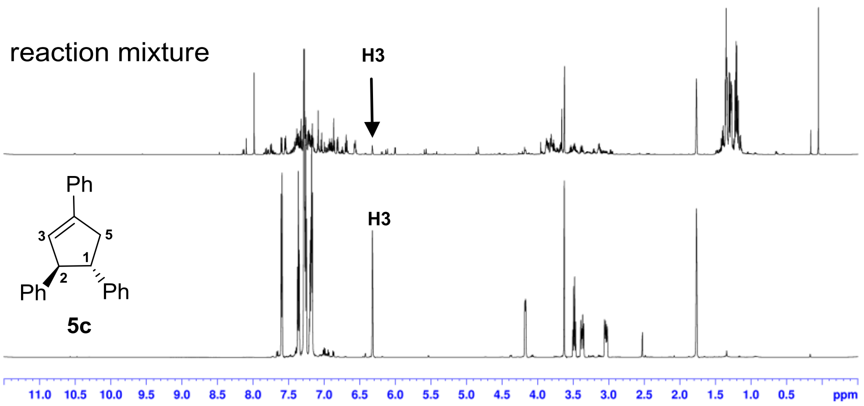

Figure S3. Top: ^{1}H NMR spectrum ([D$_8$]THF, 600 MHz, 298 K) resulting from the transformation of the Michael addition product **4c** upon heating to 80 °C for 12 h, formation of **5c**. Bottom: ^{1}H NMR spectrum of **5c** ([D$_8$]THF, 150 MHz, 298 K).

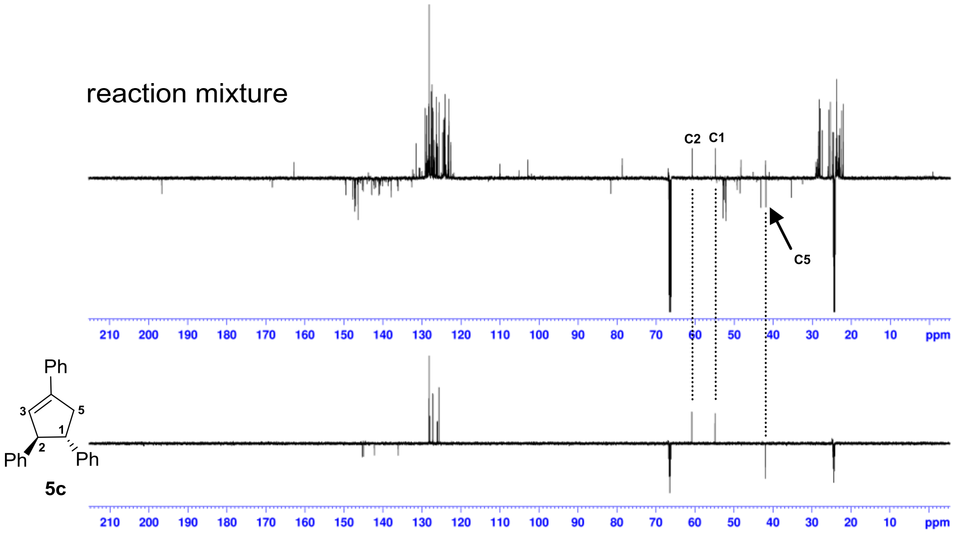

Figure S4. Top: ^{13}C NMR spectrum ([D$_8$]THF, 600 MHz, 298 K) resulting from the transformation of the Michael addition product **4c** uponr heating to 80 °C for 12 h, formation of **5c**. Bottom: ^{13}C DEPTQ NMR spectrum of **5c** ([D$_8$]THF, 150 MHz, 298 K).

7 NMR studies of γ-butyrolactone formation from pre-formed 2,2-diamino dienol

7.1 Reaction of the 2,2-diamino dienol 1 with an equimolar amount of benzaldehyde (6)

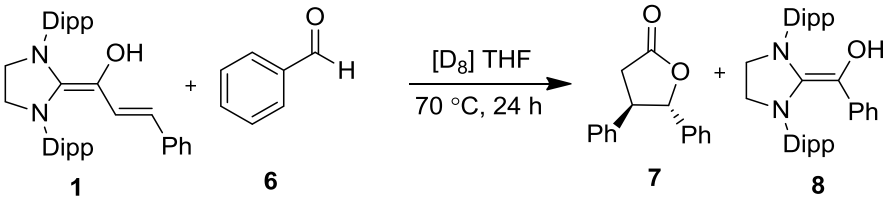

In a glovebox, an NMR tube was charged with 15 mg (38 µmol, 1.0 equiv) of SiPr in [D$_8$] THF and sealed with a septum. 1.0 Equiv of *E*-cinnamic aldehyde (5.1 mg, 4.8 µl) was added with a syringe followed by measuring ^{1}H NMR, showing the signals of **1** (see figure S39). 1.0 Equiv of benzaldehyde (4.1 mg, 3.9 µl) was added and this NMR tube was heated in an oil bath to 70 °C, and the course of the reaction was followed by NMR spectroscopy. The formation of **7** was observed (see figure S5-S6 for characteristic signals of **7**) along with the regenerated SIPr, which then reacts with benzaldehyde affording the diamino enol **8** (OH is indicative peak in the case of diamino enol **8**).

The identity of the **7** was proven by independent synthesis (page S20).

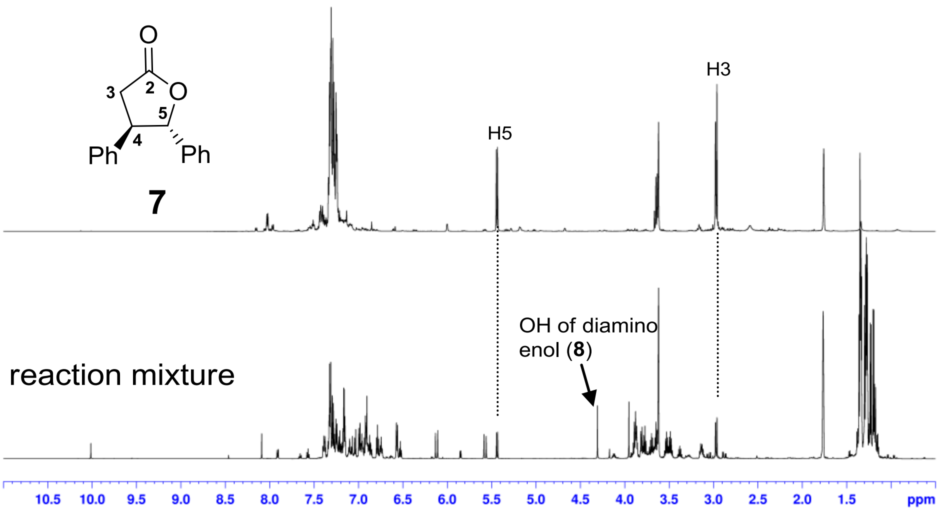

Figure S5. Top: ^{1}H NMR spectrum of **7** ([D$_8$]THF, 150 MHz, 298 K). Bottom: ^{1}H NMR spectrum (recorded after 24 h in [D$_8$]THF, 150 MHz, 298 K) resulting from the reaction of the 2,2-diamino dienol **1** with benzaldehyde (1 equiv) at 70 °C, formation of **7** and diaminoenol (**8**).

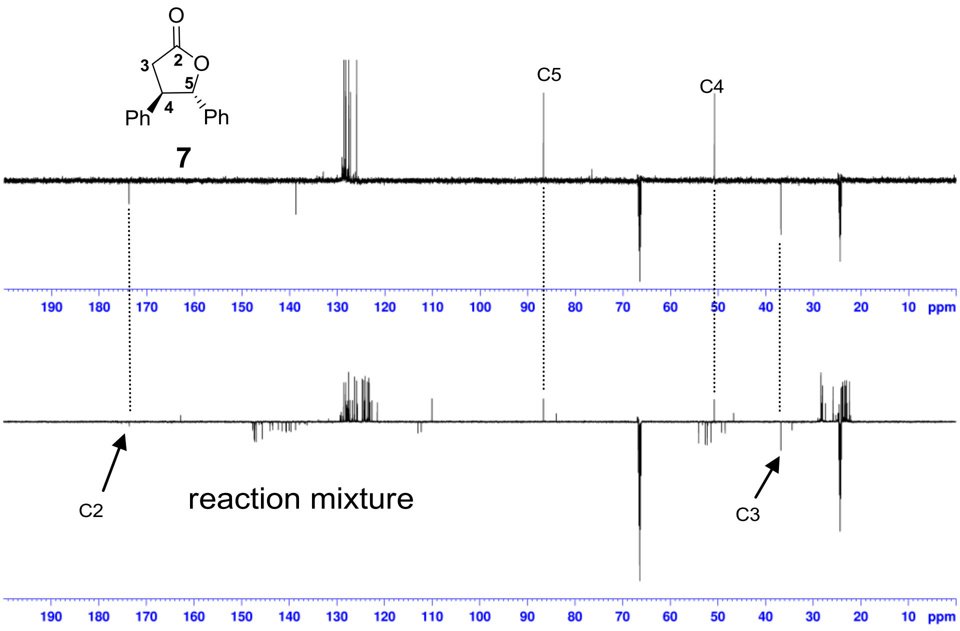

Figure S6. Top: ^{13}C DEPTQ NMR spectrum of **7** ([D$_8$]THF, 150 MHz, 298 K). Bottom: ^{13}C DEPTQ NMR spectrum (recorded after 24 h in [D$_8$]THF, 150 MHz, 298 K) resulting from the reaction of the 2,2-diamino dienol **1** with benzaldehyde (1 equiv) at 70 °C, formation of **7.**

8 NMR studies of γ,δ-unsaturated δ-lactone formation from pre-formed azolium enolates

8.1 Reaction of the azolium enolate 11a with an equimolar amount of *E*-chalcone (3c)

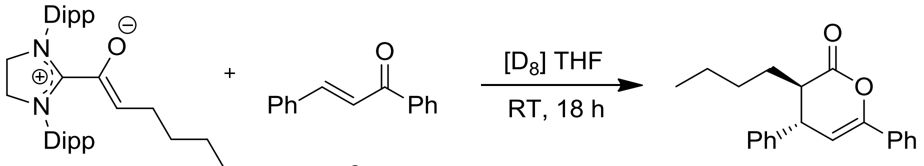

11a **3c** **12a**

In a glovebox, an NMR tube was charged with 15 mg (38 µmol, 1.0 equiv) of SiPr in [D_8] THF and sealed with a septum. 1.0 Equiv of *E*-2-hexenal (3.8 mg, 4.5 µl) was added with a syringe followed by measuring ^{1}H NMR, showing the signals of the azolium enolate **11a**.
E-chalcone (8.0 mg, 38 µmol, 1.0 eq.) was added and the course of the reaction was followed by NMR spectroscopy. The formation of 1**2a** (see figure S7-S11 for characteristic signals of **12a**) was observed.

The identity of the 1**2a** was proven by independent synthesis (see page S21).

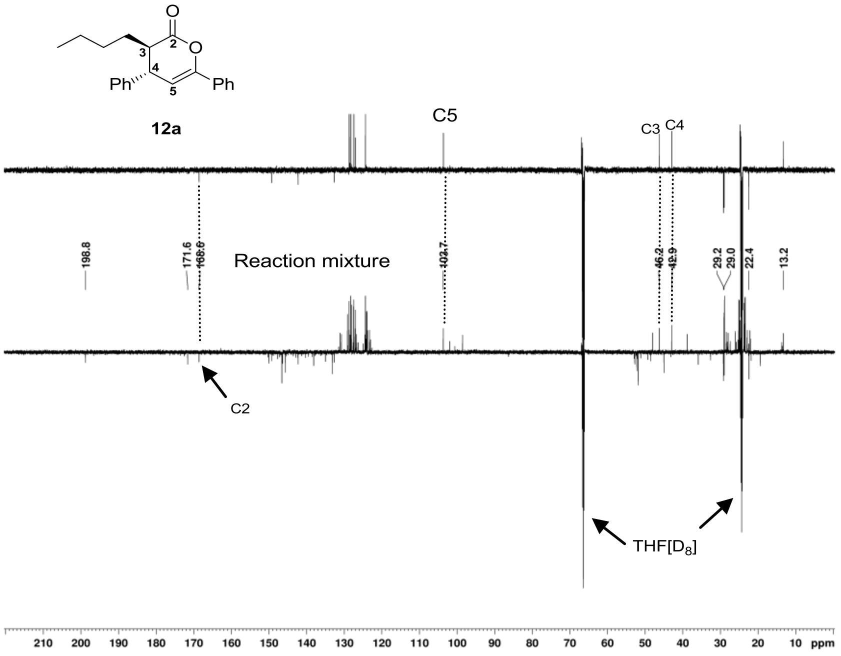

Figure S7. Top: ^{13}C DEPTQ NMR spectrum of **12a** ([D_8]THF, 150 MHz, 298 K). Bottom: ^{13}C DEPTQ NMR spectrum (recorded after 18 h in [D_8]THF, 150 MHz, 298 K) resulting from the reaction of the azolium enolate **11a** with *E*-chalcone (1 equiv), formation of **12a**.

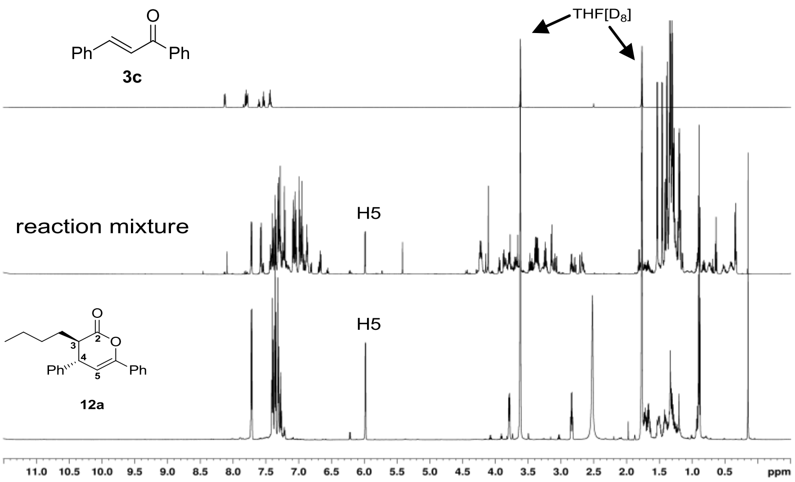

Figure S8. Top: ^{1}H NMR spectrum of *E*-chalcone ([D$_8$]THF, 600 MHz, 298 K). Middle: ^{1}H NMR spectrum (recorded after 18 h in [D$_8$]THF, 600 MHz, 298 K) resulting from the reaction of the azolium enolate **11a** with *E*-chalcone (1 equiv), formation of **12a.** Bottom: ^{1}H NMR spectrum of **12a** ([D$_8$]THF, 600 MHz, 298 K).

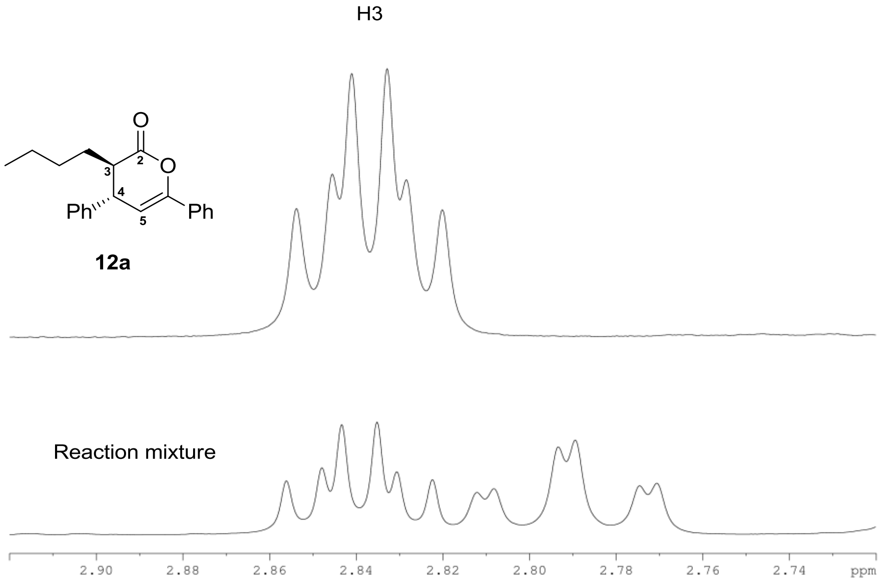

Figure S9. Top: Part of ^{1}H NMR spectrum of **12a** ([D$_8$]THF, 600 MHz, 298 K) Bottom: Part of ^{1}H NMR spectrum (recorded after 18 h in [D$_8$]THF, 600 MHz, 298 K) resulting from the reaction of the azolium enolate **11a** with *E*-chalcone (1 equiv), formation of **12a**.

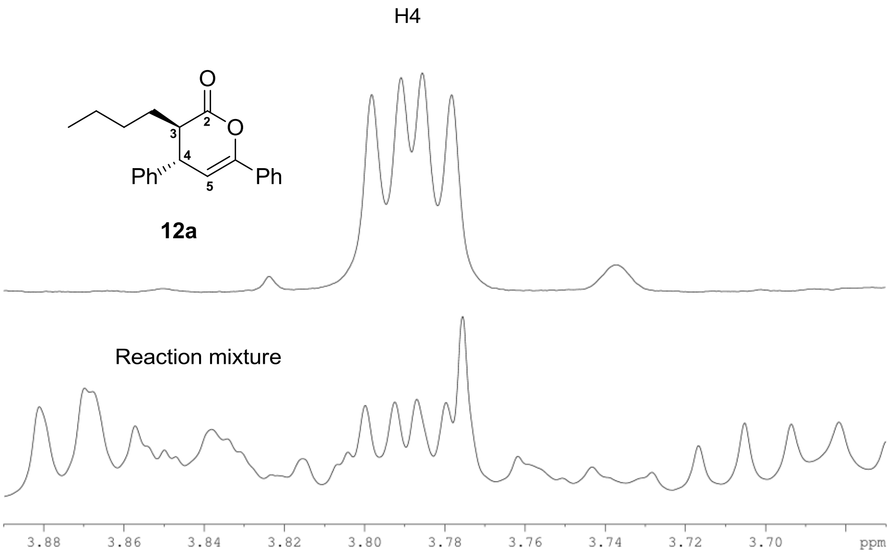

Figure S10. Top: Part of ^{1}H NMR spectrum of **12a** ([D$_8$]THF, 600 MHz, 298 K). Bottom: Part of ^{1}H NMR spectrum (recorded after 18 h in [D$_8$]THF, 600 MHz, 298 K) resulting from the reaction of the azolium enolate **11a** with *E*-chalcone (1 equiv), formation of **12a**.

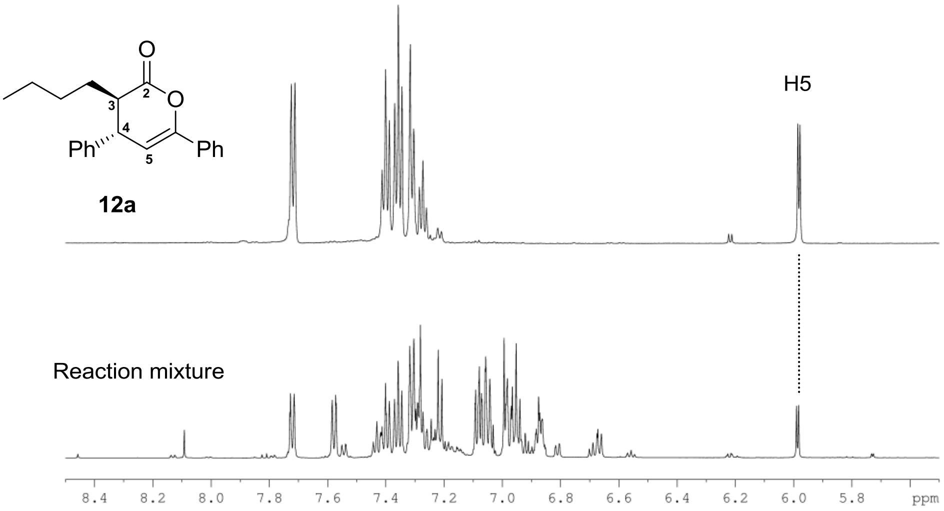

Figure S11. Top: Part of ^{1}H NMR spectrum of **12a** ([D$_8$]THF, 600 MHz, 298 K). Bottom: Part of ^{1}H NMR spectrum (recorded after 18 h in [D$_8$]THF, 600 MHz, 298 K) resulting from the reaction of azolium enolate **11a** with *E*-chalcone (1 equiv), formation of **12a**.

8.2 Reaction of the azolium enolate 11b with *E*-chalcone (3c)

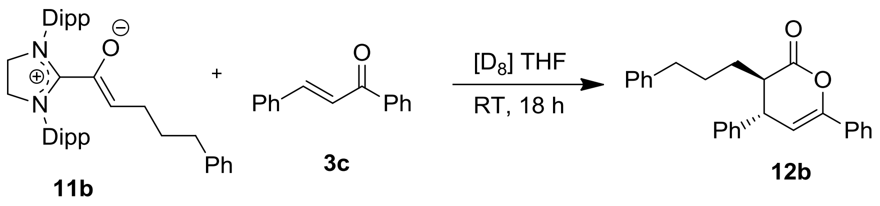

In a glovebox, an NMR tube was charged with 22 mg (56 µmol, 1.0 equiv) of SiPr in [D$_8$] THF and sealed with a septum. 1.0 Equiv. of (2*E*)-5-phenylpent-2-enal (9.0 mg, 56 µmol) was added, followed by measuring ^{1}H NMR, showing the signals of the azolium enolate **11b**. *E*-chalcone (1.8 eq, 21 mg) was added and the course of the reaction was followed by NMR spectroscopy. The formation of 1**2b** (see figure S12-S13 for characteristic signals of **12b**) was observed.

The identity of the 1**2b** was proven by independent synthesis (see page S22).

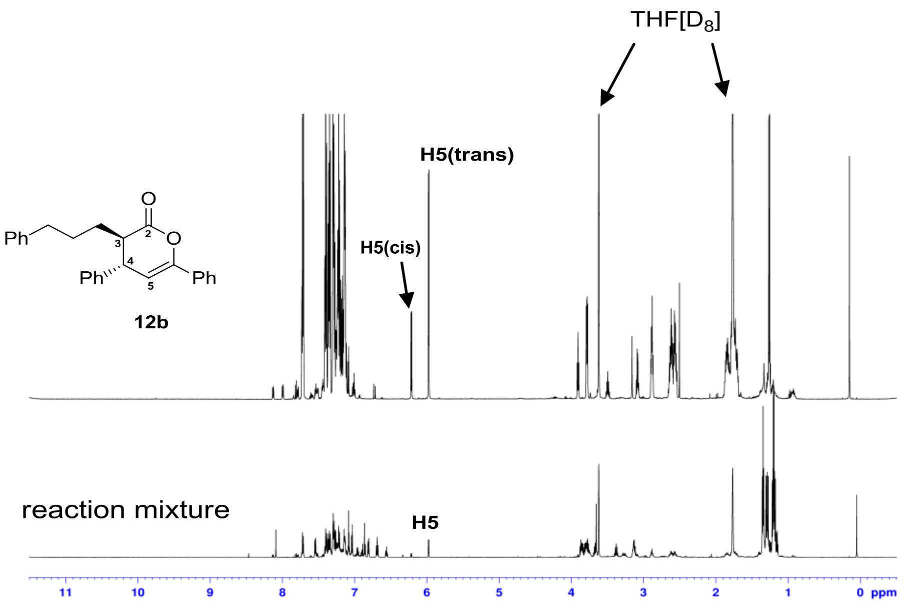

Figure S12. Top: ^{1}H NMR spectrum of **12b** (trans:cis 2.8:1) ([D$_8$]THF, 600 MHz, 298 K). Bottom: ^{1}H NMR spectrum (recorded after 18 h in [D$_8$]THF, 600 MHz, 298 K) resulting from the reaction of the azolium enolate **11b** with *E*-chalcone, formation of the **12b**.

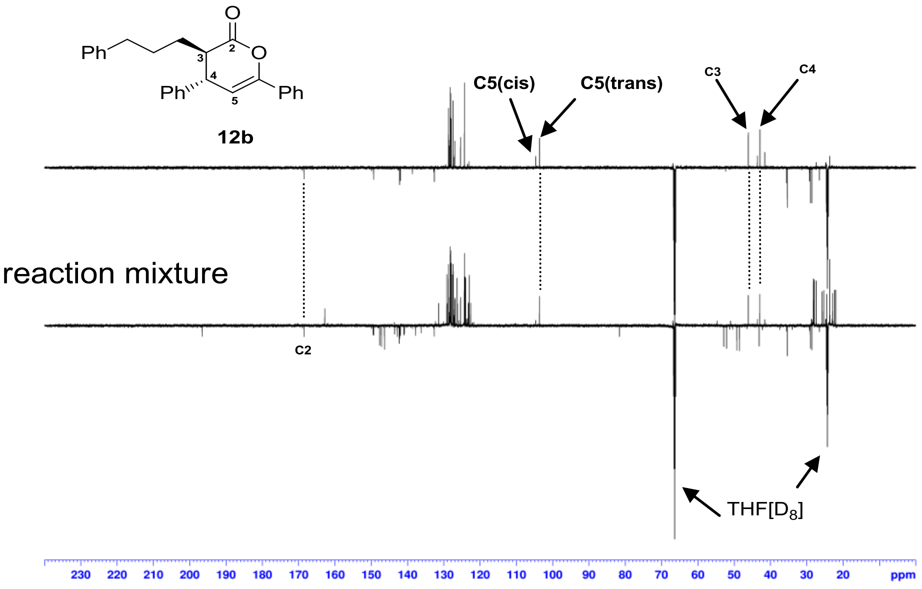

Figure S13. Top: ^{13}C NMR spectrum of **12b** (trans:cis 2.8:1) ([D$_8$]THF, 150 MHz, 298 K). Bottom: ^{13}C NMR spectrum (recorded after 18 h in [D$_8$]THF, 150 MHz, 298 K) resulting from the reaction of the azolium enolate **11b** with *E*-chalcone, formation of the lactone **12b**.

9 Independent synthesis of cyclopentene products

9.1 Synthesis of *rac*-ethyl (1*R*,2*R*)-2,4-diphenylcyclopent-3-en-1-carboxylate (5b-Et)

The cyclopentene product **5b** was synthesized according to the procedure reported by Nair et al..[4]

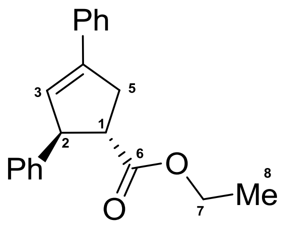

5b-Et

^{1}H NMR (600 MHz, [D$_8$]THF): δ 7.56-7.55 (m, 2H, Ar), 7.36-7.30 (m, 8 H, Ar), 6.20 (br s, 1H, H3), 4.44-4.43 (m, 1H, H2), 4.20-4.15 (m, 2H, H7), 3.24-3.14 (m, 3H, H1, H5), 1.26 (t, 3H, ^3J = 7.1 Hz, H8). **^{13}C NMR (150 MHz, [D$_8$]THF): δ** 173.9 (1C, C6), 144.8, 141.4, 135.6, 128.2, 128.1, 127.31, 127.29, 127.24, 126.3, 125.6, 60.0 (1C, C7), 55.2 (1C, C2), 52.2 (1C, C1), 36.8 (1C, C5), 13.6 (1C, C8). **IR (ATR) $\tilde{\nu}$ (cm^{-1}):** 2964, 1728, 1492, 1446, 1369, 1226, 1172, 1074, 1031, 804, 754, 692. **HR-MS (EI) [M]:** *m/z* calcd for [$C_{20}H_{20}O_2$] [M] 292.1463 found 292.147.

9.2 Synthesis of *rac*-1,1',1''-[(*1R*,*2R*)-cyclopent-3-ene-1,2,4-triyl]tribenzene (5c)

The cyclopentene product **5c** was synthesized according to the procedure reported by Nair et al.,[4] analytical data are consistent with published ones.[4]

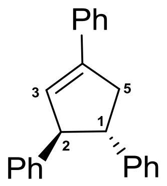

5c

^{1}H NMR (600 MHz, [D$_8$]THF): δ 7.60-7.59 (m, 2H, Ar), 7.37-7.35 (m, 2H, Ar), 7.29-7.25 (m, 7H, Ar), 7.21-7.16 (m, 4H, Ar) 6.32 (br s, 1H, H3), 4.17-4.16 (m,1H), 3.50-3.46 (m, 1H), 3.39-3.35 (m, 1H), 3.06-3.02 (m, 1H). **^{13}C NMR (150 MHz, [D$_8$]THF): δ** 145.3, 145.0, 142.2, 136.1, 128.2, 128.0, 127.3, 127.2, 127.1, 126.1, 126.0, 125.6, 60.8 (C2), 54.8 (C1), 41.9 (C5). **IR (ATR) $\tilde{\nu}$ (cm^{-1}):** 3024, 2916, 1598, 1492, 1450, 1261, 1234, 1159, 1076, 1026, 869, 754. **HR-MS (EI) [M]:** *m/z* calcd for [$C_{23}H_{20}$] [M] 296.1565 found 296.156.

10 Independent synthesis of *rac*-(4*R*,5*S*)-4,5-diphenyloxolan-2-one (7)

The lactone product **7** was synthesized according to the known literature protocol,[5] analytical data are consistent with published ones.[6]

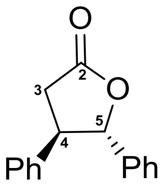

7

^{1}H NMR (600 MHz, [D$_8$]THF): δ 7.34-7.24 (m, Ar, 10H), 5.44 (d, 1H, ^{3}J = 9.0 Hz, H5), 3.67-3.62 (m, 1H, H4), 2.98-2.96 (m, 2H, H3). **^{13}C NMR (150 MHz, [D$_8$]THF): δ** 173.73 (C2), 138.66, 138.64, 128.61, 128.22, 128.15, 127.58, 127.25, 125.90, 86.68 (C5), 50.76 (C4), 36.82 (C3). **IR (ATR) (cm^{-1}):** 3030, 2962, 1782, 1494, 1454, 1269, 1197, 1139, 991, 977, 879, 759, 694. **HR-MS (EI) [M]:** *m/z* calcd for [$C_{16}H_{14}O_2$] [M] 238.0994 found 238.099.

11 Isolation of γ,δ-unsaturated δ-lactone products

11.1 Synthesis of *rac*-(*3R,4S*)-3-butyl-4,6-diphenyl-3,4-dihydro-2*H*-pyran-2-one (12a)

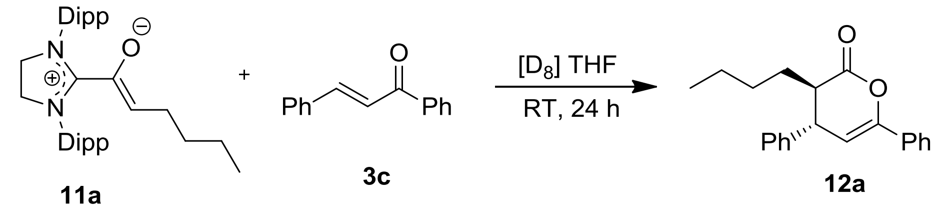

11a **3c** **12a**

In a glovebox, an NMR tube was charged with 15 mg (38 µmol, 1.0 equiv) of SiPr in [D_8] THF and sealed with a septum. 1.0 Equiv of *E*-2-hexenal (3.8 mg, 4.5 µl) was added with a syringe followed by measuring ^{1}H NMR, showing the signals of the azolium enolate **11a**. *E*-chalcone (8.0 mg, 1.0 eq.) was added and the reaction was followed by NMR. After 24 h the reaction mixture was subjected to column chromatography on silica gel, eluting with 1:20 EtOAc: *n*-hexane, affording **12a** (5 mg, 42%, trans:cis:13.5:1) (see figure S87-S95 for 1D and 2D NMR).

The trans-configuration of **12a** was assigned by NOE spectroscopy (strong NOE between H4 and H7) (see figure S93 for NOE spectrum).

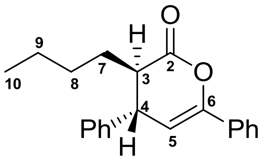

12a

^{1}H NMR (600 MHz, CD_2Cl_2): δ 7.59-7.58 (m, 2H, Ar), 7.33-7.25 (m, 5H, Ar), 7.20-7.15 (m, 3H, Ar), 5.79 (d, 1H, ^3J = 4.4 Hz, H5), 3.63 (dd, 1H, $^3J_{H4\text{-}H5}$ = 4.4 Hz, $^3J_{H4\text{-}H3}$ = 7.6 Hz, H4), 2.68 (m, 1H, H3), 1.67-1.54 (m, 2H, H7), 1.38-1.30 (m, 2H, H8), 1.23-1.15 (m, 2H, H9), 0.78 (t, 3H, ^3J = 7.2Hz, H10). **^{13}C NMR (150 MHz, CD_2Cl_2): δ** 169.9 (1C, C2), 149.6 (1C, C6), 141.7 (1C, Ar), 132.4 (1C, Ar), 129.1 (1C, Ar), 129.05 (2C, Ar) 128.5 (2C, Ar), 127.4 (2C, Ar), 127.34 (1C, Ar), 124.6 (2C, Ar), 103.4 (1C, C5), 46.9 (1C, C3), 42.9 (1C, C4), 29.6 (1C, C7), 29.0 (1C, C8), 22.5 (1C, C9), 13.6 (1C, C10). **HR-MS (EI)** *m/z* calcd for [$C_{21}H_{22}O_2$] [M] 306.1620 found 306.163.

11.2 Synthesis of *rac*-(3*R*,4*S*)-4,6-diphenyl-3-(3-phenylpropyl)-3,4-dihydro-2*H*-pyran-2-one (12b)

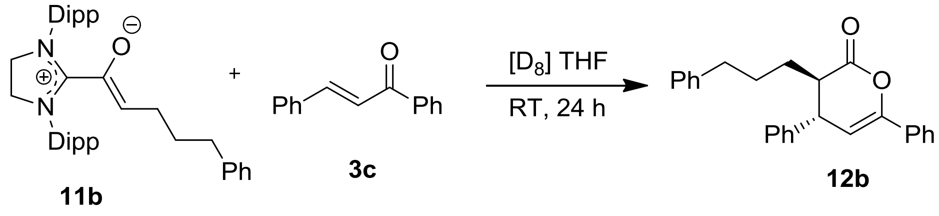

In a glovebox, an NMR tube was charged with 22 mg (56 µmol, 1.0 equiv) of SiPr in [D_8] THF and sealed with a septum. 1.0 Equiv. of *E*-5-phenylpent-2-enal (9.0 mg) was added followed by measuring ^{1}H NMR, showing the signals azolium enolate **11b**. *E*-chalcone (1.8 eq, 21 mg) was added and the reaction was followed by NMR. After 24 h the reaction mixture was subjected to column chromatography on silica gel, eluting with 1:10 EtOAc: *n*-hexane, affording the **12b** (6 mg, 29%, trans:cis:2.8:1) (see figure S96-S97 for 1D NMR).

12 NMR spectra

12.1 ^{1}H and ^{13}C NMR spectra of *E*-5-phenylpent-2-enal (9b)

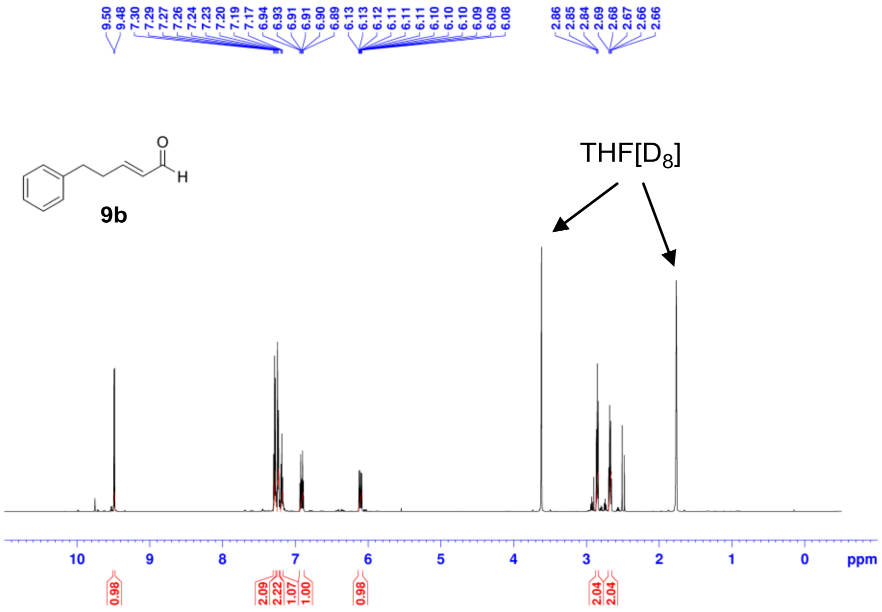

Figure S14. ^{1}H(600 MHz) NMR spectrum of **9b** ([D$_8$]THF, 298 K).

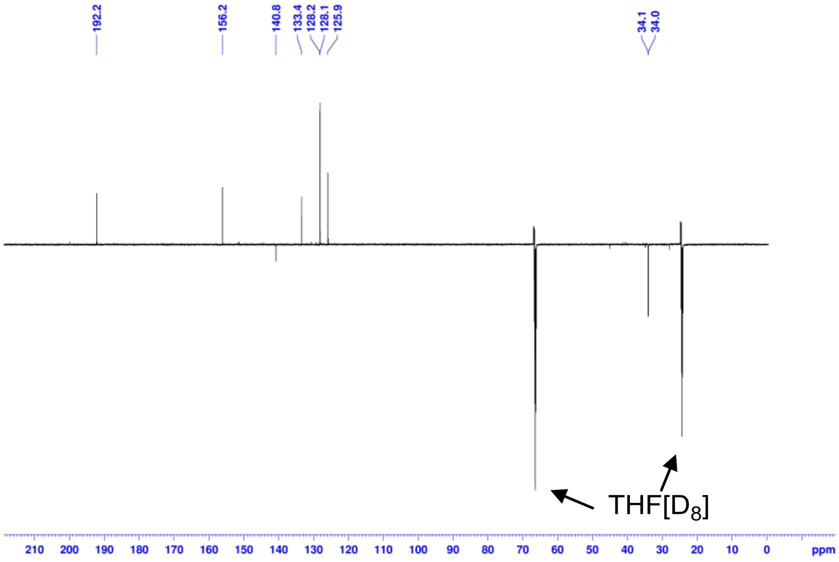

Figure S15. ^{13}C(150 MHz) DEPTQ NMR spectrum of **9b** ([D$_8$]THF, 298 K).

12.2 ^{1}H and ^{13}C NMR spectra of methyl *E*-3-benzoylacrylate (3b-Me)

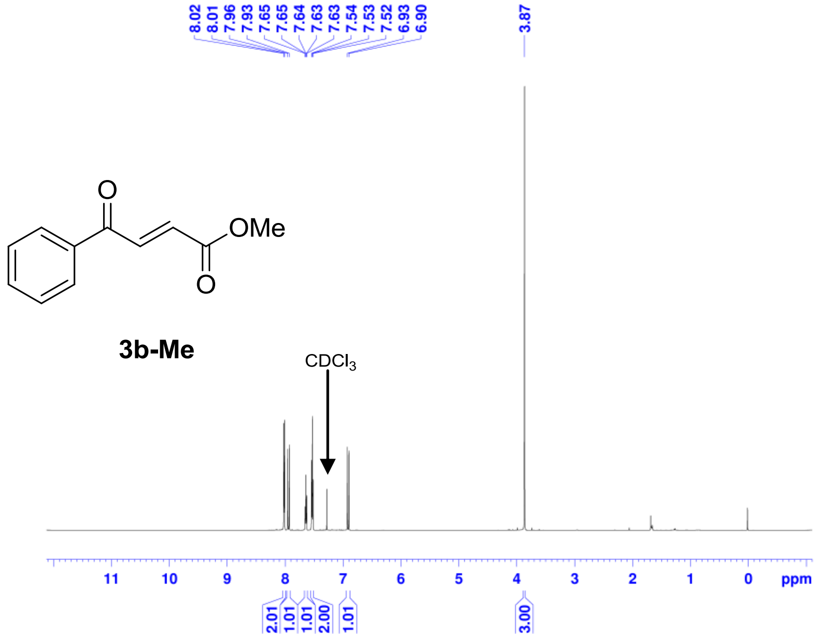

Figure S16. ^{1}H(600 MHz) NMR spectrum of **3b-Me** ($CDCl_3$, 298 K).

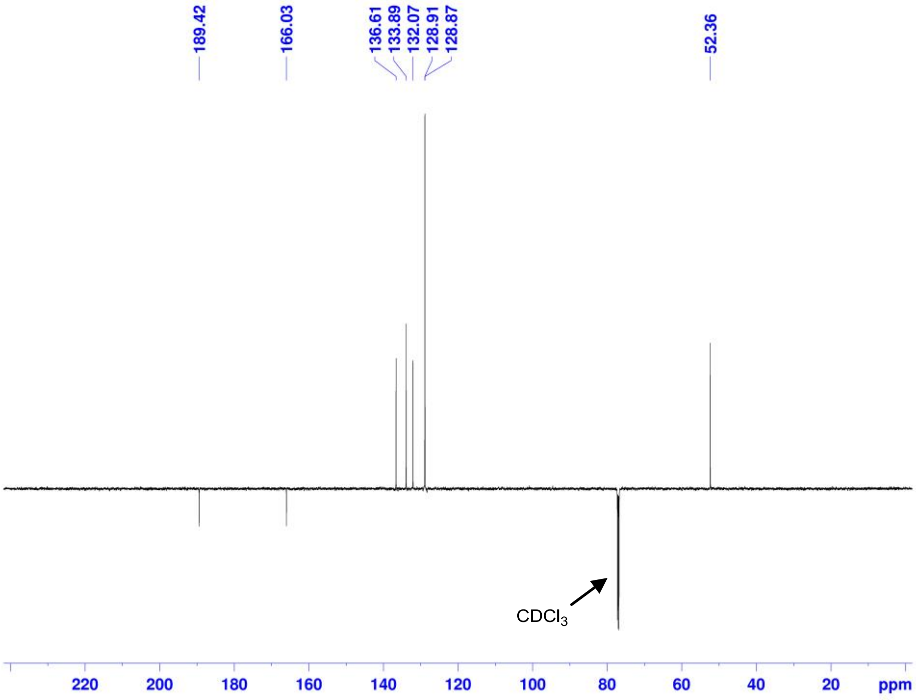

Figure S17. ^{13}C(150 MHz) DEPTQ NMR spectrum of **3b-Me** ($CDCl_3$, 298 K).

12.3 1D and 2D NMR spectra of the azolium enolate 11a

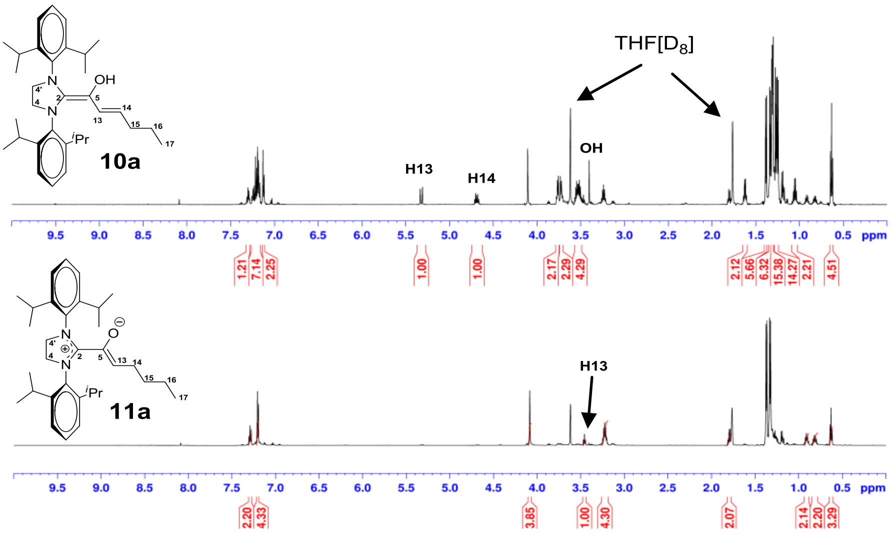

Figure S18. Top: ¹H NMR spectrum of diamino dienol **10a** (containing minor amout of azolium enolate **11a**) Bottom: ¹H NMR spectrum of azolium enolate **11a** (600 MHz, [D$_8$]THF at 298 K)

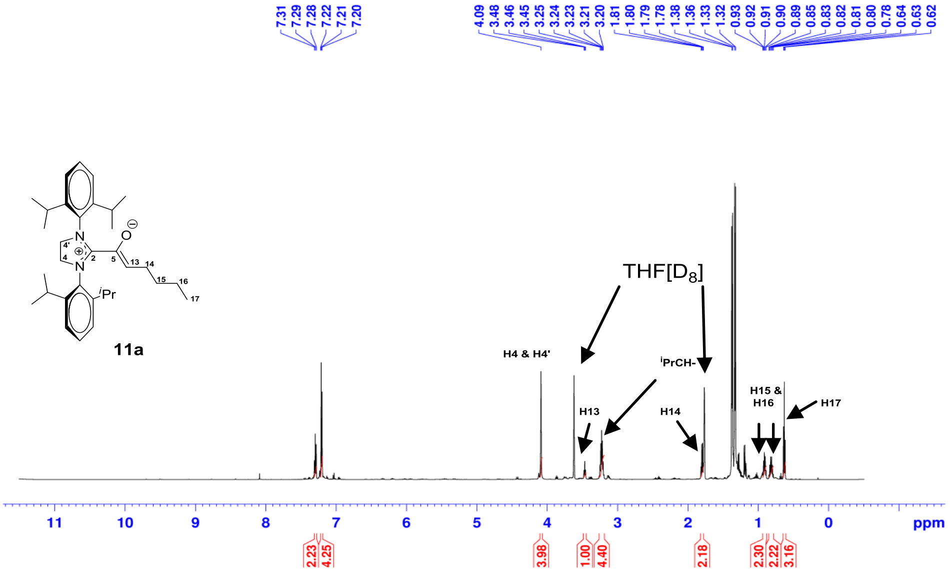

Figure S19.¹H(600 MHz) NMR spectrum of **11a** ([D$_8$]THF, 298 K).

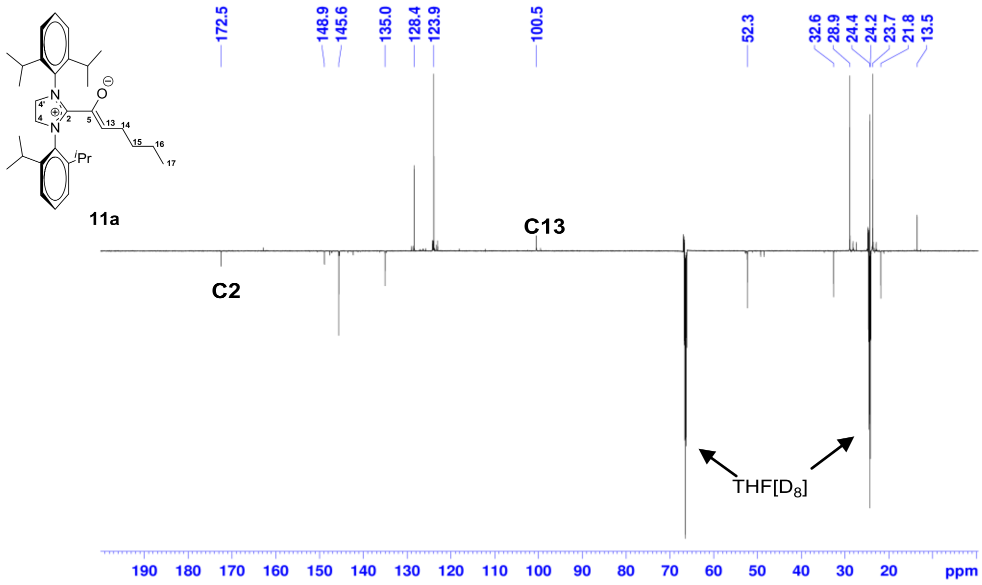

Figure S20. ^{13}C(150 MHz) DEPTQ NMR spectrum of **11a** ([D$_8$]THF, 298 K).

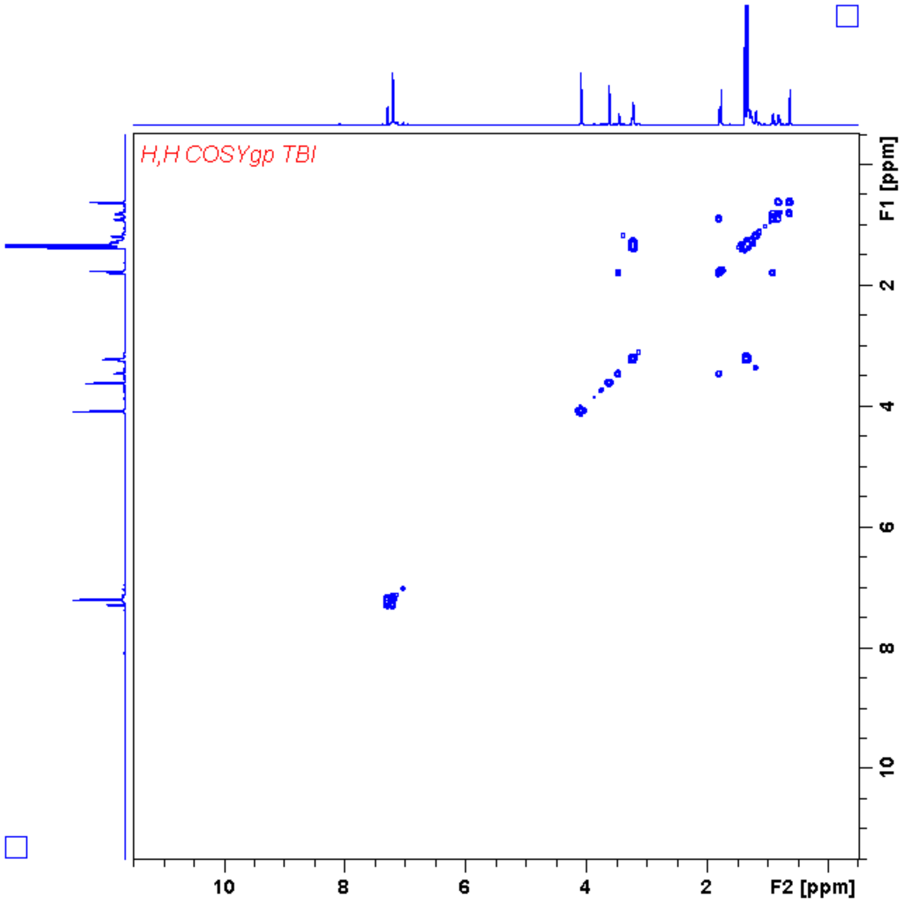

Figure S21. ^{1}H,^{1}H(600MHz) COSY NMR spectrum of **11a** ([D$_8$]THF, 298 K).

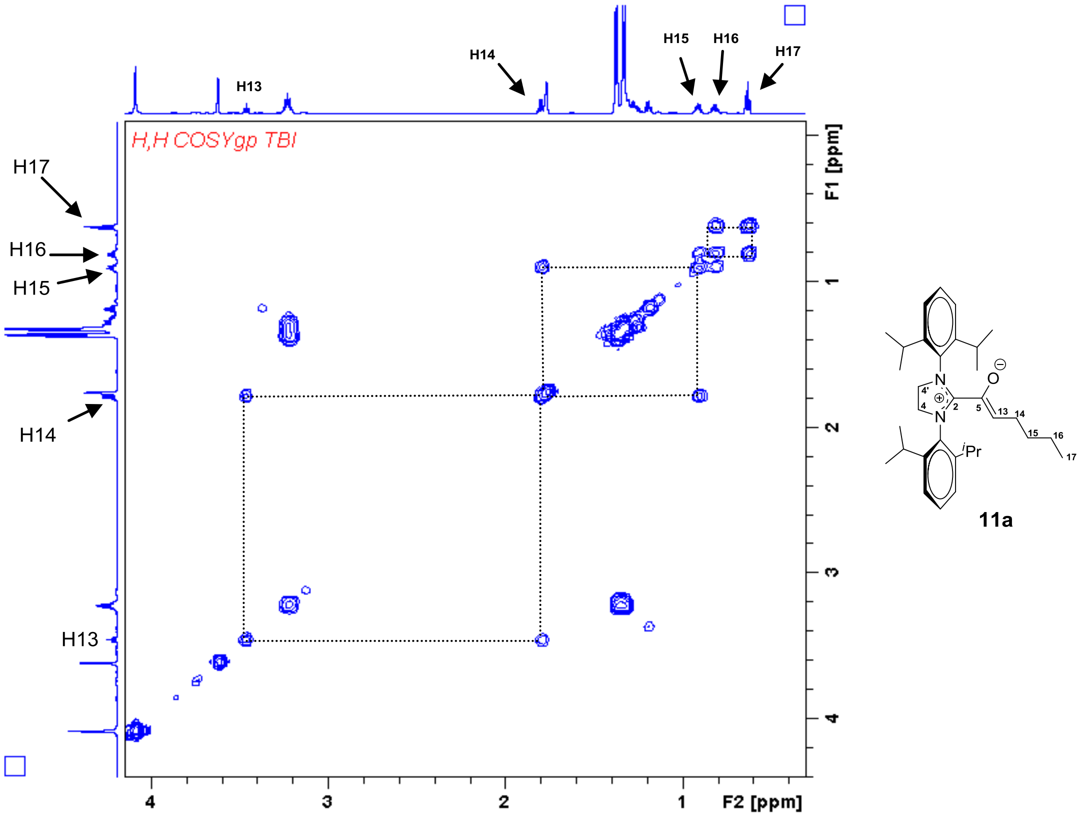

Figure S22. Part of the $^{1}H,^{1}H$(600MHz) COSY NMR spectrum of **11a** ([D_8]THF, 298 K).

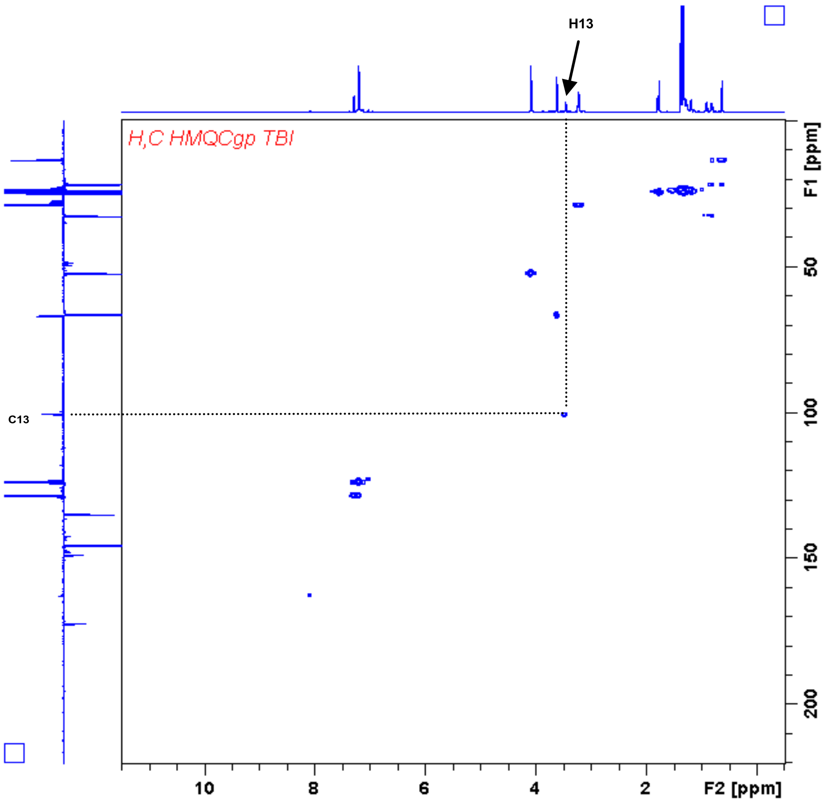

Figure S23. ^{1}H(600 MHz),^{13}C(150 MHz) HMQC NMR spectrum of **11a** ([D_8]THF, 298 K).

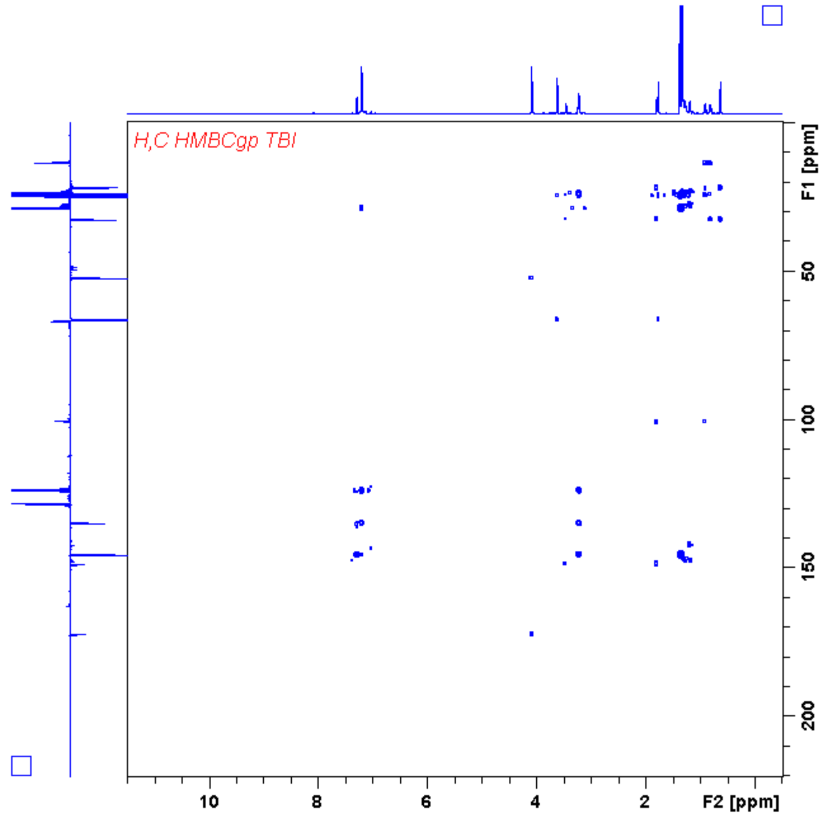

Figure S24. ^{1}H(600 MHz),^{13}C(150 MHz) HMBC NMR spectrum of **11a** ([D_8]THF, 298 K).

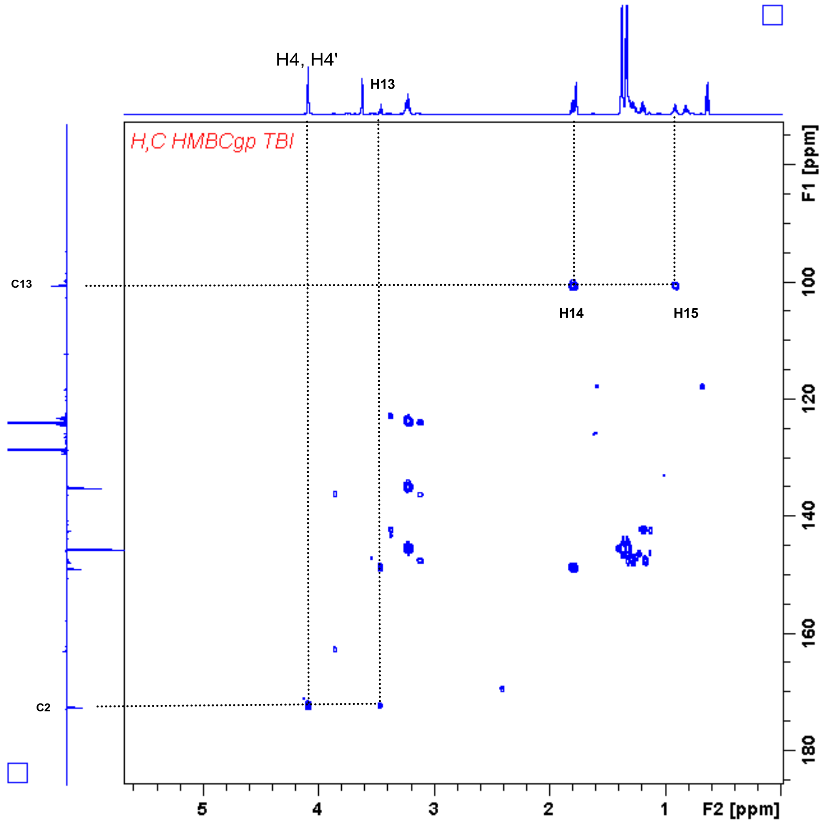

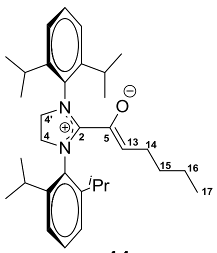

11a

Figure S25. Part of the ^{1}H(600 MHz),^{13}C(150 MHz) HMBC NMR spectrum of **11a** ([D_8]THF, 298 K).

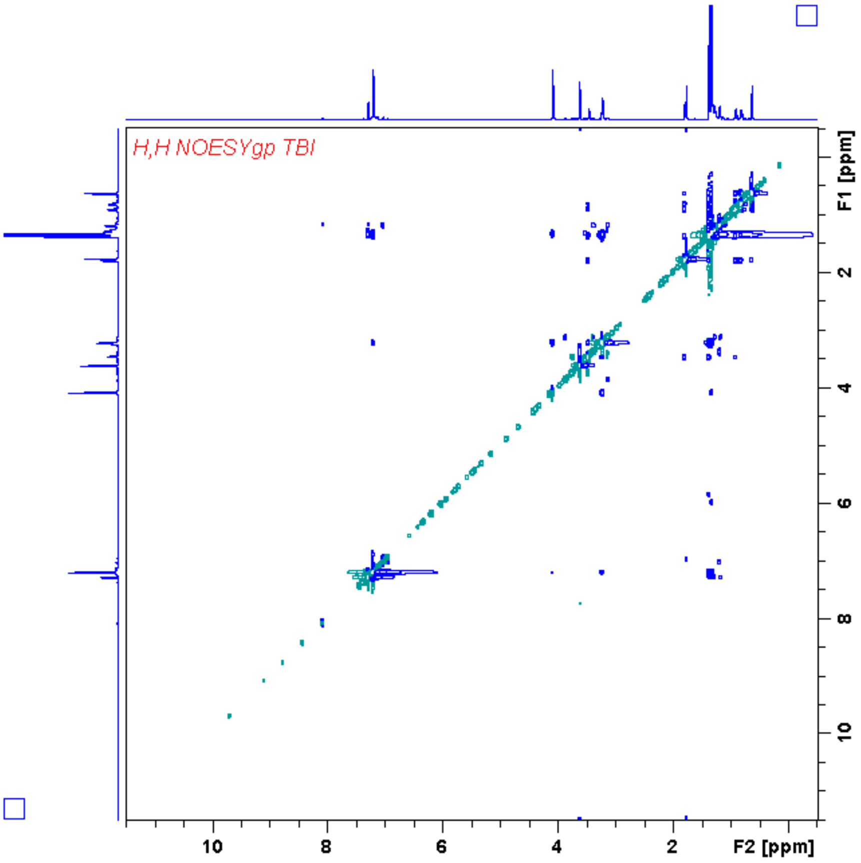

Figure S26. $^{1}H,^{1}H$ NOESY NMR spectrum of **11a** ([D8]THF, 600 MHz, 298 K, mixing time = 600 ms).

12.4 ^{1}H and ^{13}C NMR spectra of the 2,2-diamino dienol 10b

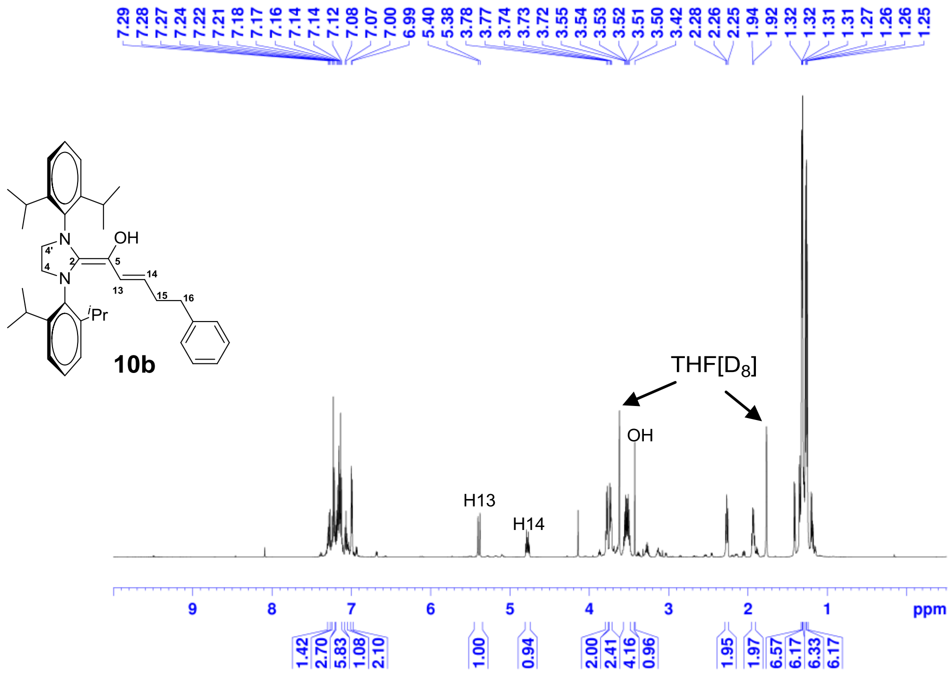

Figure S27. ^{1}H(600 MHz) NMR spectrum of **10b** (containing a minor amount of the azolium enolate **11b**) ([D8]THF, 600 MHz, 298 K).

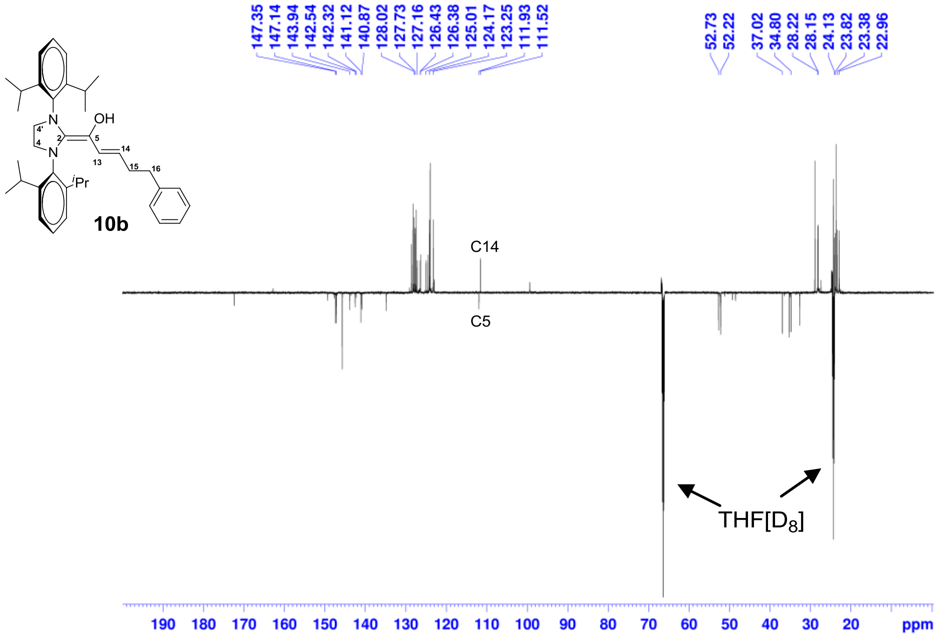

Figure S28. ^{13}C(150 MHz) DEPTQ NMR spectrum of **10b** (containing a minor amount of the azolium enolate **11b**) ([D8]THF, 600 MHz, 298 K).

12.5 1D and 2D NMR spectra of the azolium enolate 11b

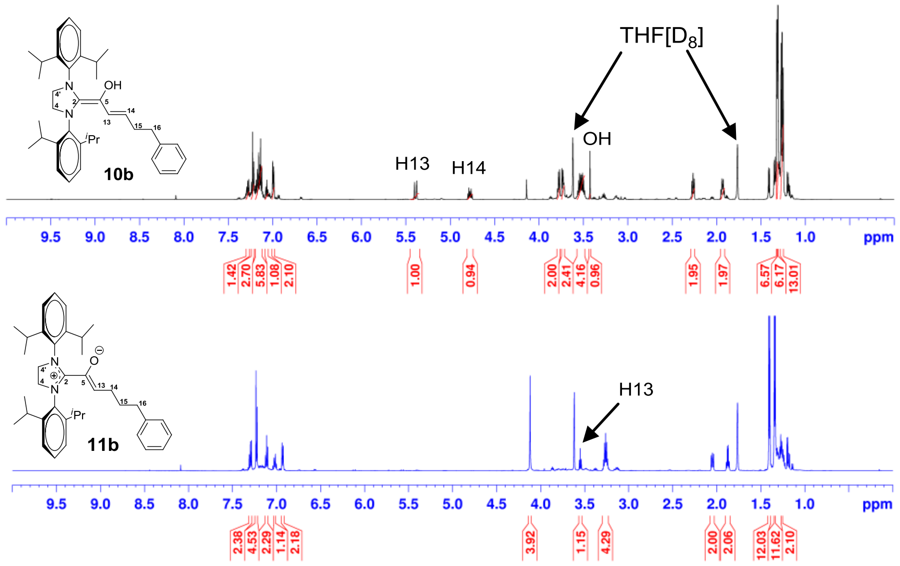

Figure S29. Top: ^{1}H NMR spectrum of diamino dienol **10b** (containing a minor amout of the azolium enolate **11b**) Bottom: ^{1}H NMR spectrum of azolium enolate **11b** (600 MHz, [D$_8$]THF at 298 K).

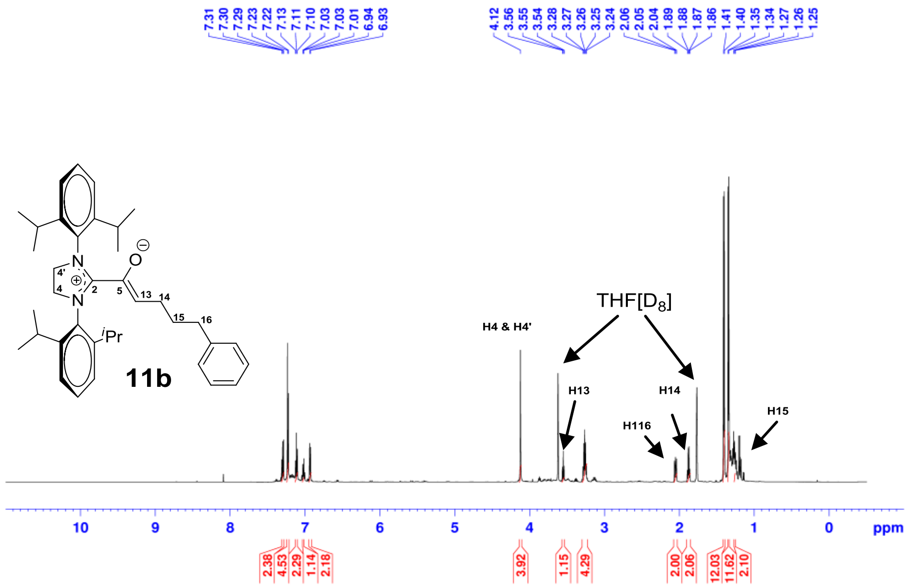

Figure S30. ^{1}H(600 MHz) NMR spectrum of **11b** ([D$_8$]THF, 298 K).

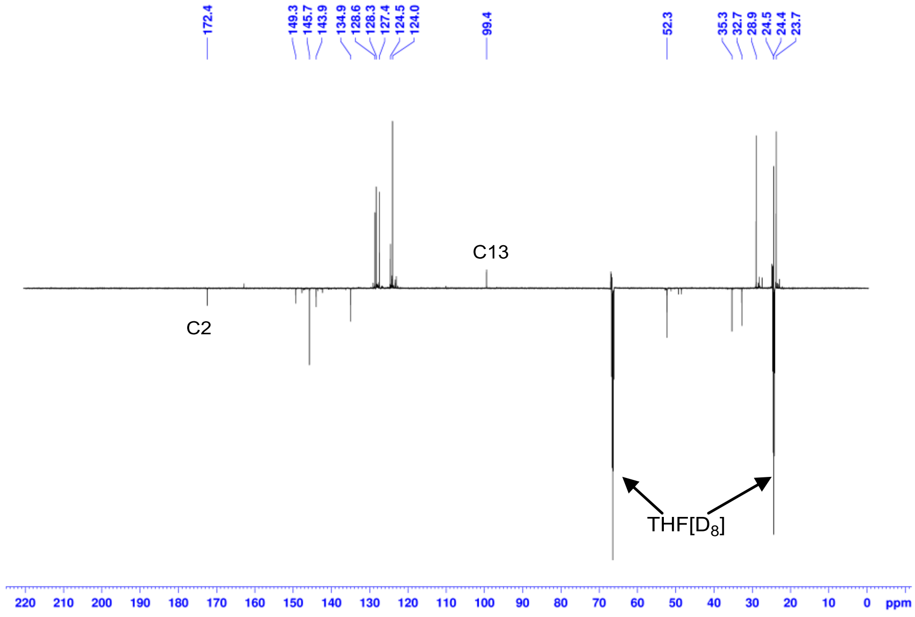

Figure S31. ^{13}C(150 MHz) DEPTQ NMR spectrum of **11b** ([D$_8$]THF, 298 K).

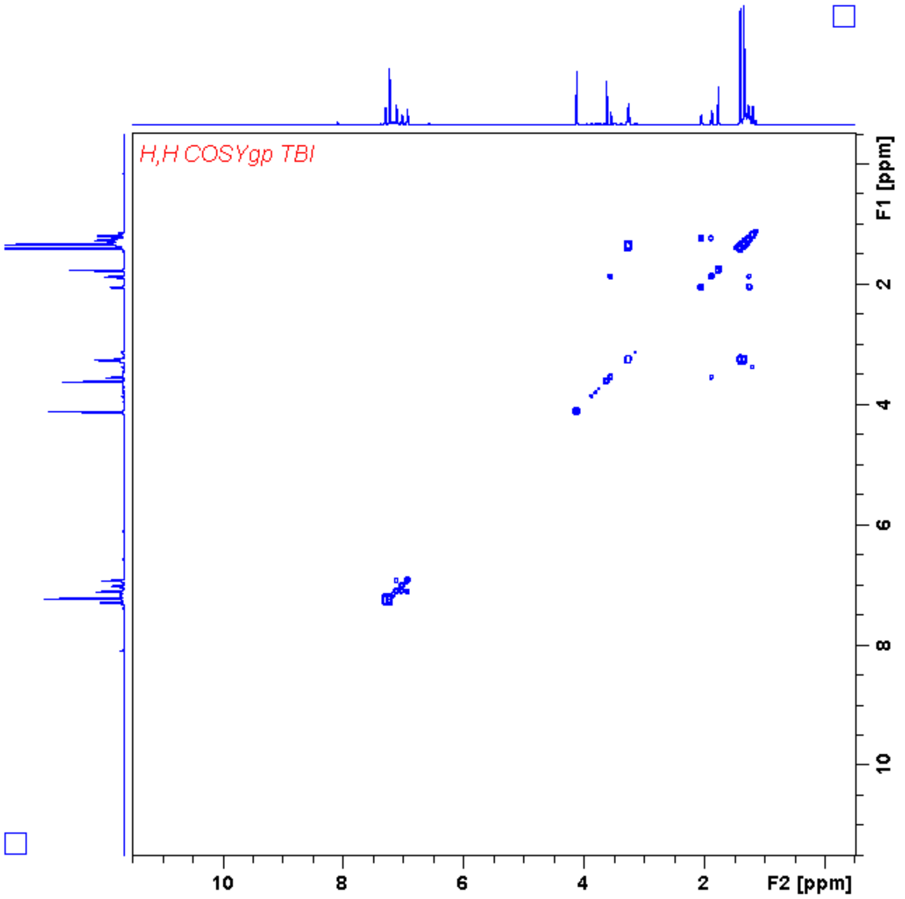

Figure S32. $^{1}H,^{1}H$(600MHz) COSY NMR spectrum of **11b** ([D$_8$]THF, 298 K).

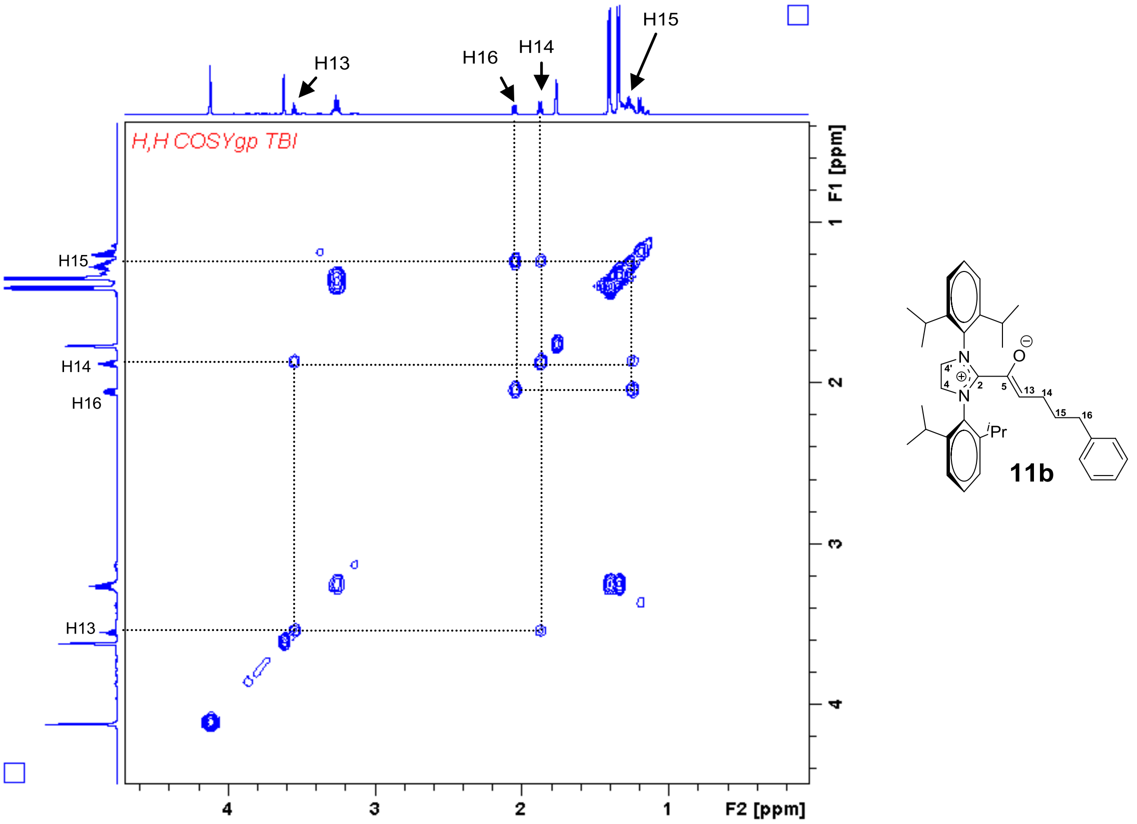

Figure S33. Part of $^{1}H,^{1}H$(600MHz) COSY NMR spectrum of **11b** ([D$_8$]THF, 298 K).

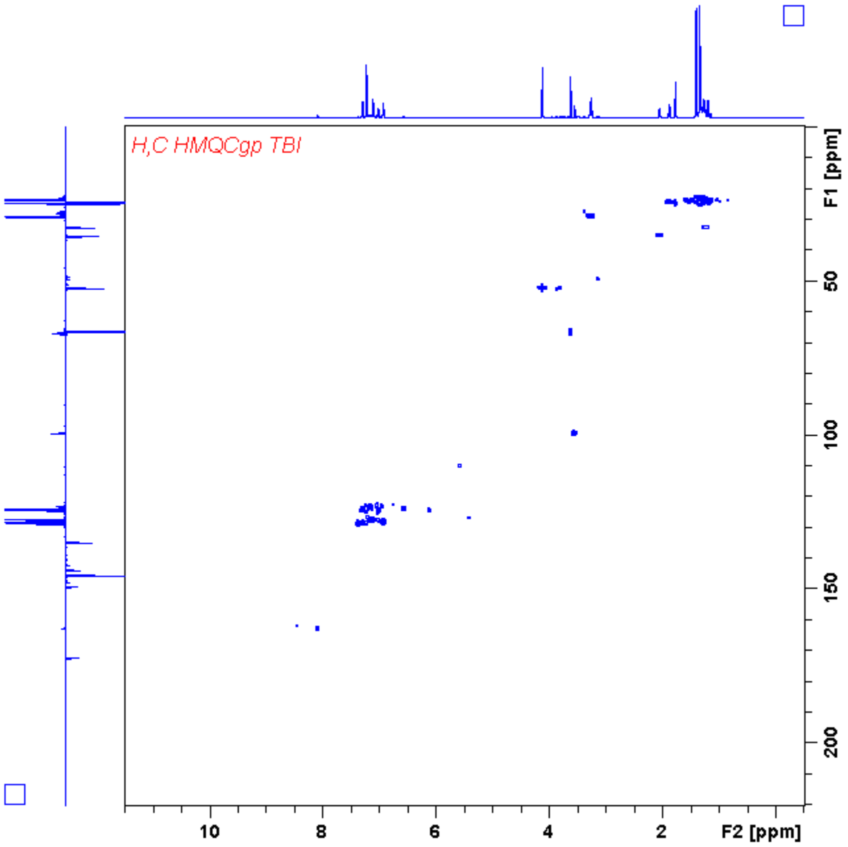

Figure S34. ^{1}H(600 MHz),^{13}C(150 MHz) HMQC NMR spectrum of **11b** ([D8]THF, 298 K).

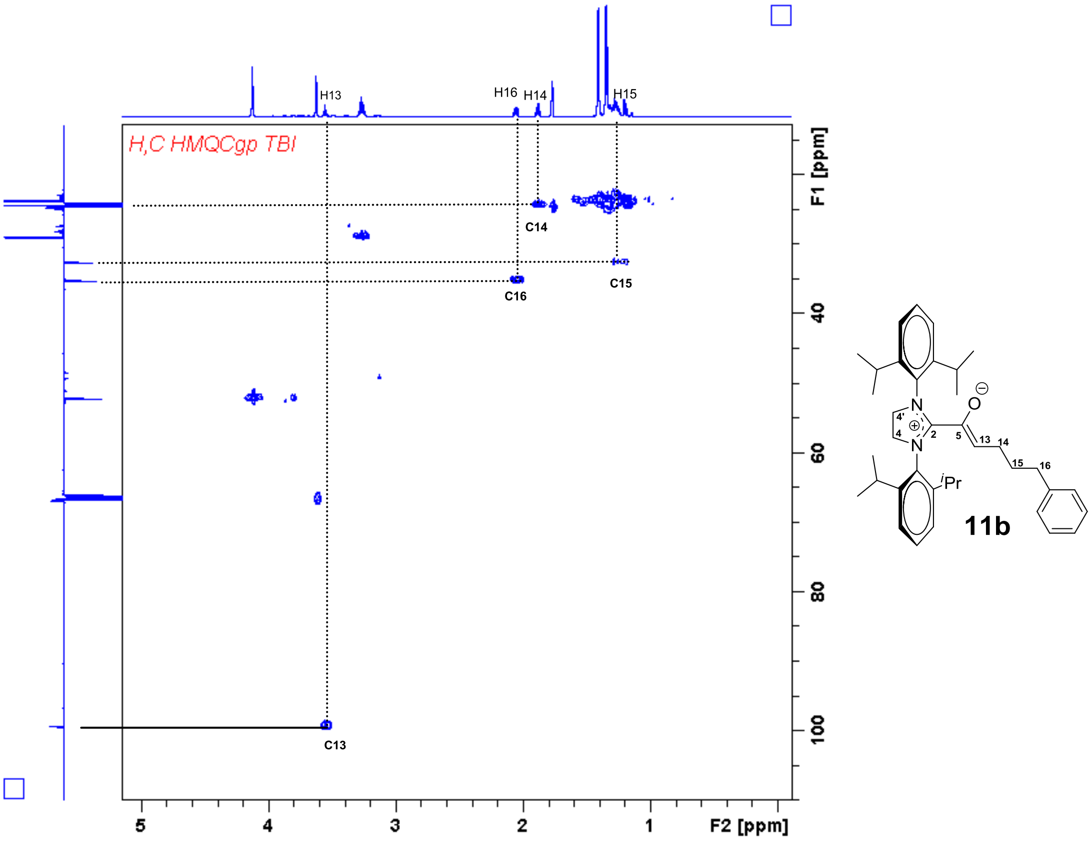

Figure S35. Part of ^{1}H(600 MHz),^{13}C(150 MHz) HMQC NMR spectrum of **11b** ([D8]THF, 298 K).

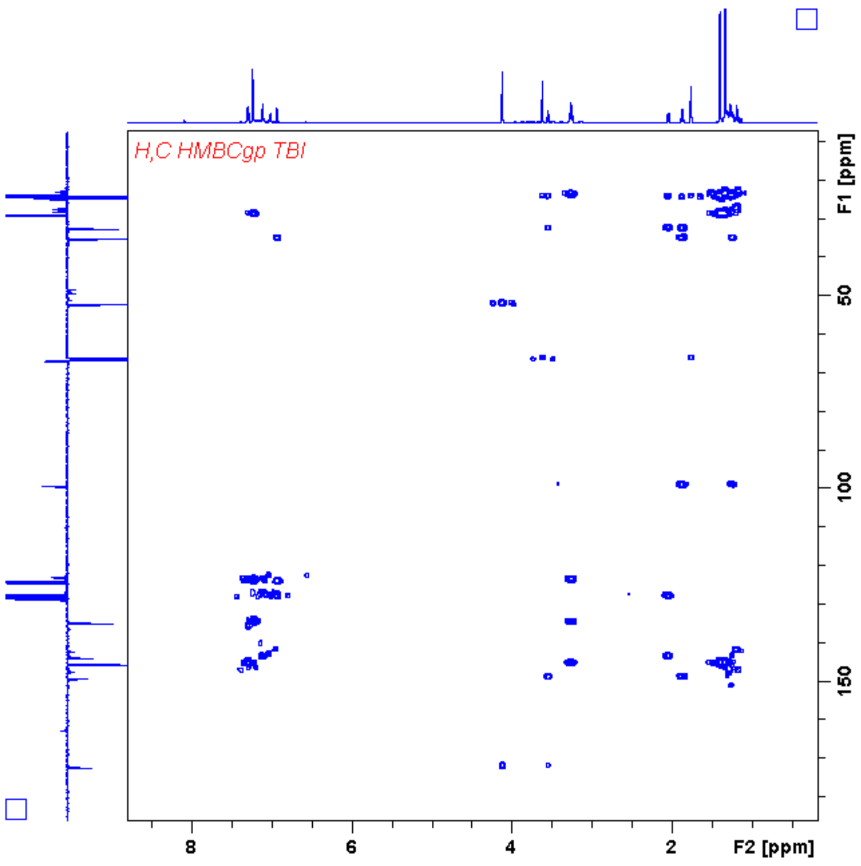

Figure S36. ^{1}H(600 MHz),^{13}C(150 MHz) HMBC NMR spectrum of **11b** ([D8]THF, 298 K).

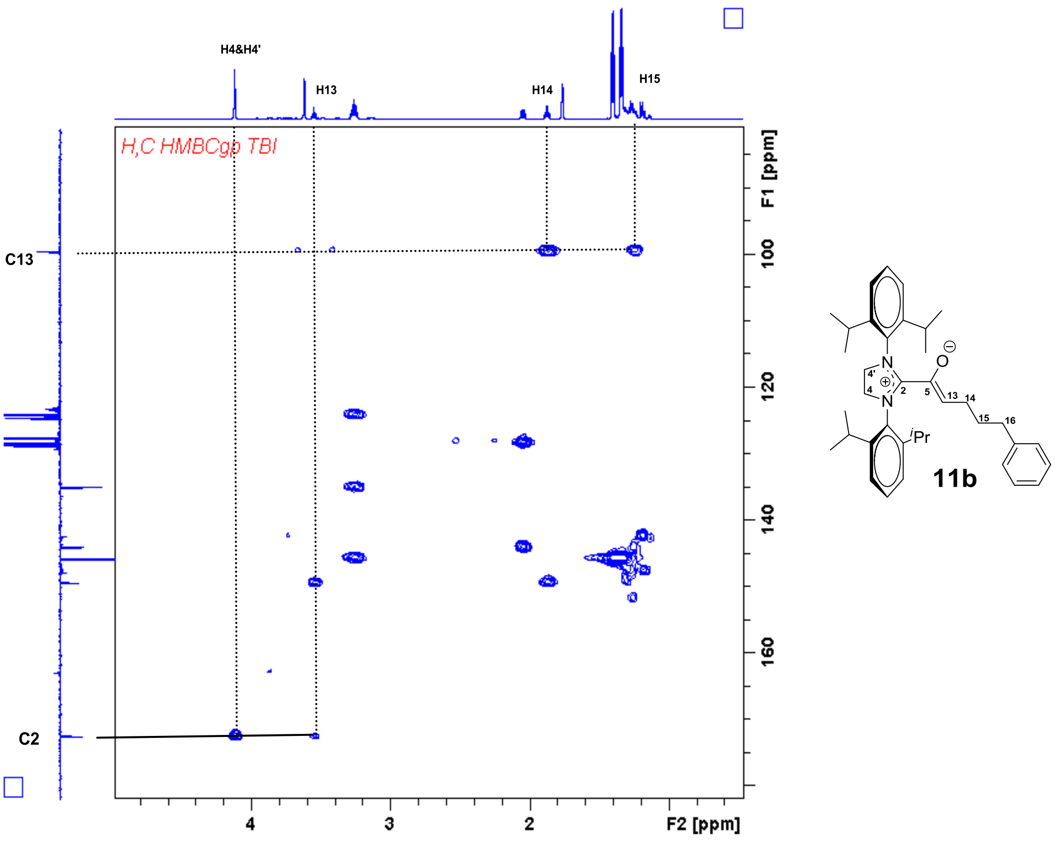

Figure S37. Part of ^{1}H(600 MHz),^{13}C(150 MHz) HMBC NMR spectrum of **11b** ([D8]THF, 298 K).

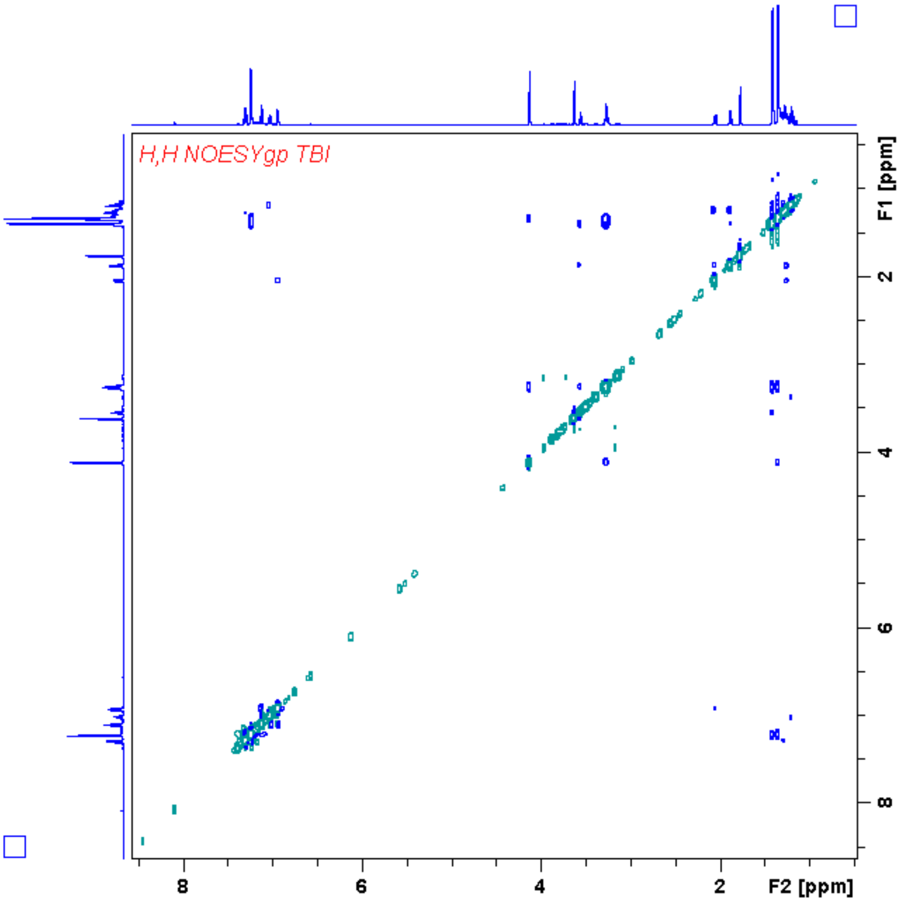

Figure S38. ^{1}H,^{1}H NOESY NMR spectrum of **11b** ([D8]THF, 600 MHz, 298 K, mixing time = 600 ms).

12.6 ^{1}H NMR spectrum of the 2,2-diamino dienol (1)

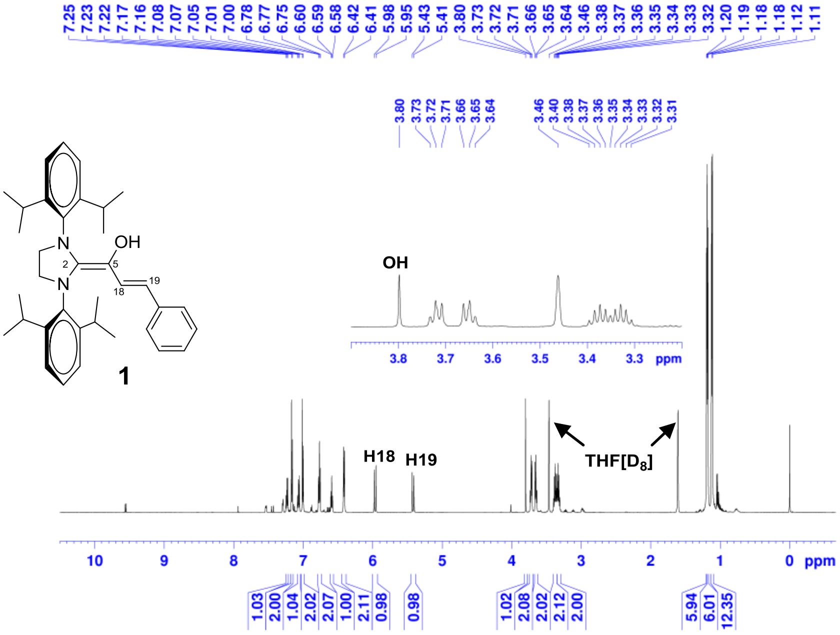

Figure S39. ^{1}H(600 MHz) NMR spectrum of **1** ([D8]THF, 298 K).

12.7 1D and 2D NMR spectra of the Michael addition product 4a

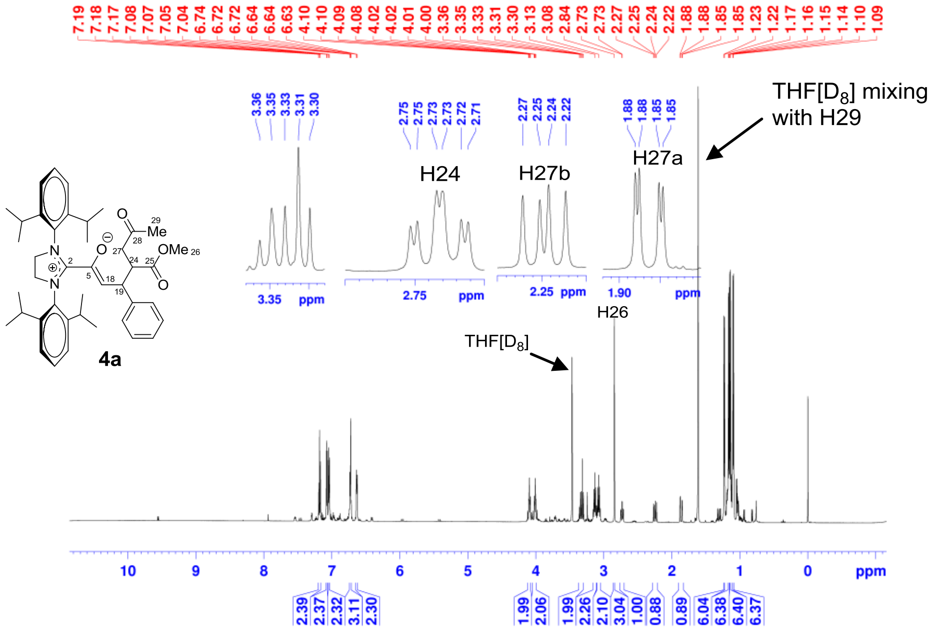

Figure S40. ^{1}H NMR(600 MHz) NMR spectrum of **4a** ([D$_8$]THF, 298 K).

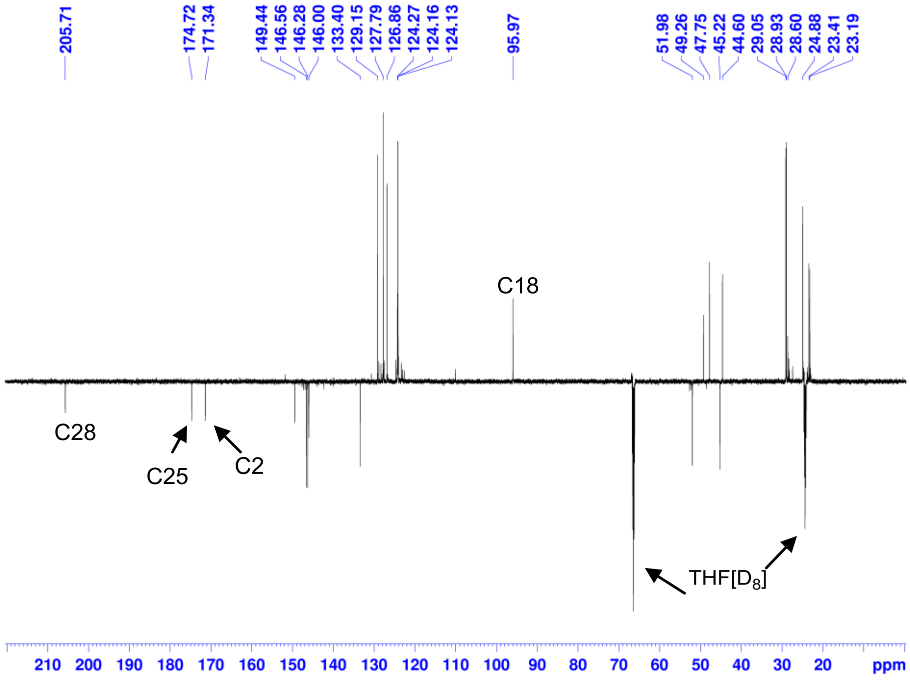

Figure S41. ^{13}C(150 MHz) DEPTQ NMR spectrum of **4a** ([D$_8$]THF, 298 K).

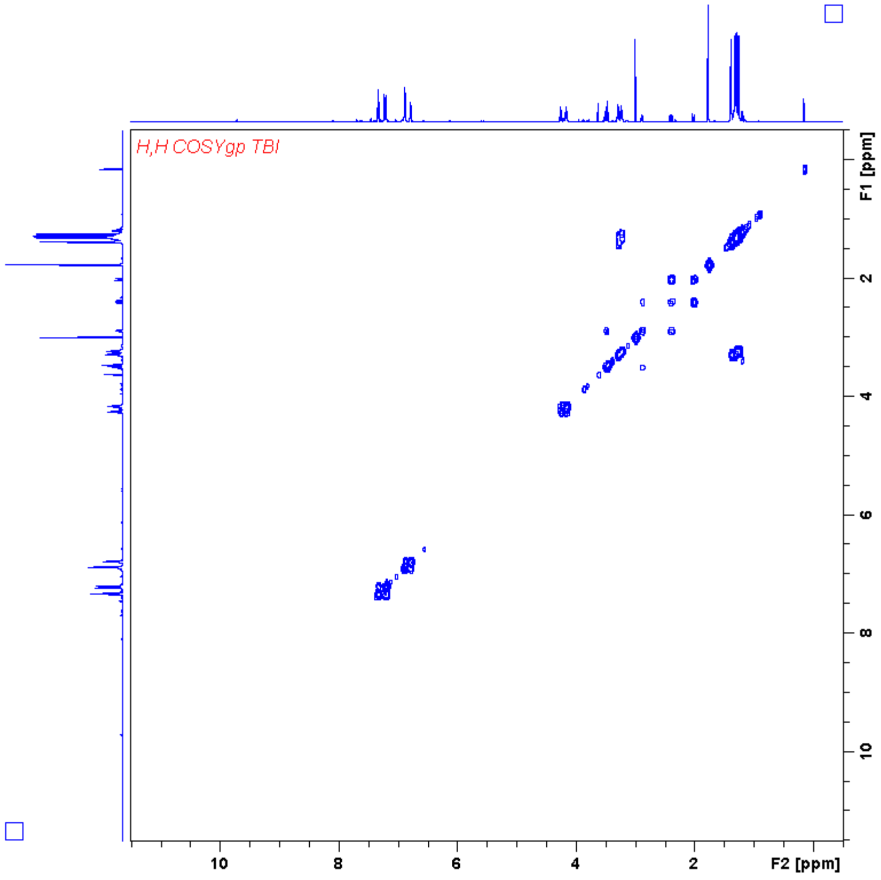

Figure S42. ^{1}H,^{1}H COSY(600MHz) NMR spectrum of **4a** ([D$_8$]THF, 298 K).

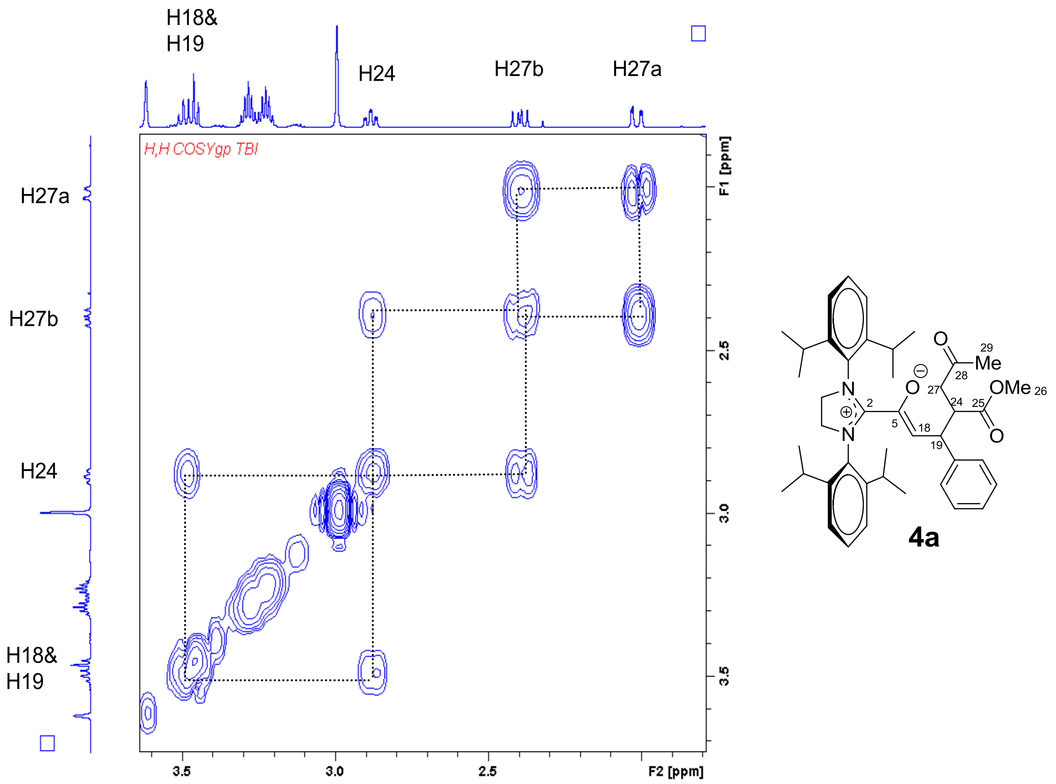

Figure S43. Part of the ^{1}H,^{1}H(600MHz) COSY NMR spectrum of **4a** ([D$_8$]THF, 298 K).

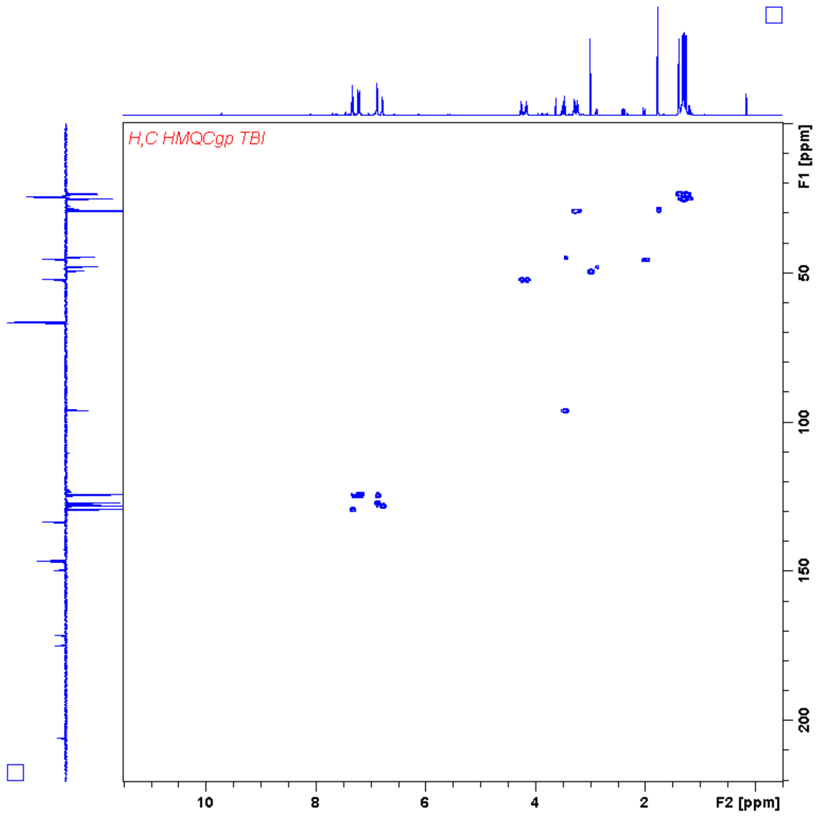

Figure S44. ^{1}H(600 MHz),^{13}C(150 MHz) HMQC NMR spectrum of **4a** ([D8]THF, 298 K).

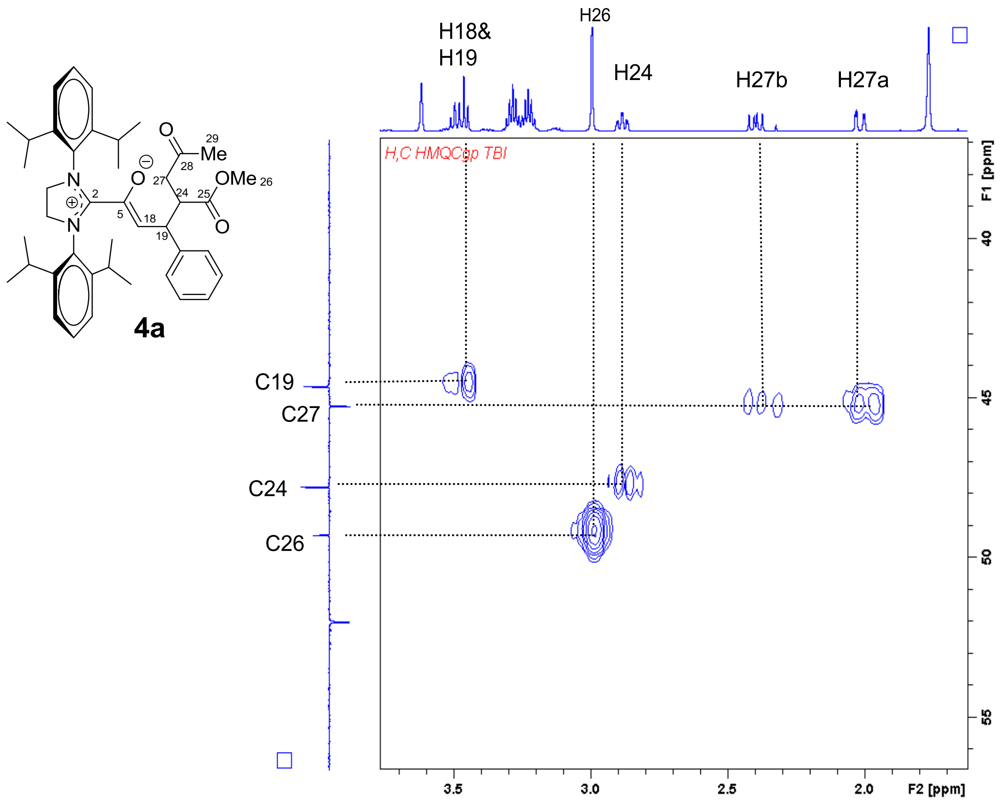

Figure S45. Part of the ^{1}H(600 MHz),^{13}C(150 MHz) HMQC NMR spectrum of **4a** ([D8]THF, 298 K).

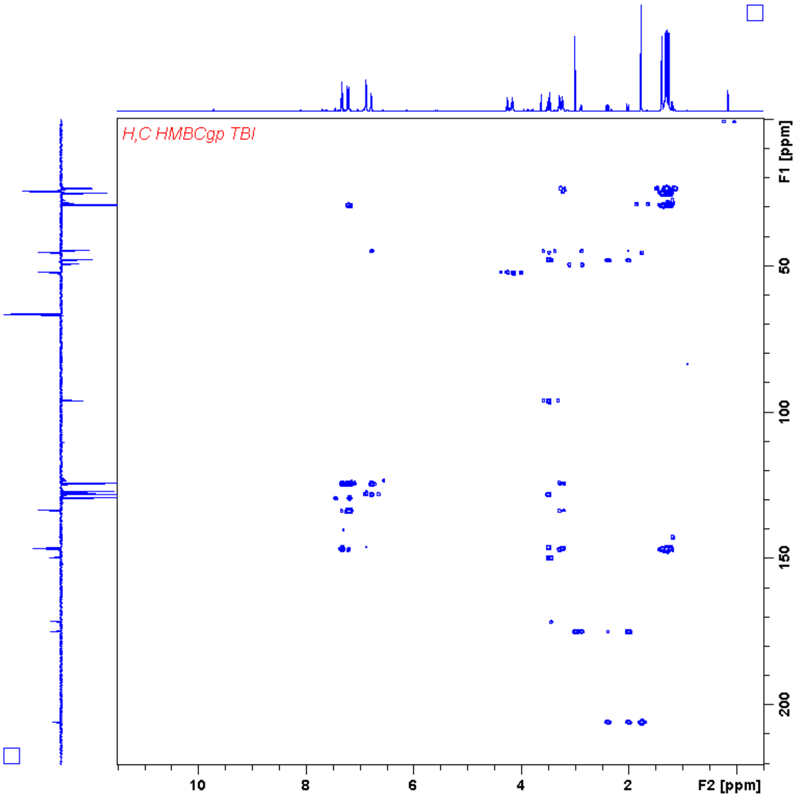

Figure S46. ^{1}H(600 MHz),^{13}C(150 MHz) HMBC NMR spectrum of **4a** ([D$_8$]THF, 298 K).

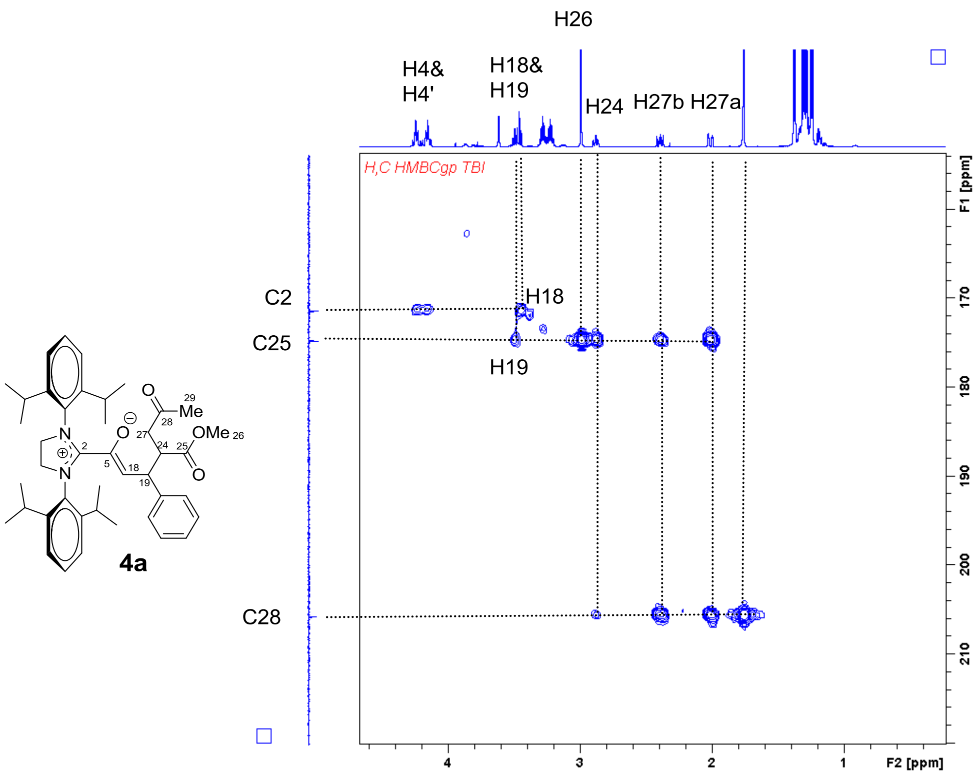

Figure S47. Part of the ^{1}H(600 MHz),^{13}C(150 MHz) HMBC NMR spectrum of **4a** ([D$_8$]THF, 298 K).

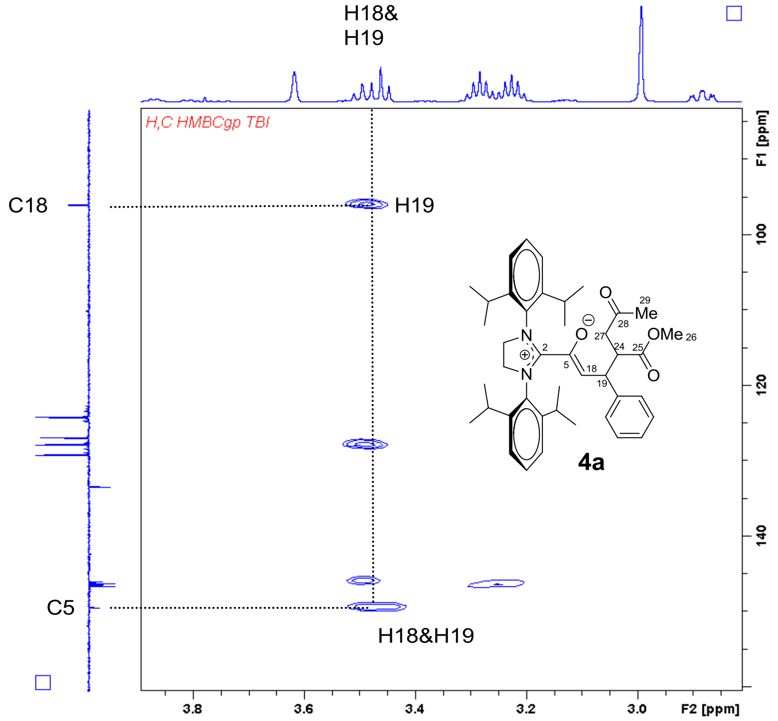

Figure S48. Part of the ^{1}H(600 MHz),^{13}C(150 MHz) HMBC NMR spectrum of **4a** ([D8]THF, 298 K).

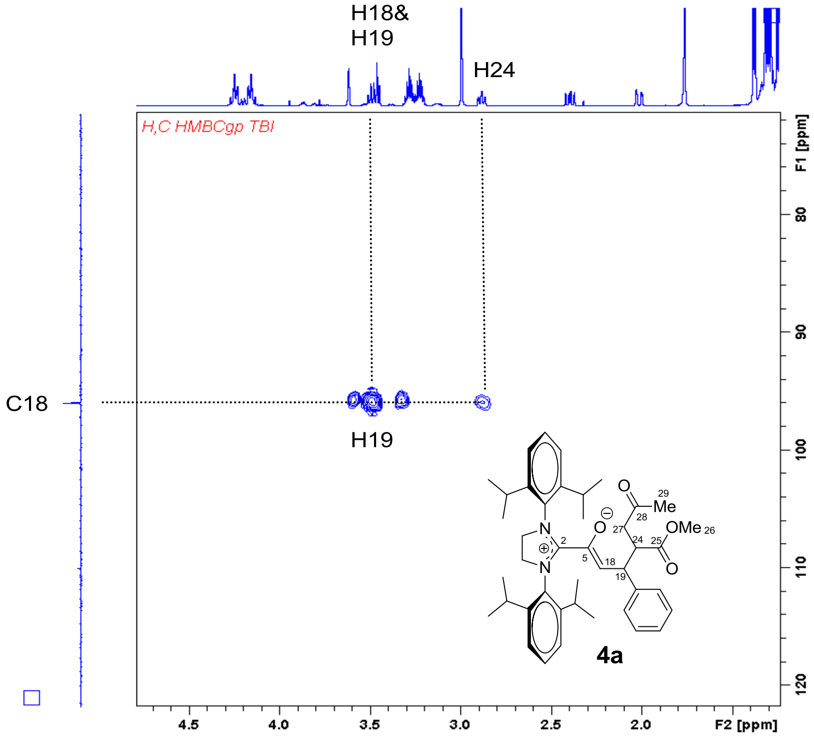

Figure S49. Part of the ^{1}H(600 MHz),^{13}C(150 MHz) HMBC NMR spectrum of **4a** ([D8]THF, 298 K).

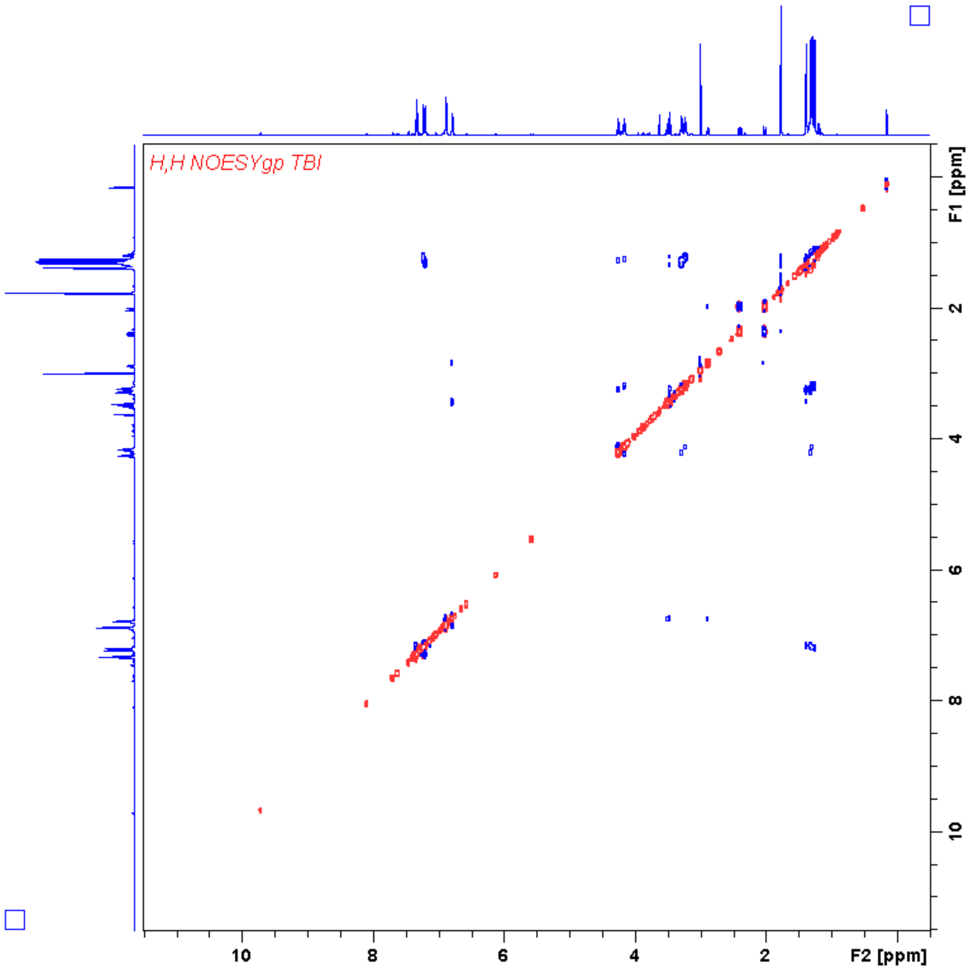

Figure S50. ^{1}H,^{1}H NOESY NMR spectrum of **4a** ([D8]THF, 600 MHz, 298 K, mixing time = 600 ms).

12.8 1D and 2D NMR spectra of the Michael addition product 4b-Et

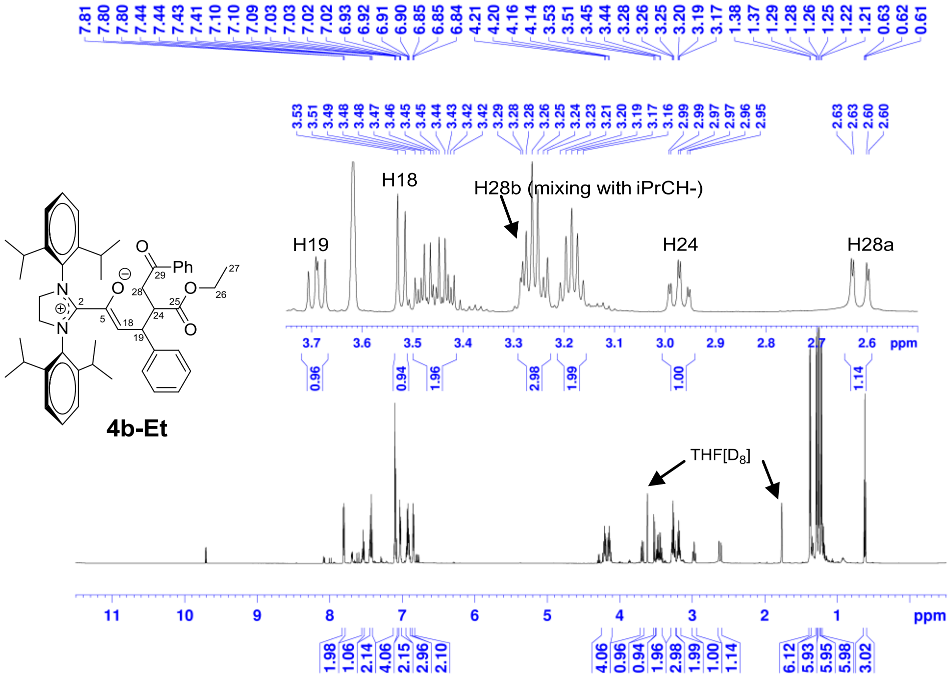

Figure S51. ^{1}H(600 MHz) NMR spectrum of **4b-Et** ([D$_8$]THF, 298 K).

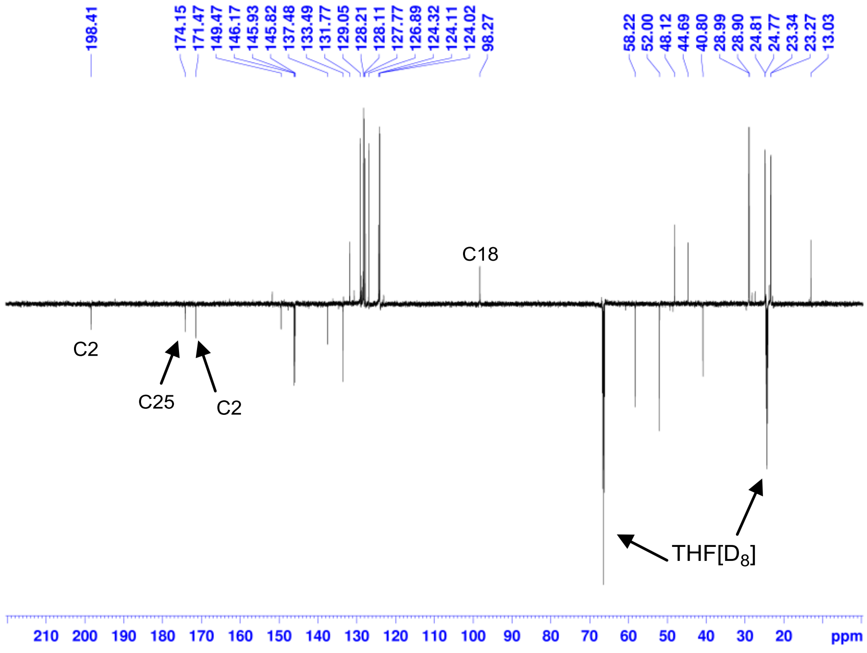

Figure S52. ^{13}C(150 MHz) DEPTQ NMR spectrum of **4b-Et** ([D$_8$]THF, 298 K).

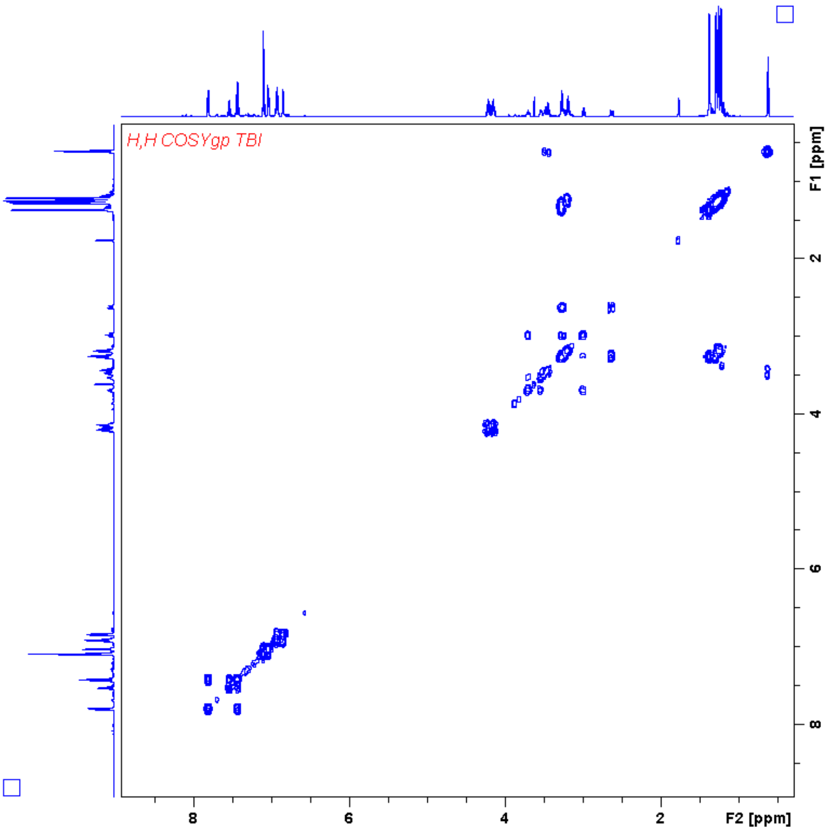

Figure S53. ^{1}H,^{1}H(600MHz) COSY NMR spectrum of **4b-Et** ([D$_8$]THF, 298 K).

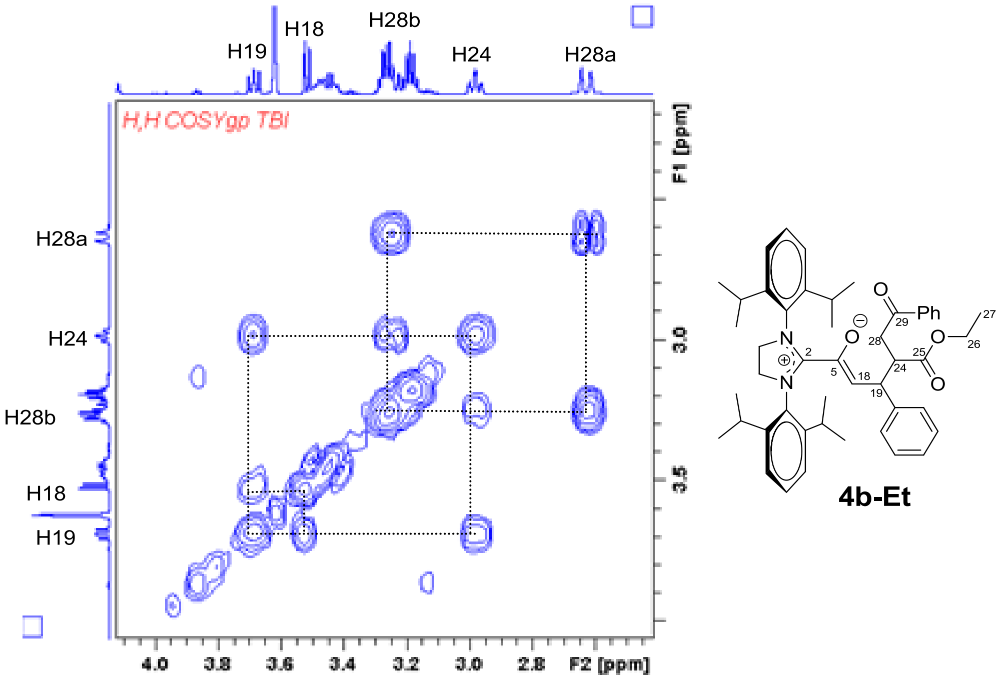

Figure S54. Part of the ^{1}H,^{1}H(600MHz) COSY NMR spectrum of **4b-Et** ([D$_8$]THF, 298 K).

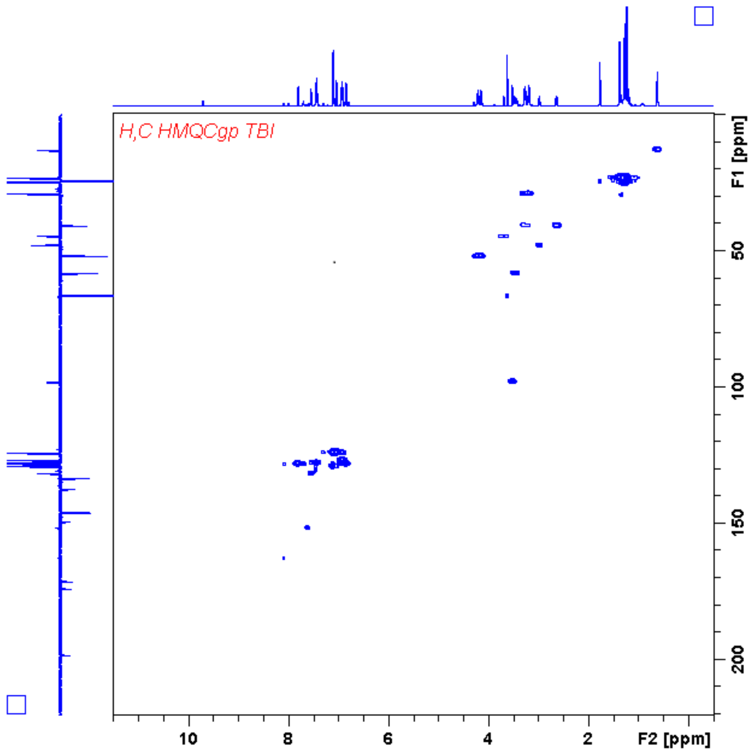

Figure S55. ^{1}H(600 MHz),^{13}C(150 MHz) HMQC NMR spectrum of **4b-Et** ([D8]THF, 298 K).

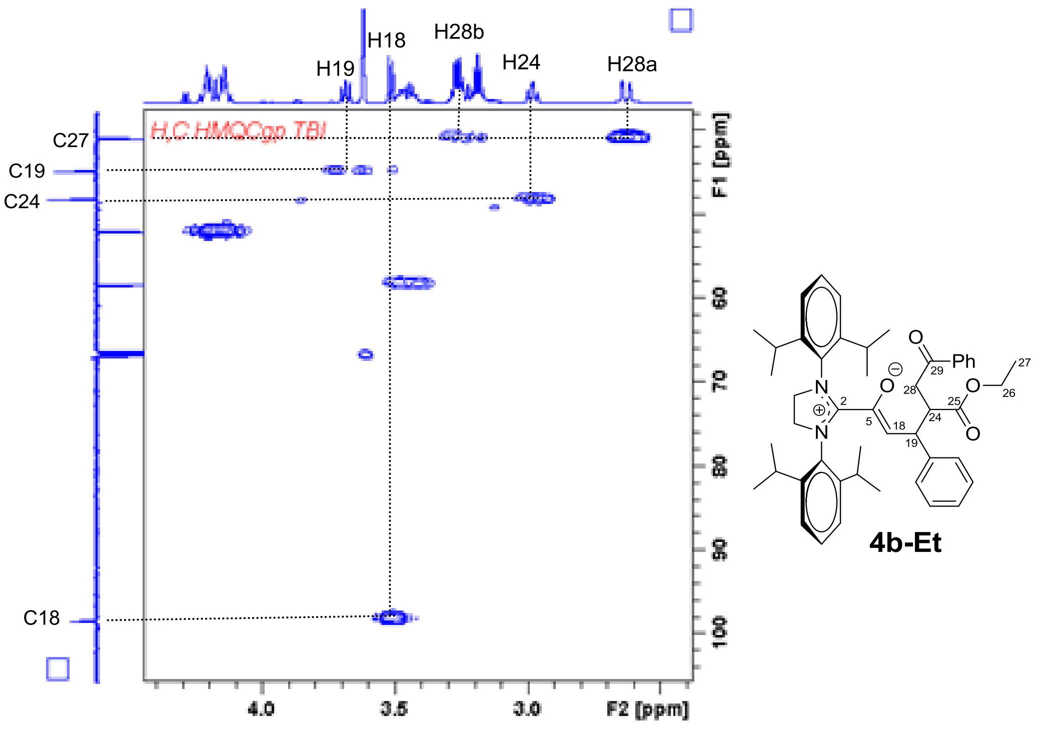

Figure S56. Part of the ^{1}H(600 MHz),^{13}C(150 MHz) HMQC NMR spectrum of **4b-Et** ([D8]THF, 298 K).

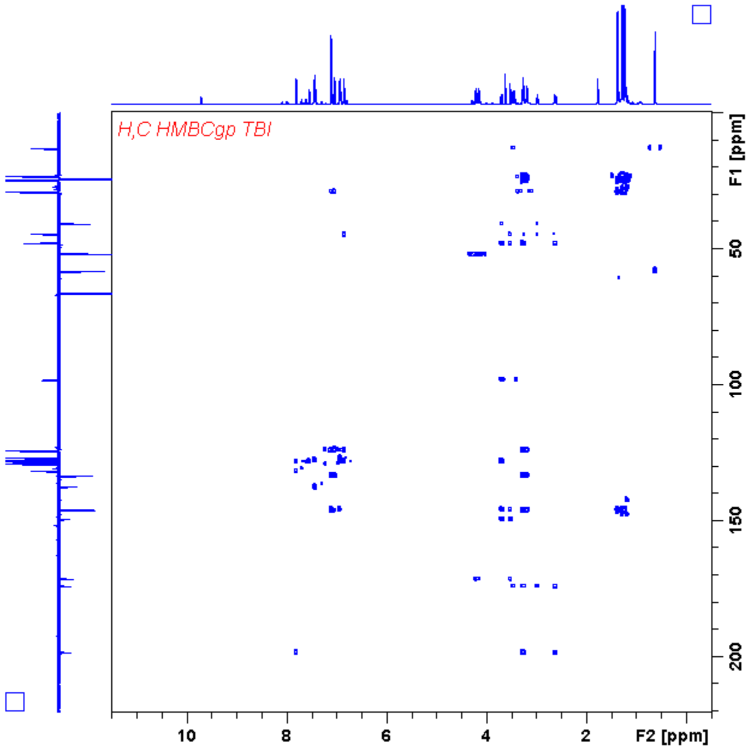

Figure S57. ^{1}H(600 MHz),^{13}C(150 MHz) HMBC NMR spectrum of **4b-Et** ([D8]THF, 298 K).

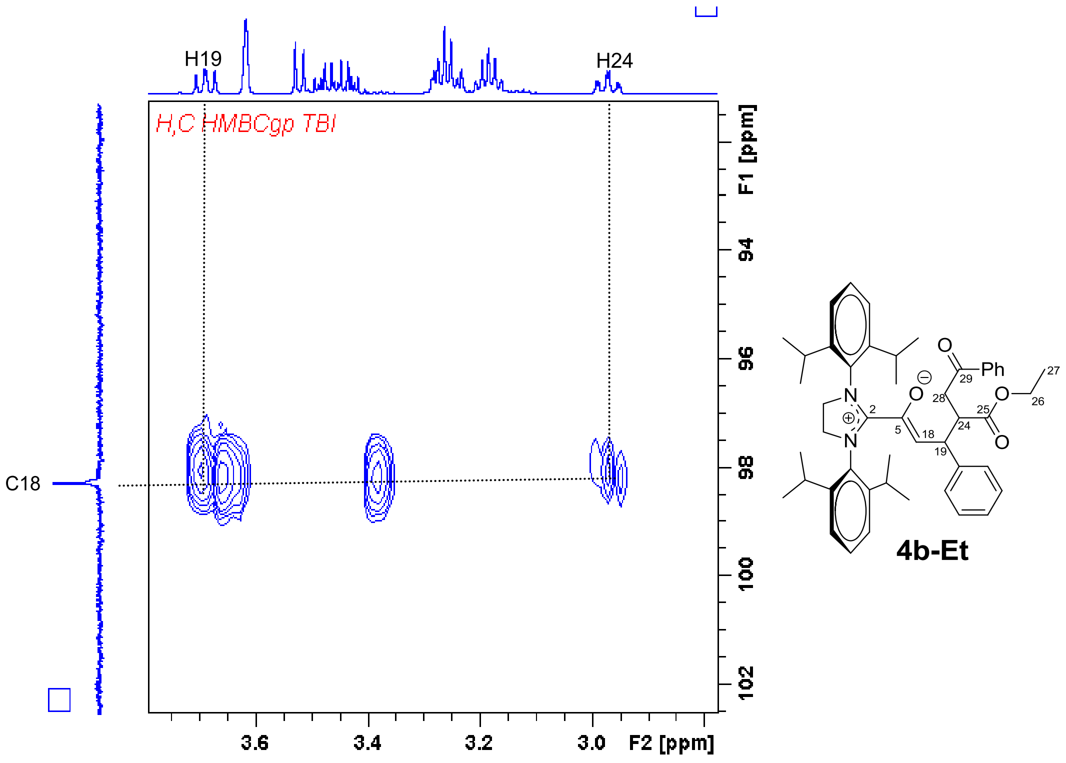

Figure S58. Part of the ^{1}H(600 MHz),^{13}C(150 MHz) HMBC NMR spectrum of **4b-Et** ([D_8]THF, 298 K).

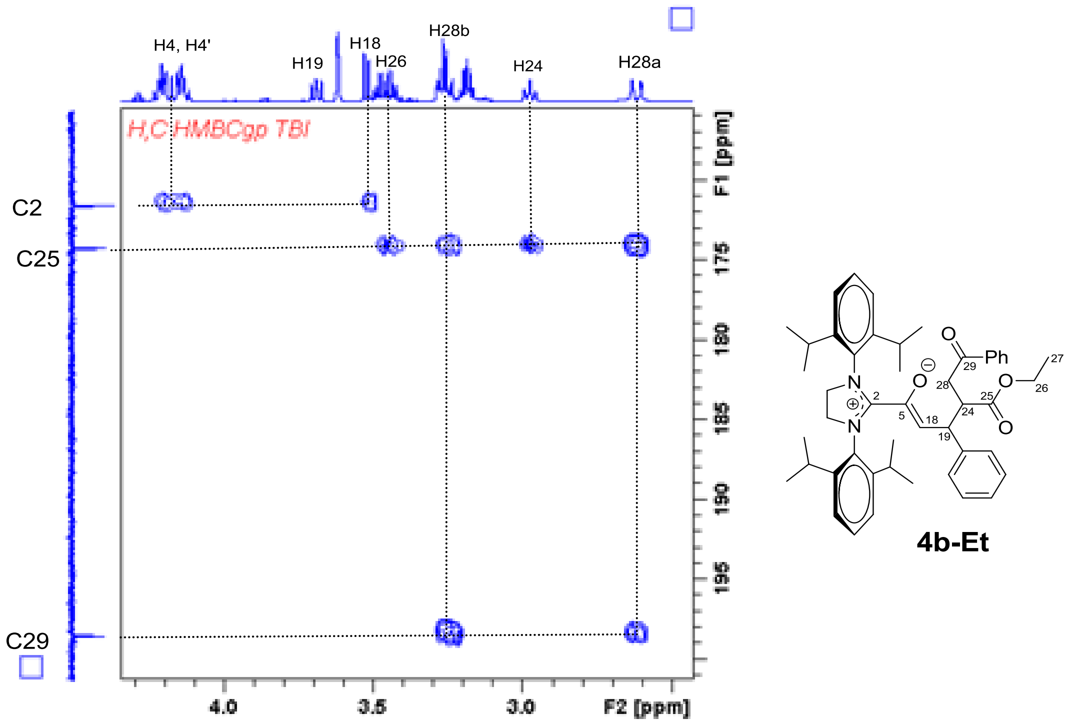

Figure S59. Part of the ^{1}H(600 MHz),^{13}C(150 MHz) HMBC NMR spectrum of **4b-Et** ([D8]THF, 298 K).

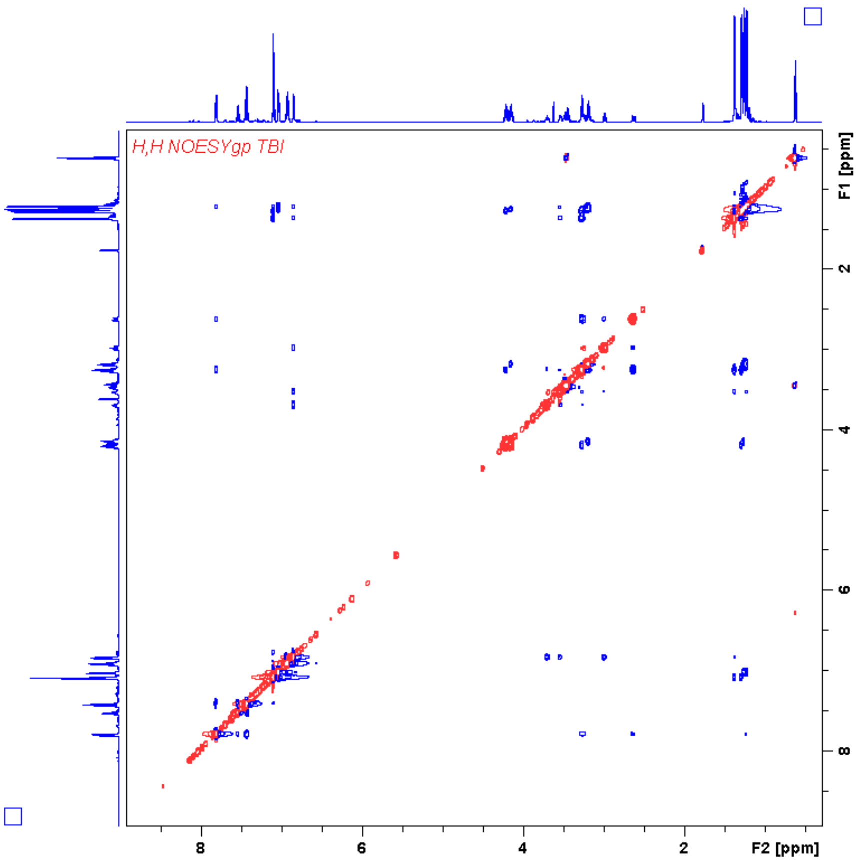

Figure S60. ^{1}H,^{1}H NOESY NMR spectrum of **4b-Et** ([D8]THF, 600 MHz, 298 K, mixing time = 600 ms).

12.9 1D and 2D NMR spectra of the Michael addition product 4c

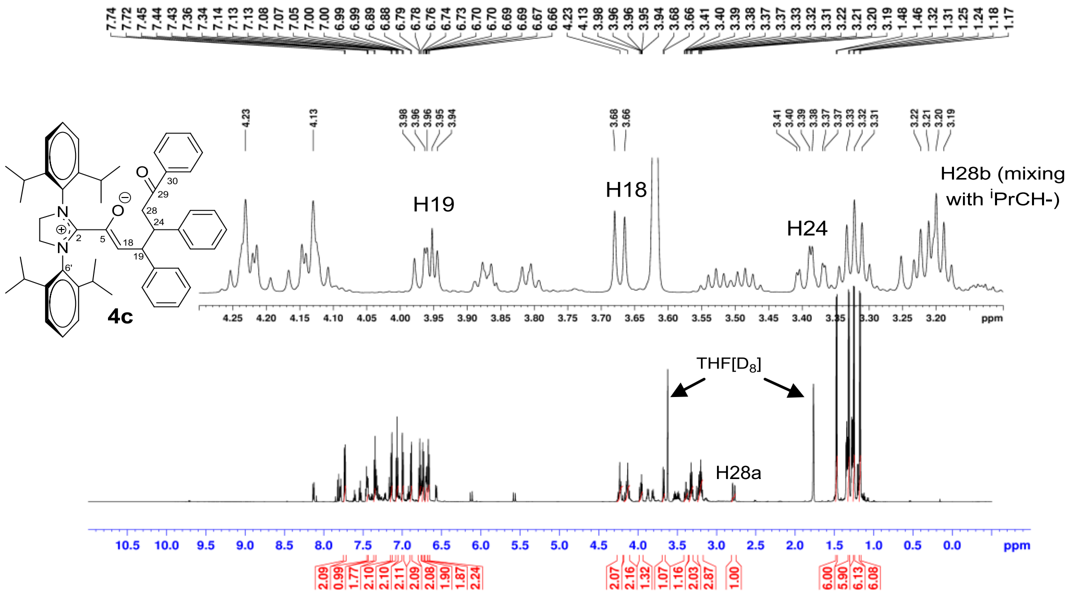

Figure S61. ^{1}H(600 MHz) NMR spectrum of **4c** (containing a minor amount of the 2,2-diamino dienol **1** and *E*-chalcone) ([D$_8$]THF, 298 K).

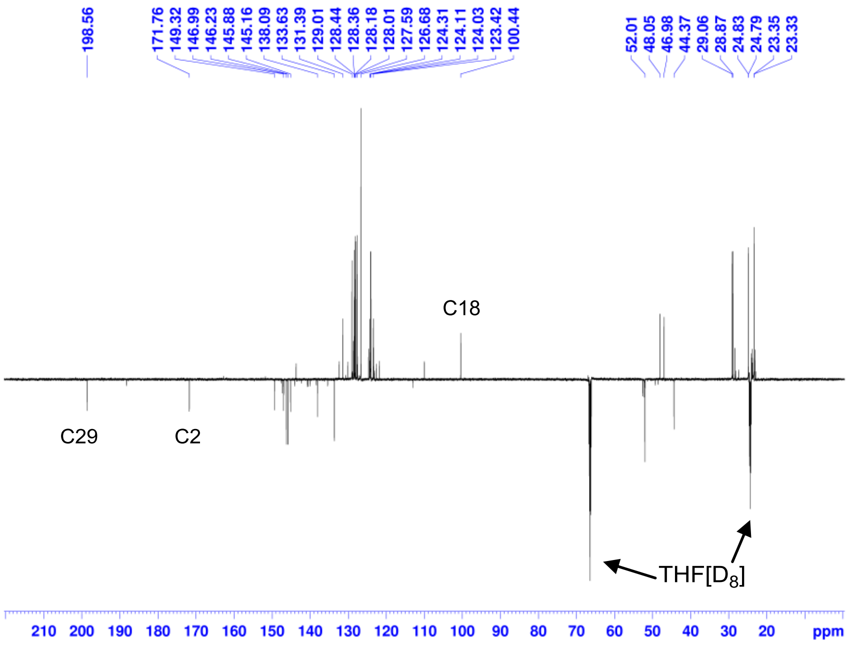

Figure S62. ^{13}C(150 MHz) DEPTQ NMR spectrum of **4c** (containing a minor amount of the 2,2-diamino dienol **1** and *E*-chalcone) ([D$_8$]THF, 298 K).

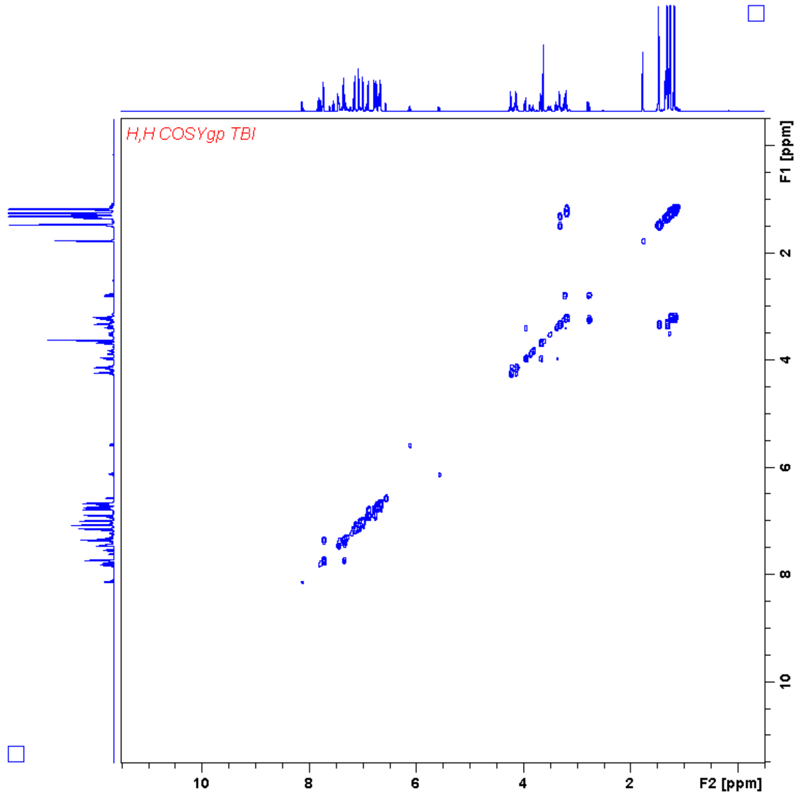

Figure S63. ^{1}H,^{1}H COSY(600MHz) NMR spectrum of **4c** (containing a minor amount of the 2,2-diamino dienol **1** and *E*-chalcone) ([D$_8$]THF, 298 K).

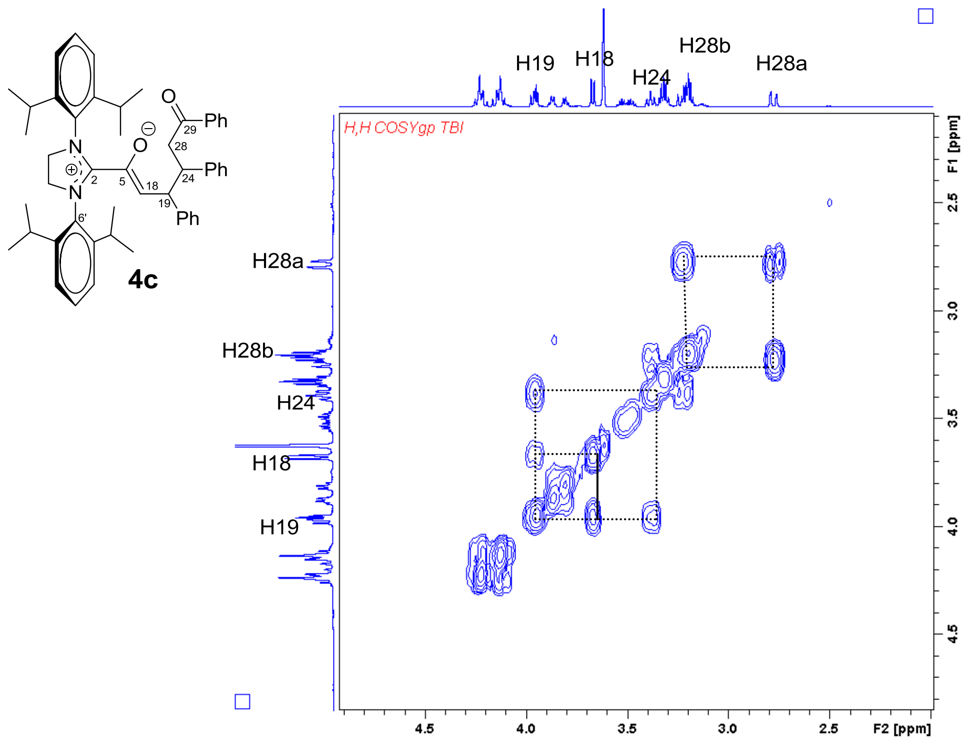

Figure S64. Part of ^{1}H,^{1}H COSY(600MHz) NMR spectrum of **4c** (containing a minor amount of the 2,2-diamino dienol **1** and *E*-chalcone) ([D$_8$]THF, 298 K).

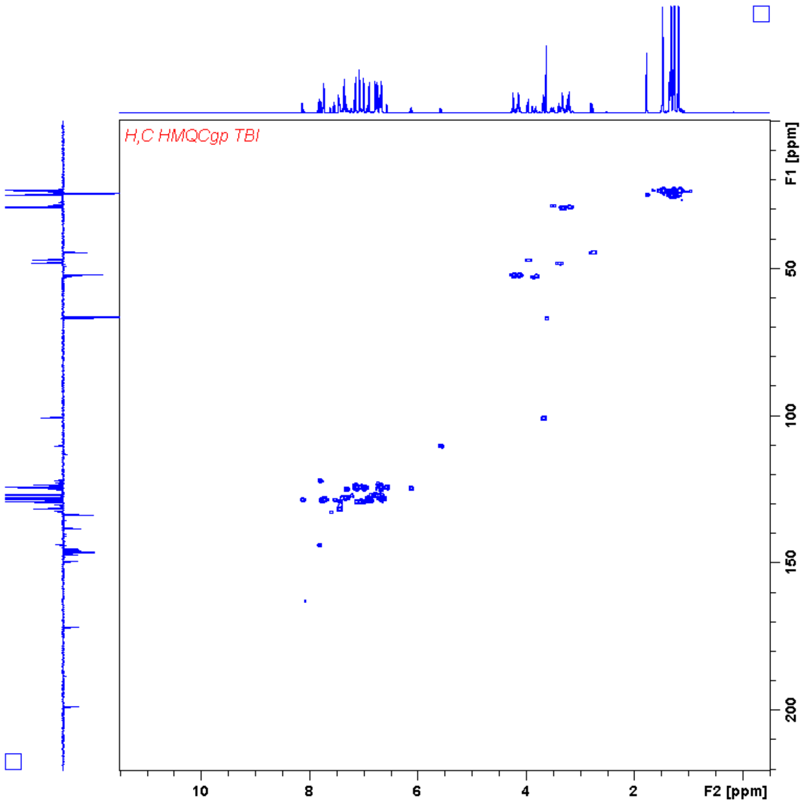

Figure S65. ^{1}H(600 MHz),^{13}C(150 MHz) HMQC NMR spectrum of **4c** (containing a minor amount of the 2,2-diamino dienol **1** and *E*-chalcone) ([D$_8$]THF, 298 K).

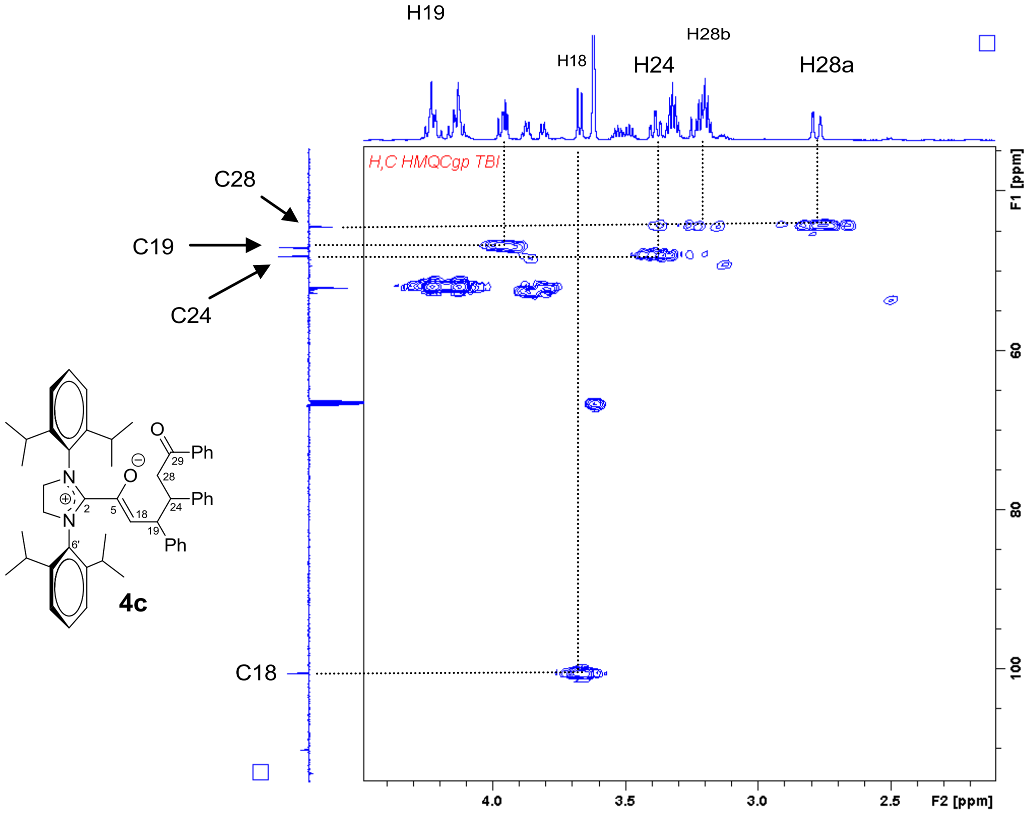

Figure S66. Part of ^{1}H(600 MHz),^{13}C(150 MHz) HMQC NMR spectrum of **4c** (containing a minor amount of the 2,2-diamino dienol **1** and *E*-chalcone) ([D$_8$]THF, 298 K).

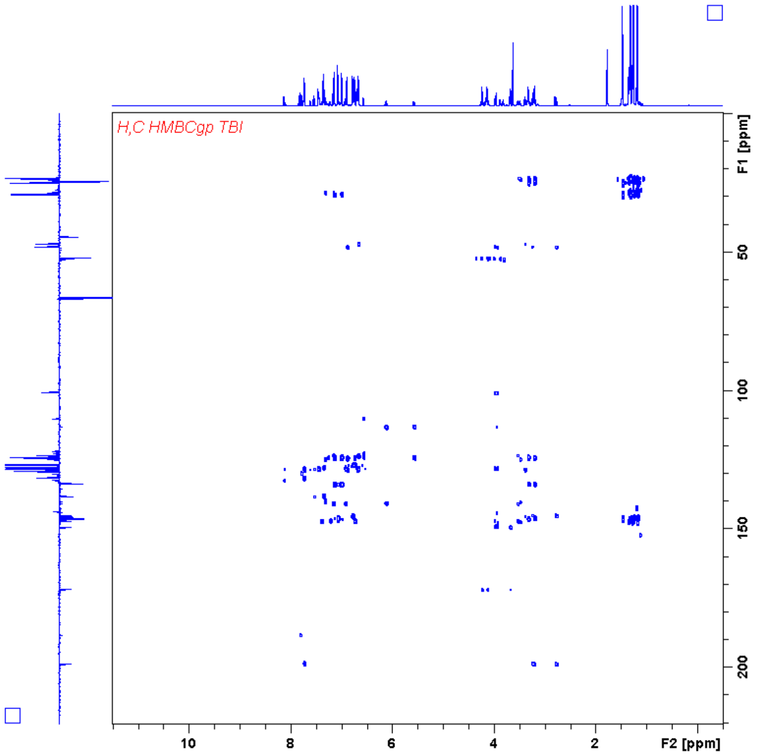

Figure S67. ^{1}H(600 MHz),^{13}C(150 MHz) HMBC NMR spectrum of **4c** (containing a minor amount of the 2,2-diamino dienol **1** and *E*-chalcone) ([D$_8$]THF, 298 K).

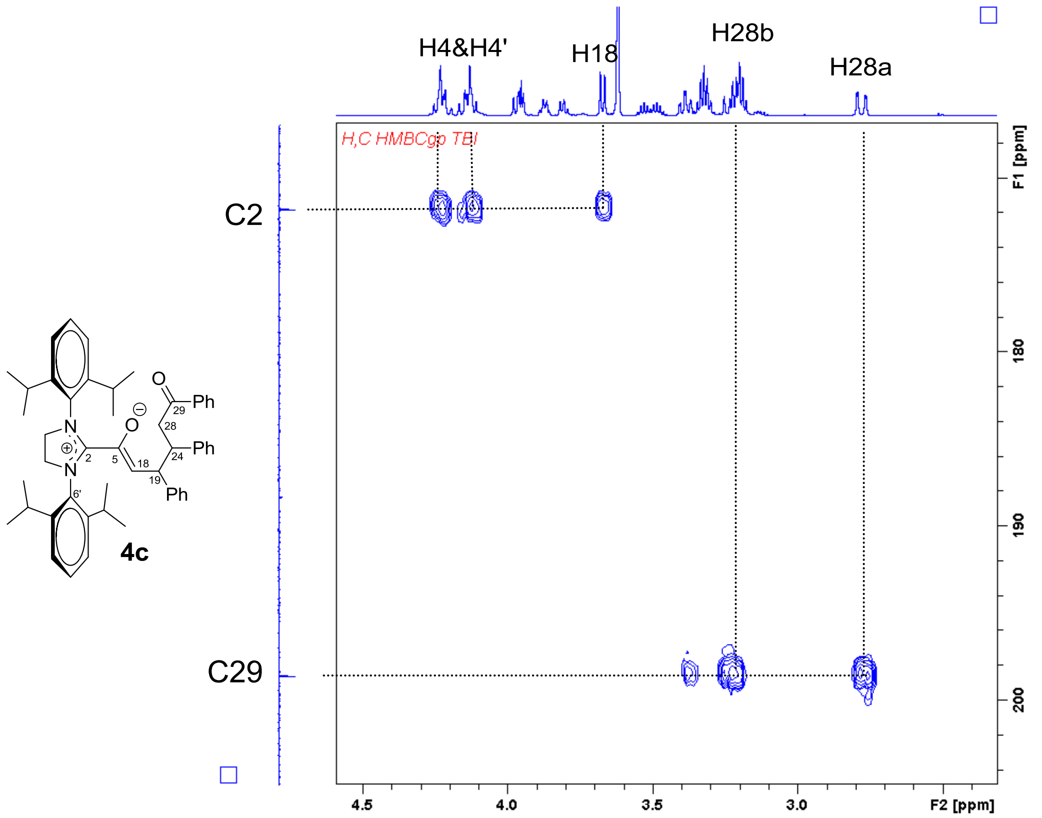

Figure S68. Part of the ^{1}H(600 MHz),^{13}C(150 MHz) HMBC NMR spectrum of **4c** (containing a minor amount of the 2,2-diamino dienol **1** and *E*-chalcone) ([D$_8$]THF, 298 K).

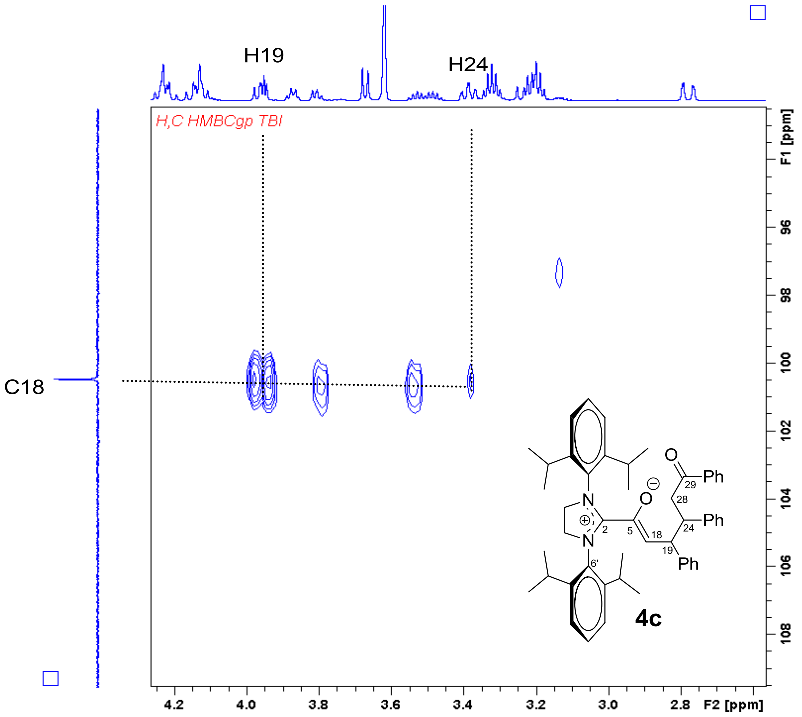

Figure S69. Part of the ^{1}H(600 MHz),^{13}C(150 MHz) HMBC NMR spectrum of **4c** (containing a minor amount of the 2,2-diamino dienol **1** and *E*-chalcone) ([D8]THF, 298 K).

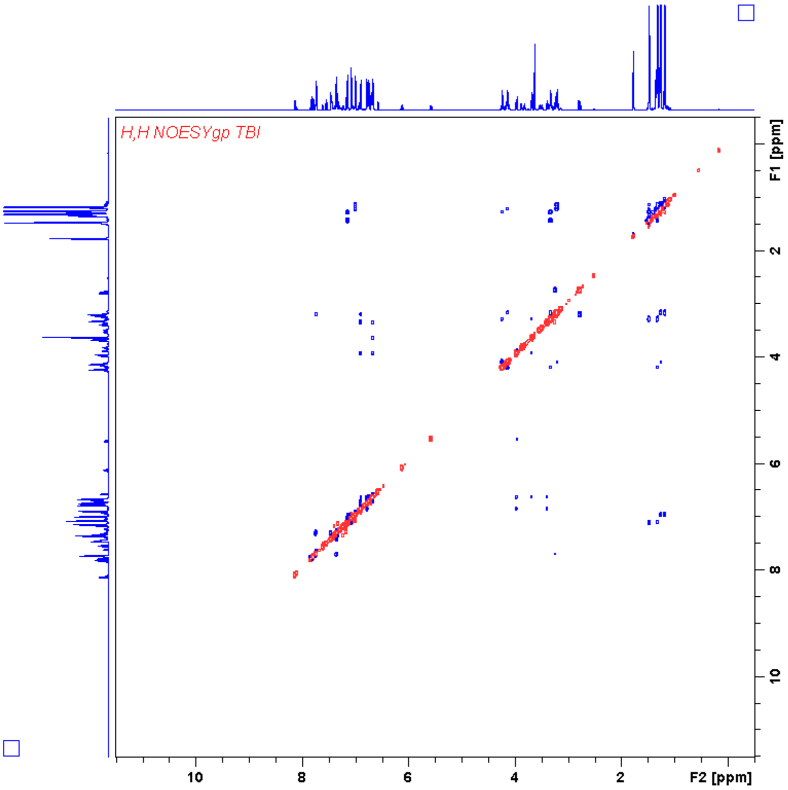

Figure S70. ^{1}H,^{1}H NOESY NMR spectrum of **4c** (containing a minor amount of the 2,2-diamino dienol **1** and *E*-chalcone) ([D8]THF, 600 MHz, 298 K, mixing time = 600 ms).

12.10 1D and 2D NMR spectra of the Michael addition product 4b-Me

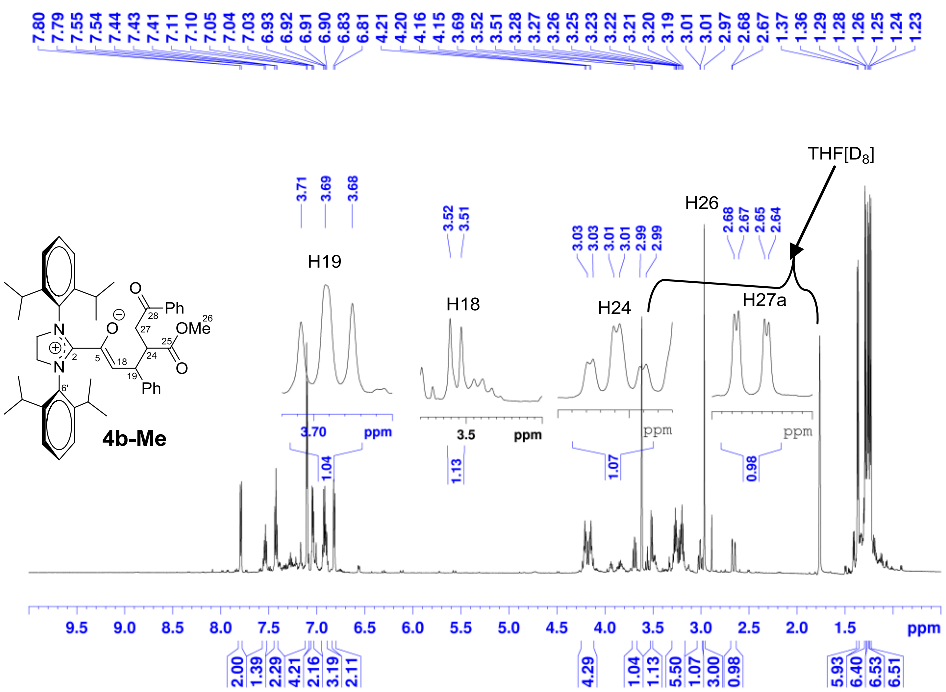

Figure S71. ^{1}H(600 MHz) NMR spectrum of **4b-Me** ([D8]THF, 298 K).

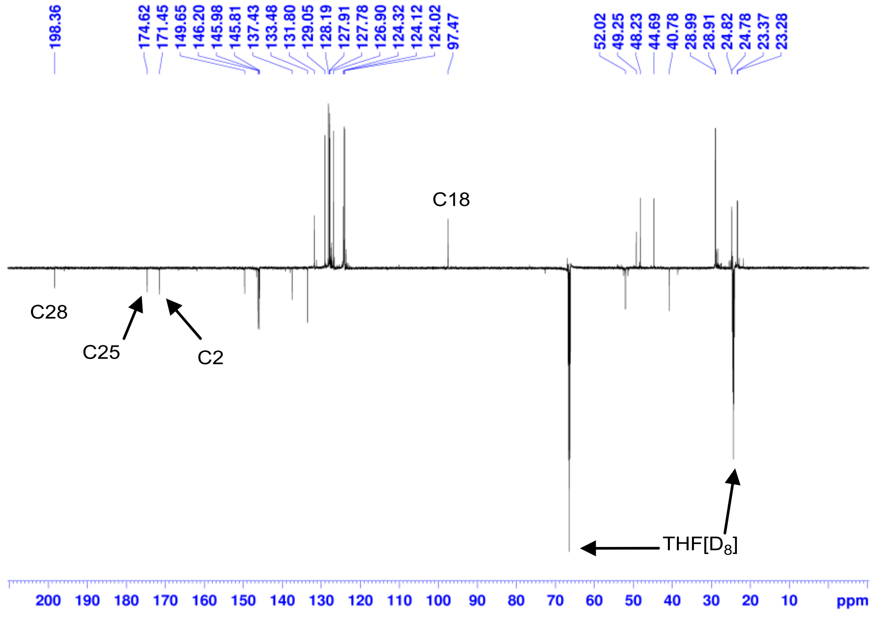

Figure S72. ^{13}C(150 MHz) DEPTQ NMR spectrum of **4b-Me** ([D8]THF, 298 K).

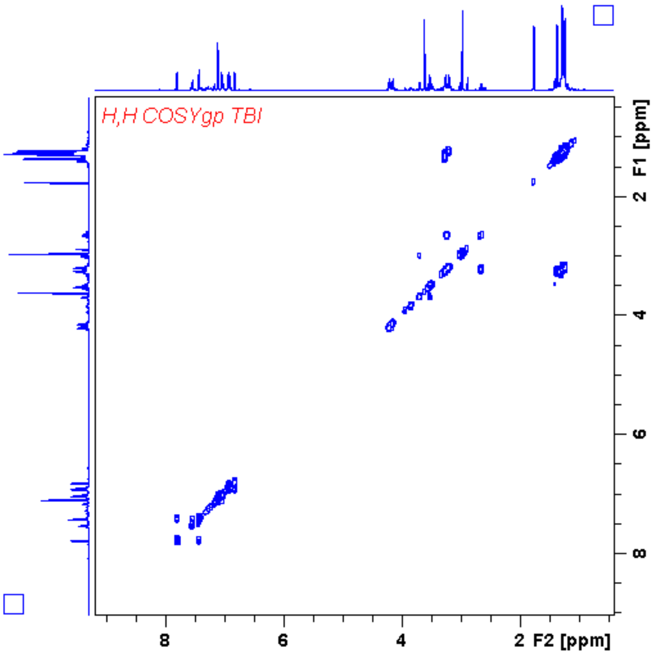

Figure S73. ^{1}H,^{1}H(600 MHz) COSY NMR spectrum of **4b-Me** ([D$_8$]THF, 298 K).

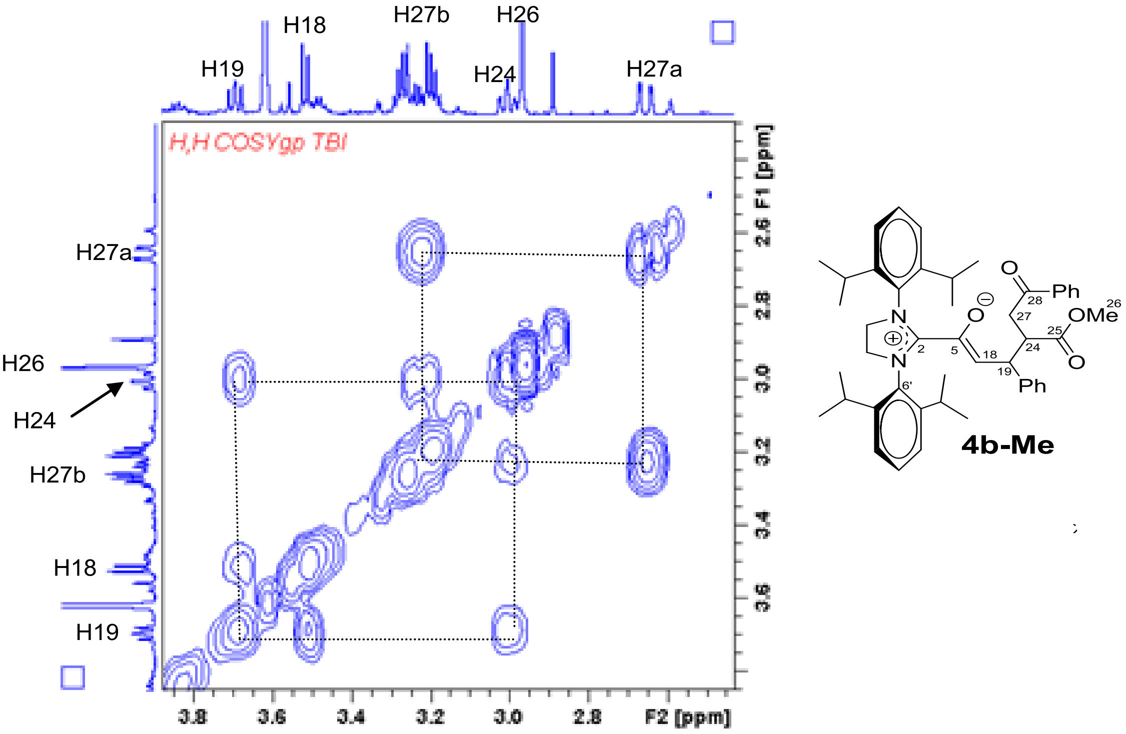

Figure S74. Part of the ^{1}H,^{1}H(600 MHz) COSY NMR spectrum of **4b-Me** ([D$_8$]THF, 298 K).

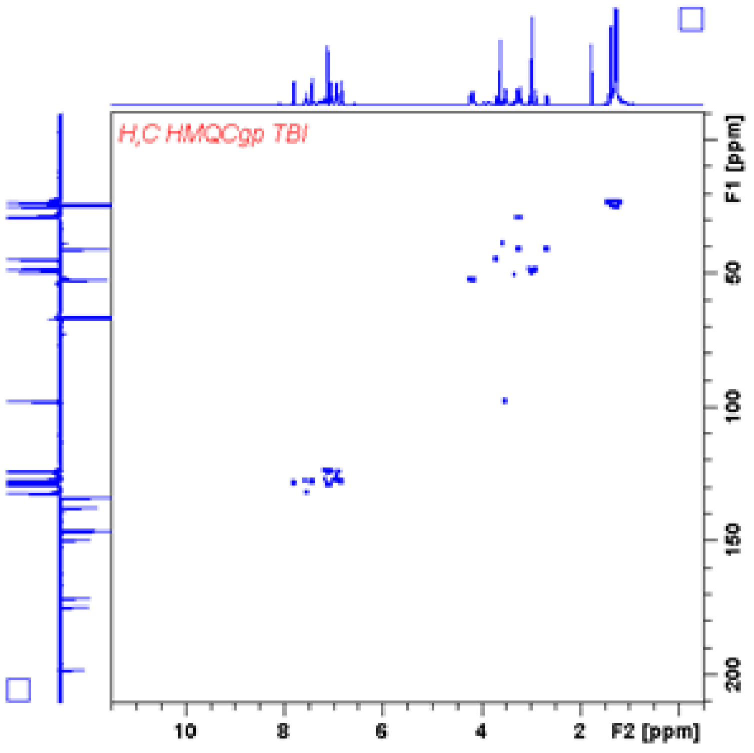

Figure S75. ^{1}H(600 MHz),^{13}C(150 MHz) HMQC NMR spectrum of **4b-Me** ([D8]THF, 298 K).

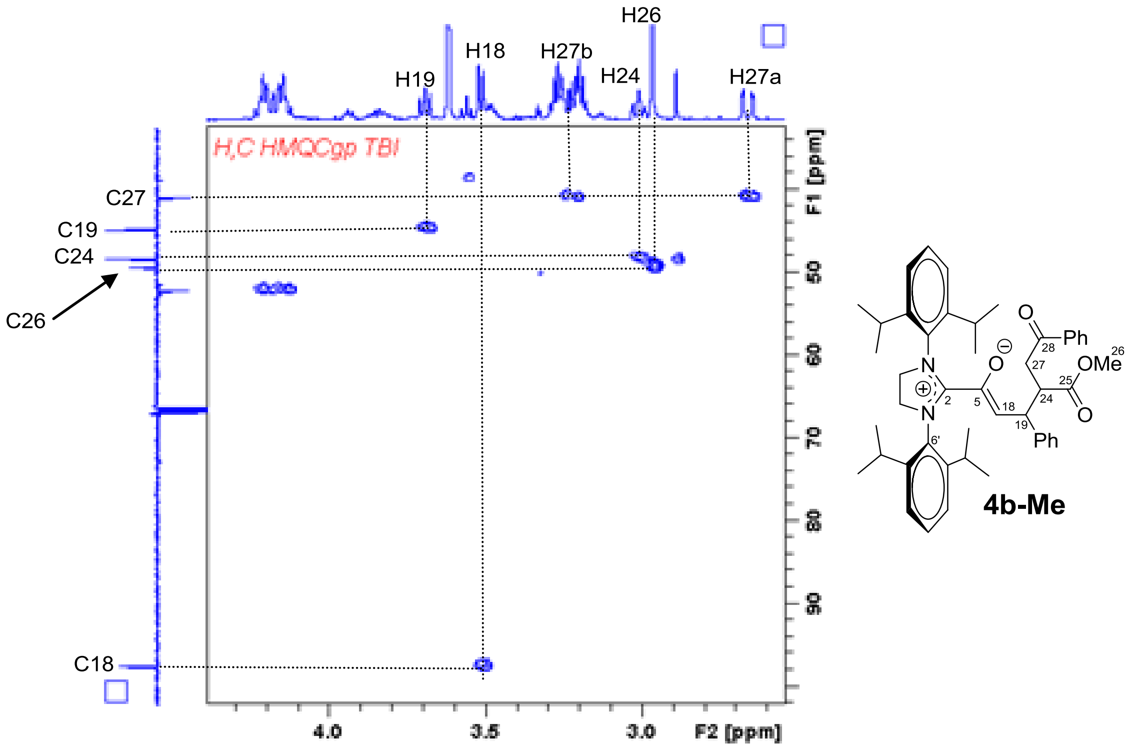

Figure S76. Part of the ^{1}H(600 MHz),^{13}C(150 MHz) HMQC NMR spectrum of **4b-Me** ([D8]THF, 298 K).

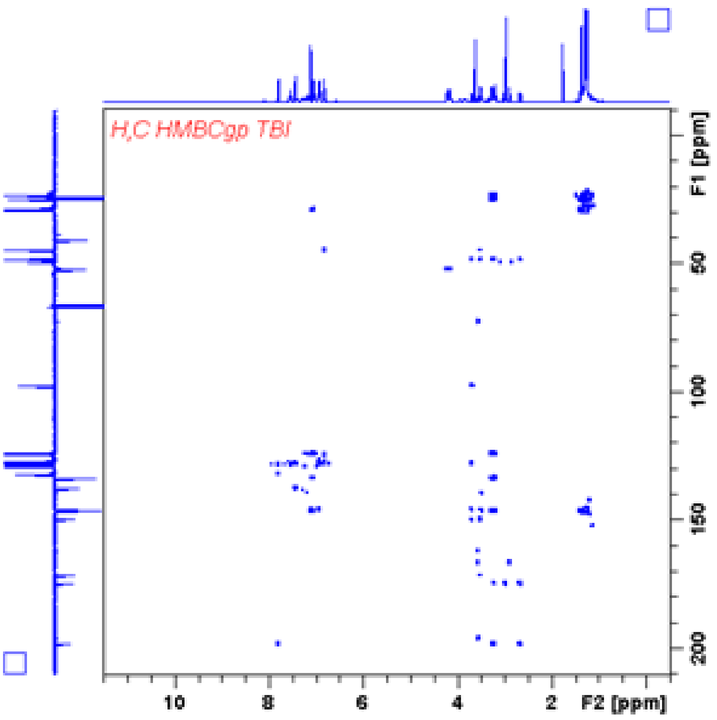

Figure S77. ^{1}H(600 MHz),^{13}C(150 MHz) HMBC NMR spectrum of **4b-Me** ([D8]THF, 298 K).

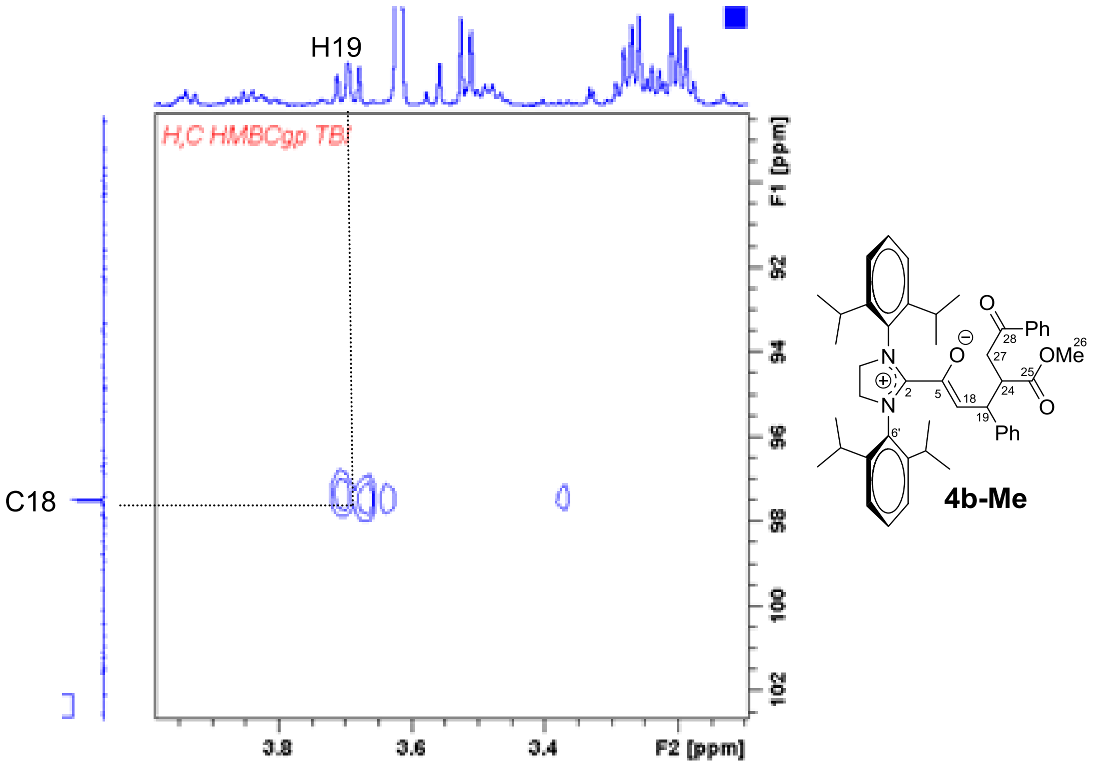

Figure S78. Part of the ^{1}H(600 MHz),^{13}C(150 MHz) HMBC NMR spectrum of **4b-Me** ([D8]THF, 298 K).

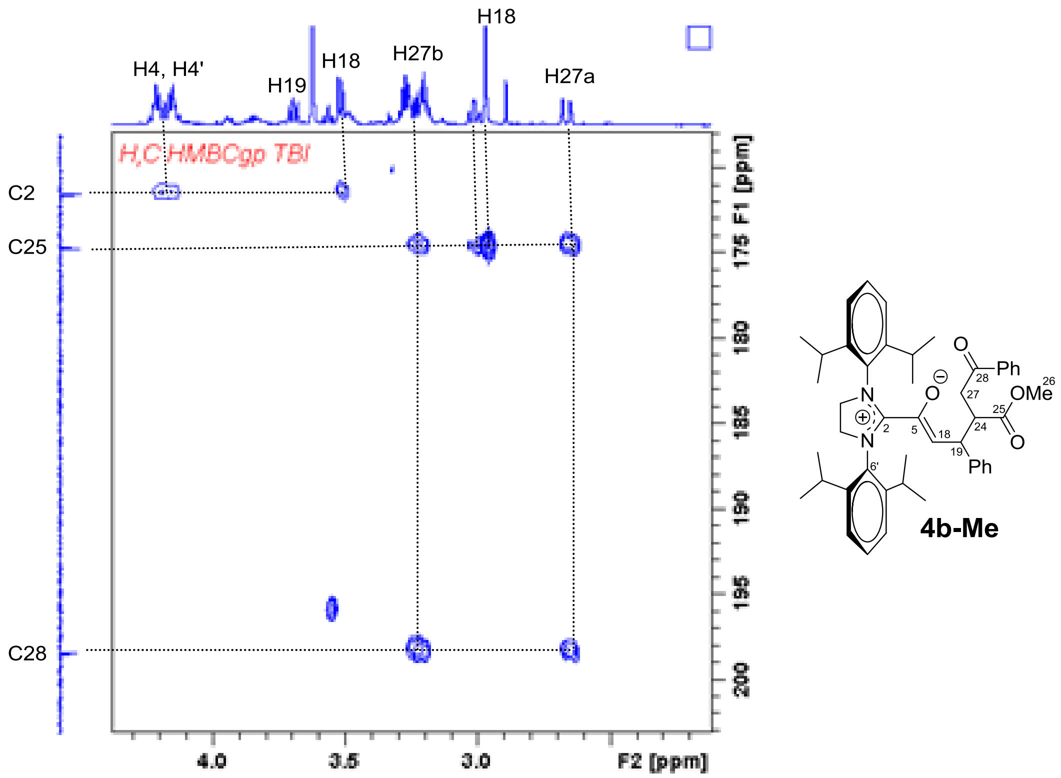

Figure S79. Part of the ^{1}H(600 MHz),^{13}C(150 MHz) HMBC NMR spectrum of **4b-Me** ([D$_8$]THF, 298 K).

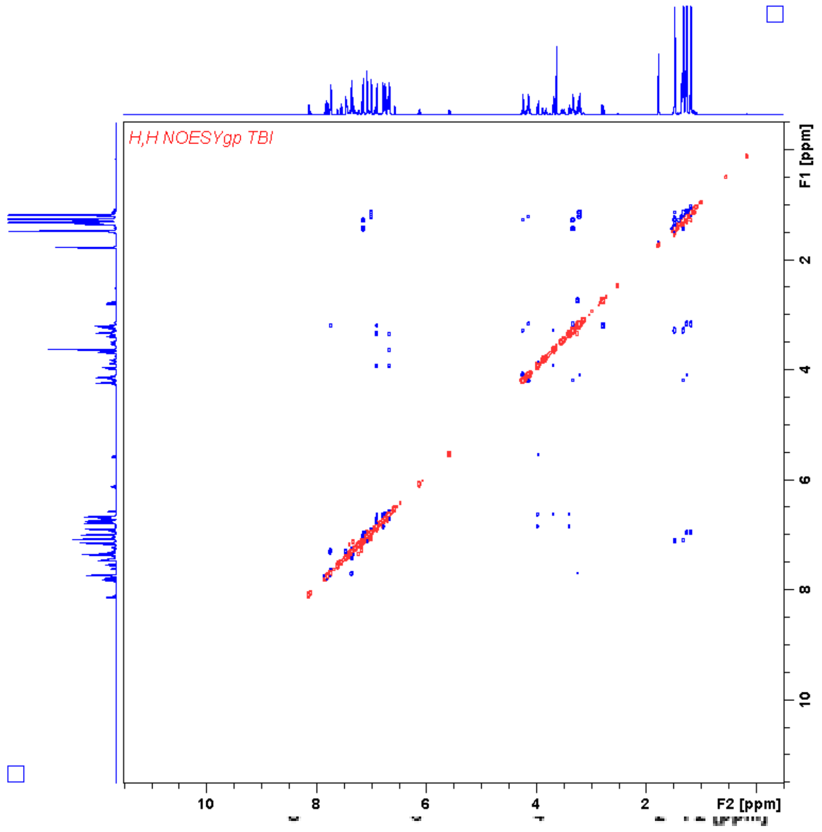

Figure S80. ^{1}H,^{1}H NOESY NMR spectrum of **4b-Me** ([D$_8$]THF, 600 MHz, 298 K, mixing time = 600 ms).

12.11 ^{1}H and ^{13}C NMR spectra of *rac*-ethyl (1*R*,2*R*)-2,4-diphenylcyclopent-3-en-1-carboxylate (5b-Et)

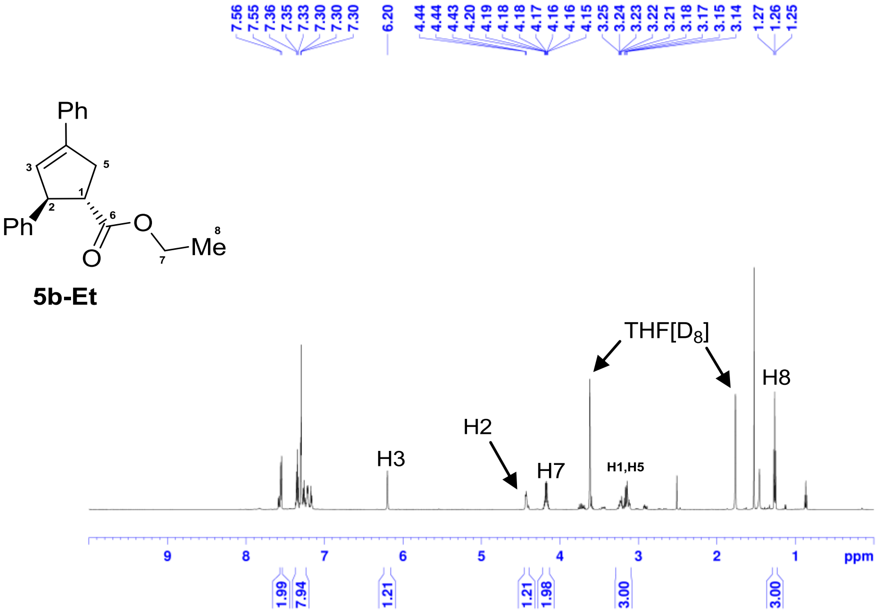

Figure S81. ^{1}H(600 MHz) NMR spectrum of **5b-Et** (trans:cis 4.3:1) ([D$_8$]THF, 298 K).

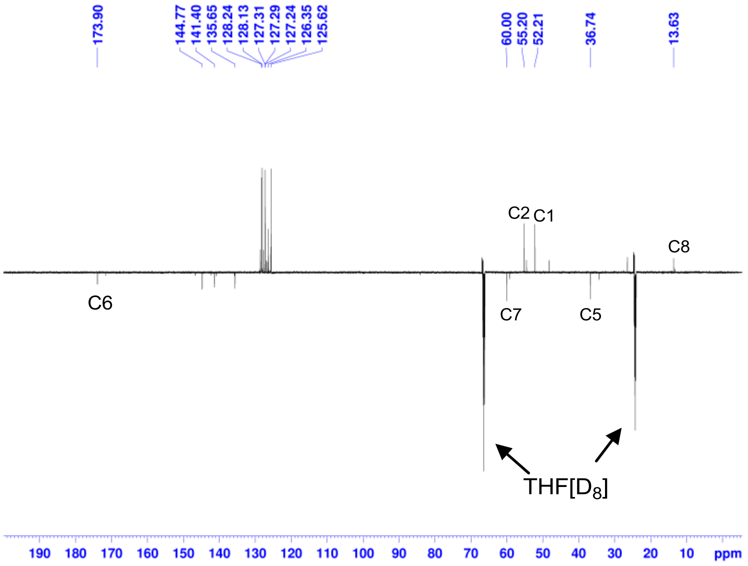

Figure S82. ^{13}C(150 MHz) DEPTQ NMR spectrum of **5b-Et** (trans:cis 4.3:1) ([D$_8$]THF, 298 K).

12.12 ^{1}H and ^{13}C NMR spectra of *rac*-1,1',1''-[(*1R*,*2R*)-cyclopent-3-ene-1,2,4-triyl]tribenzene (5c)

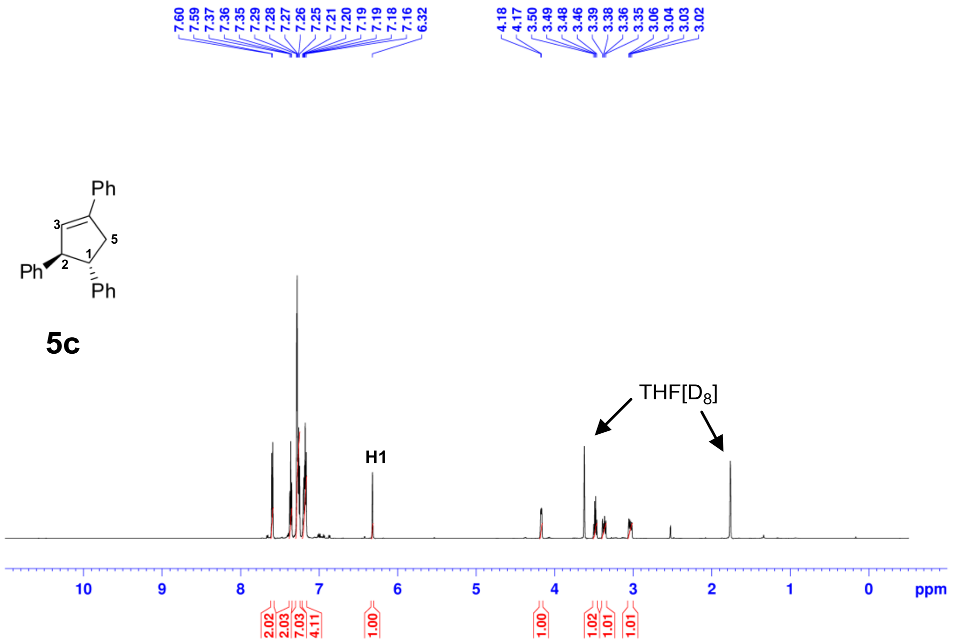

Figure S83. ^{1}H(600 MHz) NMR spectrum of **5c** ([D$_8$]THF, 298 K).

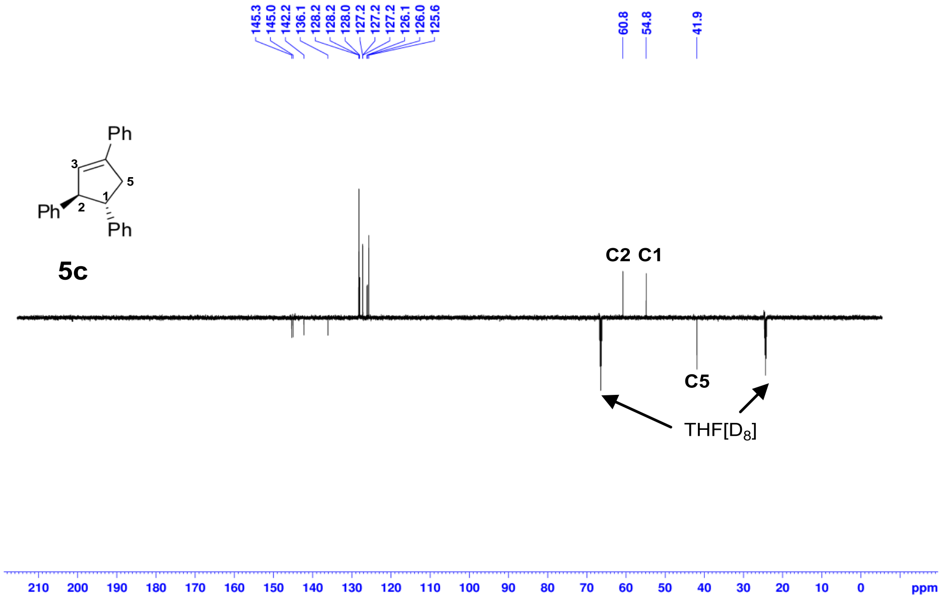

Figure S84. ^{13}C(150 MHz) DEPTQ NMR spectrum of **5c** ([D$_8$]THF, 298 K).

12.13 ^{1}H and ^{13}C NMR spectra of *rac*-(4*R*,5*S*)-4,5-diphenyloxolan-2-one (7)

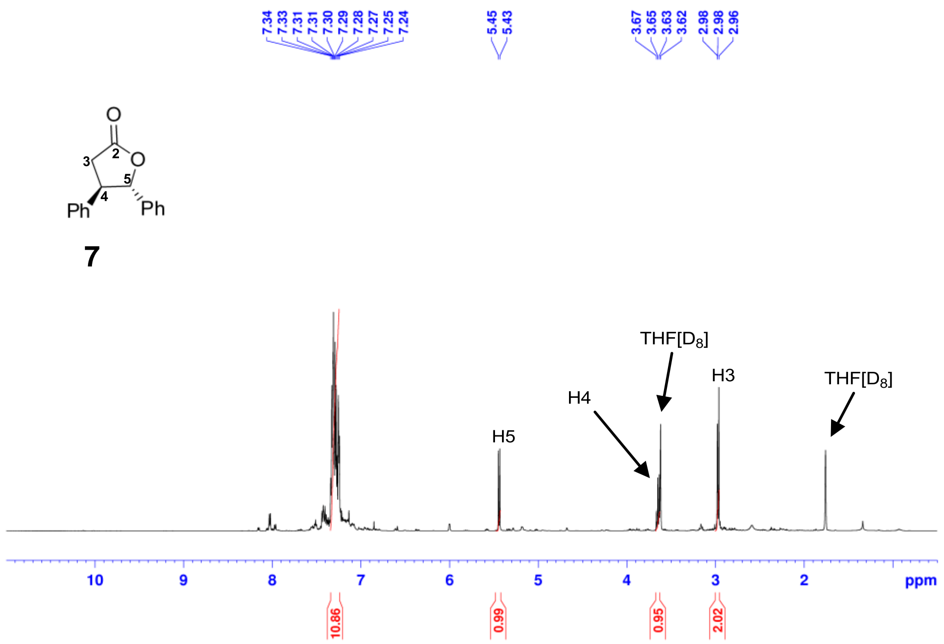

Figure S85. ^{1}H(600 MHz) NMR spectrum of **7** ([D$_8$]THF, 298 K).

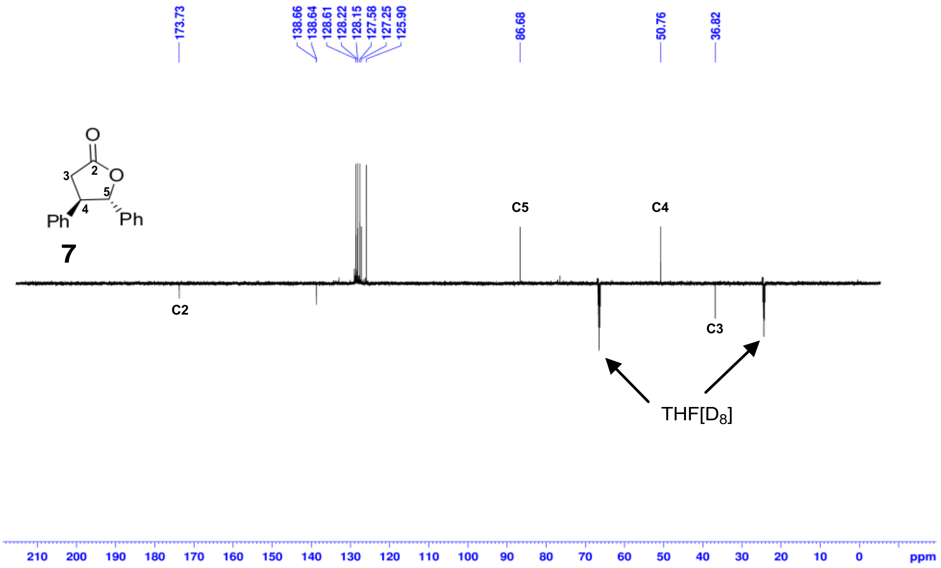

Figure S86. ^{13}C(150 MHz) DEPTQ NMR spectrum of **7** ([D$_8$]THF, 298 K).

12.14 1D and 2D NMR spectra of *rac*-(*3R,4S*)-3-butyl-4,6-diphenyl-3,4-dihydro-2*H*-pyran-2-one (12a)

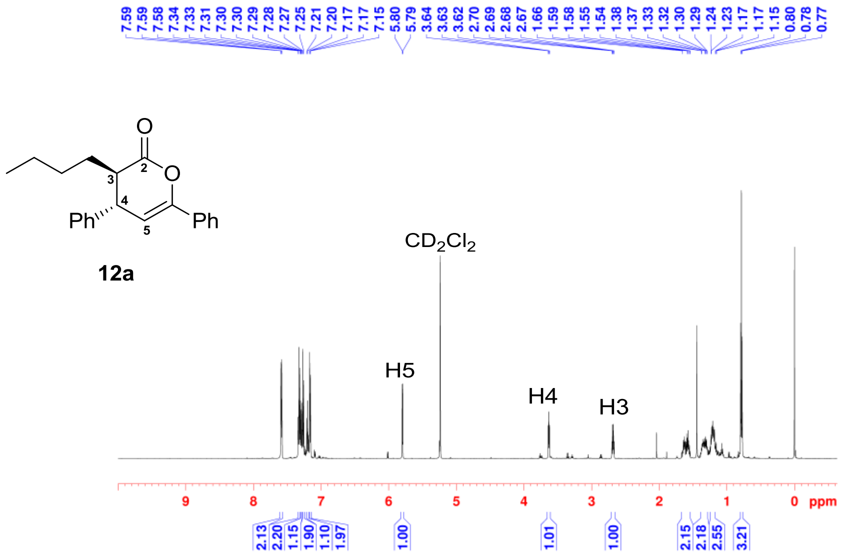

Figure S87. ^{1}H(600 MHz) NMR spectrum of **12a** (CD_2Cl_2, 298 K).

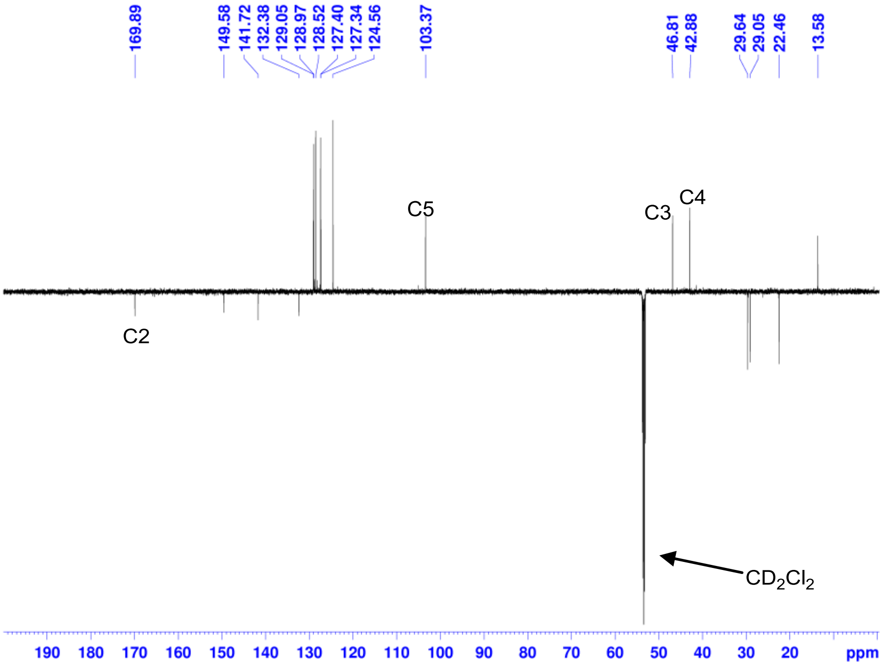

Figure S88. ^{13}C(150 MHz) DEPTQ NMR spectrum of **12a** (CD_2Cl_2, 298 K).

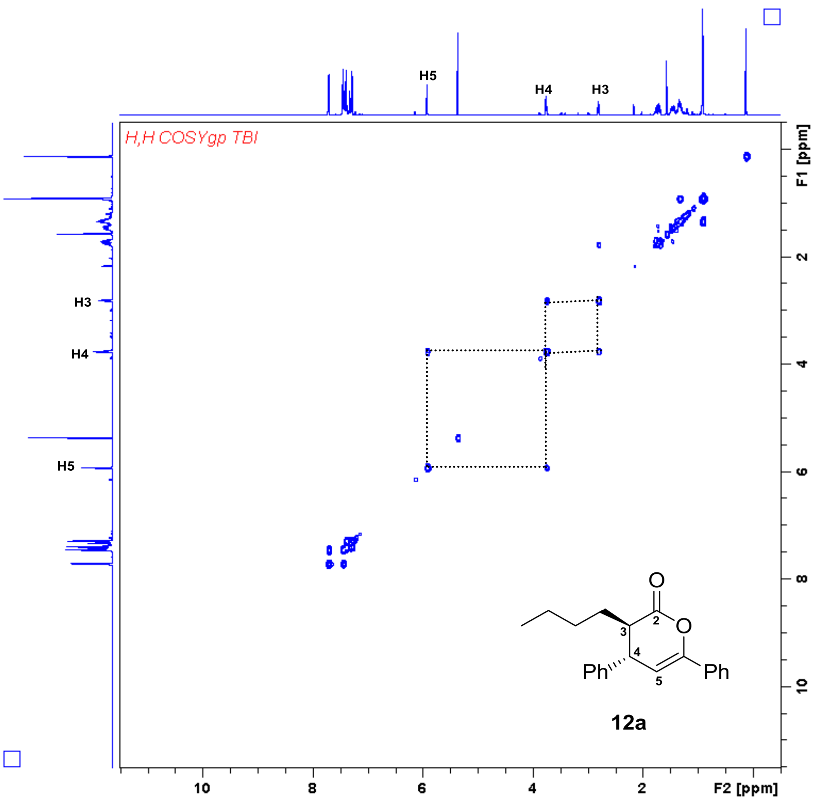

Figure S89. $^{1}H,^{1}H$ (600 MHz) COSY NMR spectrum of **12a** (CD_2Cl_2, 298 K).

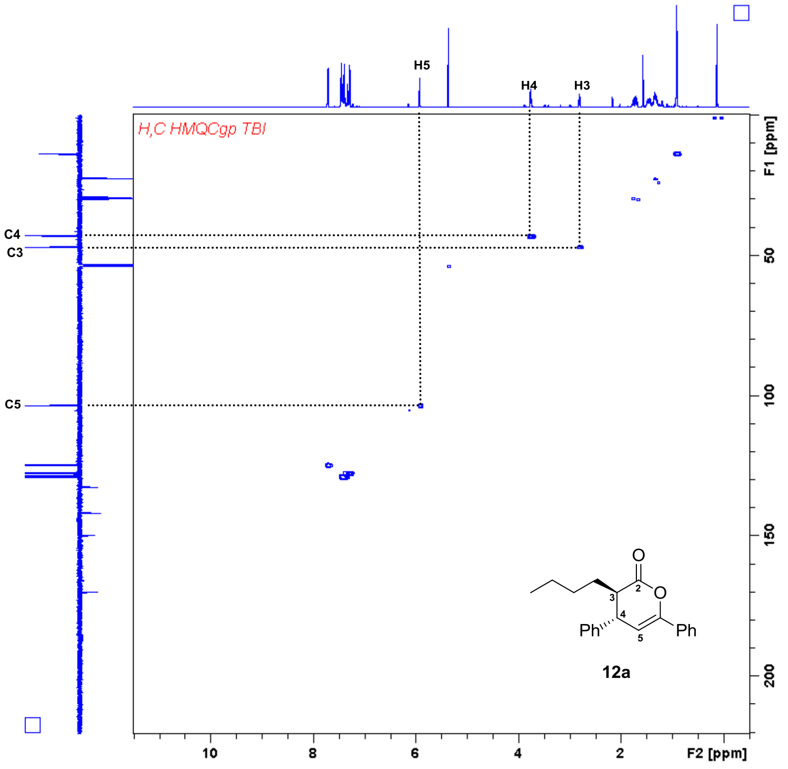

Figure S90. ^{1}H(600 MHz), ^{13}C(150 MHz) HMQC NMR spectrum of **12a** (CD_2Cl_2, 298 K).

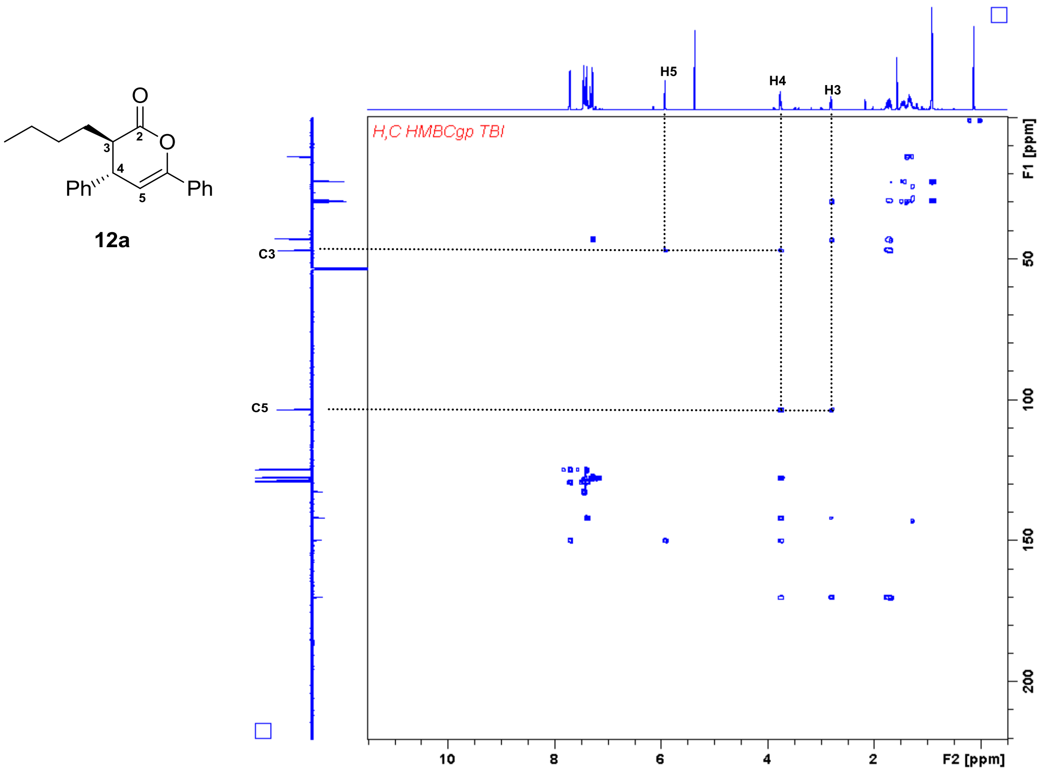

Figure S91. ^{1}H(600 MHz), ^{13}C(150 MHz) HMBC NMR spectrum of **12a** (CD_2Cl_2, 298 K).

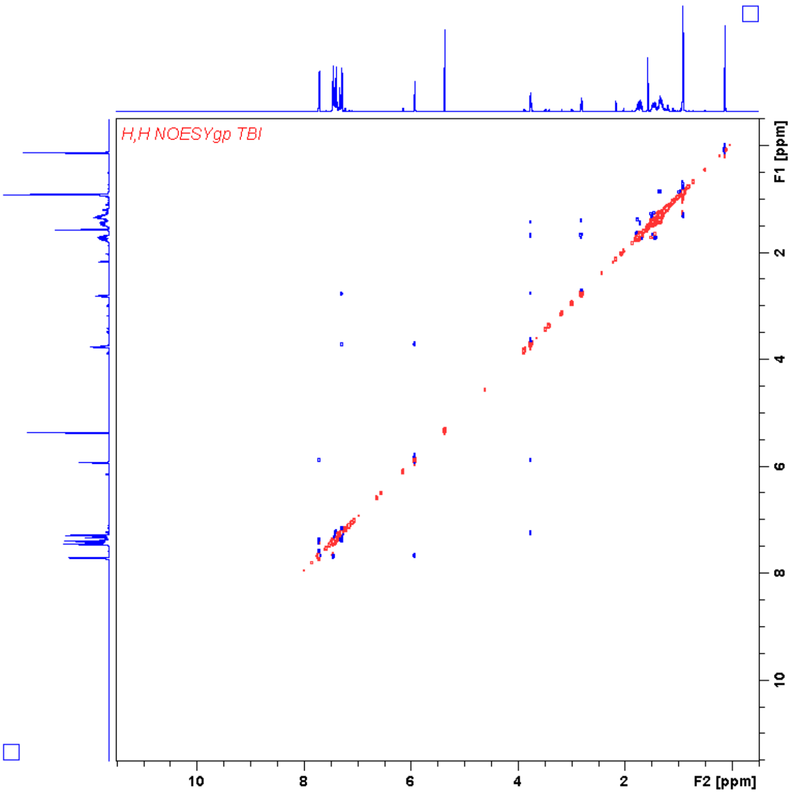

Figure S92. ^{1}H,^{1}H NOESY NMR spectrum of **12a** (CD_2Cl_2, 600 MHz, 298 K, mixing time = 600 ms).

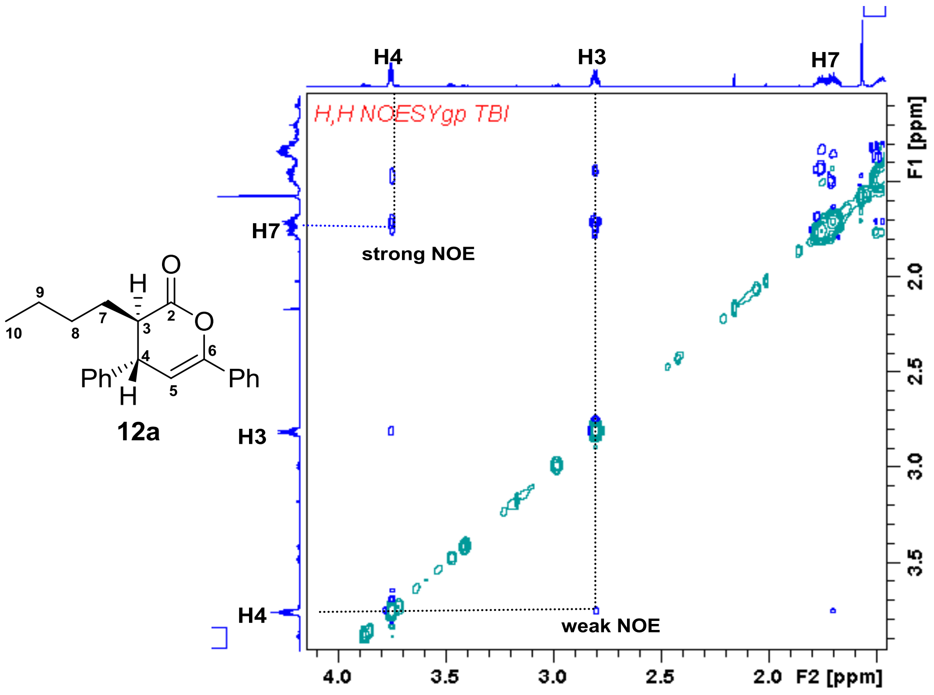

Figure S93. Part of the ^{1}H,^{1}H NOESY NMR spectrum of **12a** (CD_2Cl_2, 600 MHz, 298 K, mixing time = 600 ms).

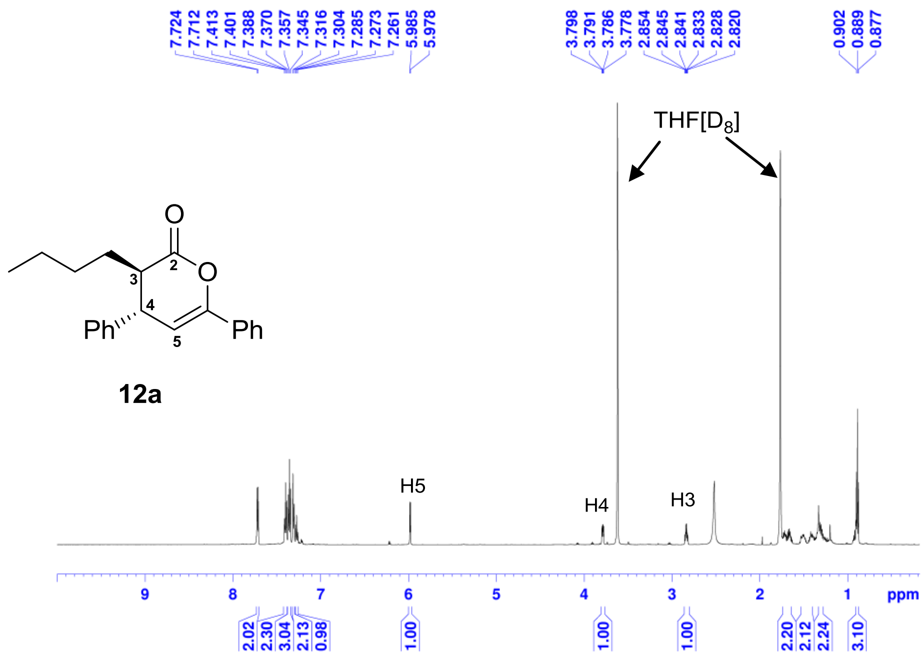

Figure S94. ^{1}H(600 MHz) NMR spectrum of **12a** ([D$_8$]THF, 298 K).

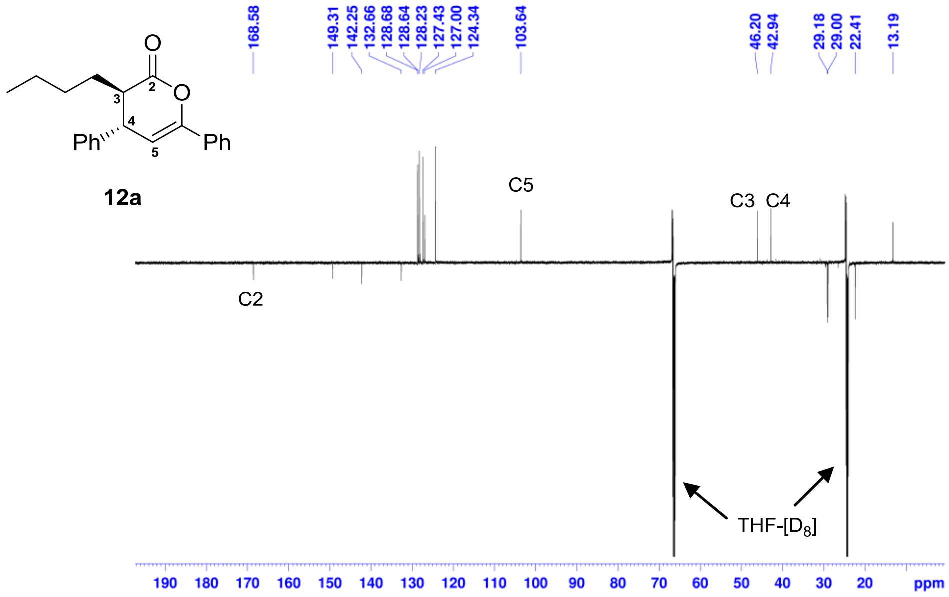

Figure S95. ^{13}C(150 MHz) DEPTQ NMR spectrum of **12a** ([D$_8$]THF, 298 K).

12.15 ^{1}H and ^{13}C NMR spectra of *rac*-(3*R*,4*S*)-4,6-diphenyl-3-(3-phenylpropyl)-3,4-dihydro-2*H*-pyran-2-one (12b)

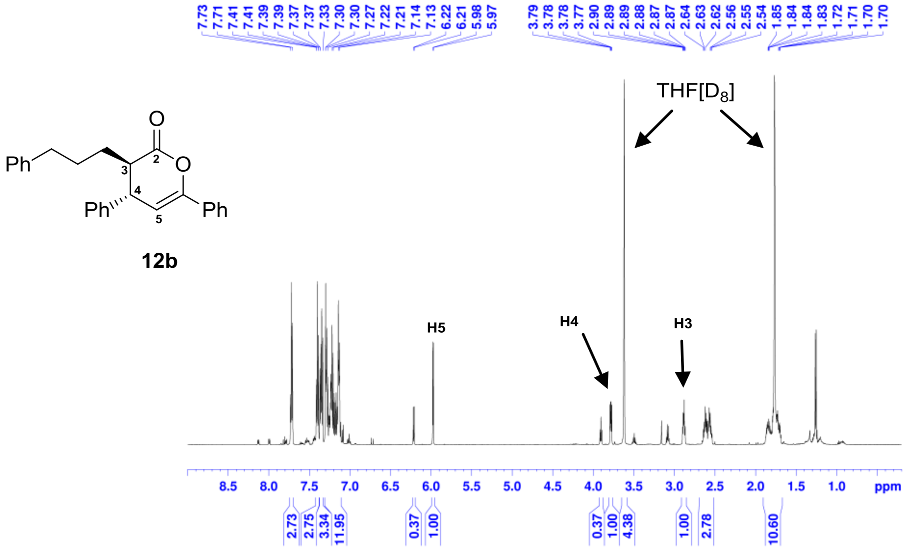

Figure S96. ^{1}H(600 MHz) NMR spectrum of **12b** (trans:cis 2.8:1) ([D$_8$]THF, 298 K).

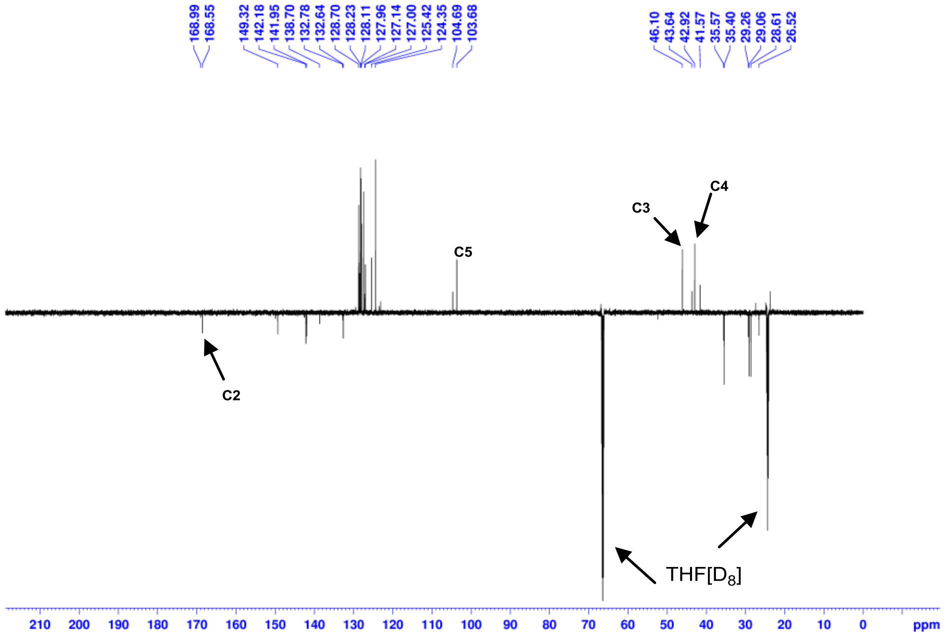

Figure S97. ^{13}C(150 MHz) DEPTQ NMR spectrum of **12b** (trans:cis 2.8:1) ([D$_8$]THF, 298 K).

13 X-ray data of compounds 4b-Et and 4b-Me: Crystal data and structure refinement, selected geometric data, ORTEPs (Oak Ridge Thermal Ellipsoid Plot).

13.1 X-ray data of 4b-Et

Table 1. Crystal data and structure refinement.

Identification code	yvr2079	
Empirical formula	C48 H58 N2 O4, 2(C6 H6)	
Formula weight	883.18	
Temperature	100(2) K	
Wavelength	0.71073 Å	
Crystal system	Triclinic	
Space group	P-1	
Unit cell dimensions	a = 10.8676(15) Å	α = 75.286(7)°.
	b = 12.3446(16) Å	β = 86.422(6)°.
	c = 19.476(3) Å	γ = 86.274(8)°.
Volume	2518.9(6) $Å^3$	
Z	2	
Density (calculated)	1.164 Mg/m^3	
Absorption coefficient	0.072 mm^{-1}	
F(000)	952	
Crystal size	.2 x .1 x .04 mm^3	
Theta range for data collection	1.08 to 25.00°.	
Index ranges	-12<=h<=10, -13<=k<=14, -21<=l<=23	
Reflections collected	9490	
Independent reflections	8128 [R(int) = 0.0270]	
Completeness to theta = 25.00°	91.8 %	
Absorption correction	None	
Refinement method	Full-matrix least-squares on F^2	
Data / restraints / parameters	8128 / 0 / 604	
Goodness-of-fit on F^2	0.913	
Final R indices [I>2sigma(I)]	R1 = 0.0546, wR2 = 0.1071	
R indices (all data)	R1 = 0.1476, wR2 = 0.1456	
Largest diff. peak and hole	0.186 and -0.214 e.$Å^{-3}$	

Table 2. Selected geometric data for **4b-Et**.

**Bond**	**Bond length [Å]**
O4-C13	1.280(4)
N1-C14	1.320(4)
N1-C27	1.474(4)
N1-C15	1.449(4)
N2-C14	1.334(4)
N2-C28	1.481(4)
N2-C29	1.446(4)
C27-C28	1.533(4)
C13-C14	1.505(5)
C13-C12	1.366(4)
C12-C11	1.510(4)
**Angle**	**Angle [°]**
N1-C14-N2	111.3(3)
**Torsion angles**	**Torsion angles [°]**
N2-C14-C13-O4	-132.3(3)
N1-C14-C13-C12	-132.6(3)

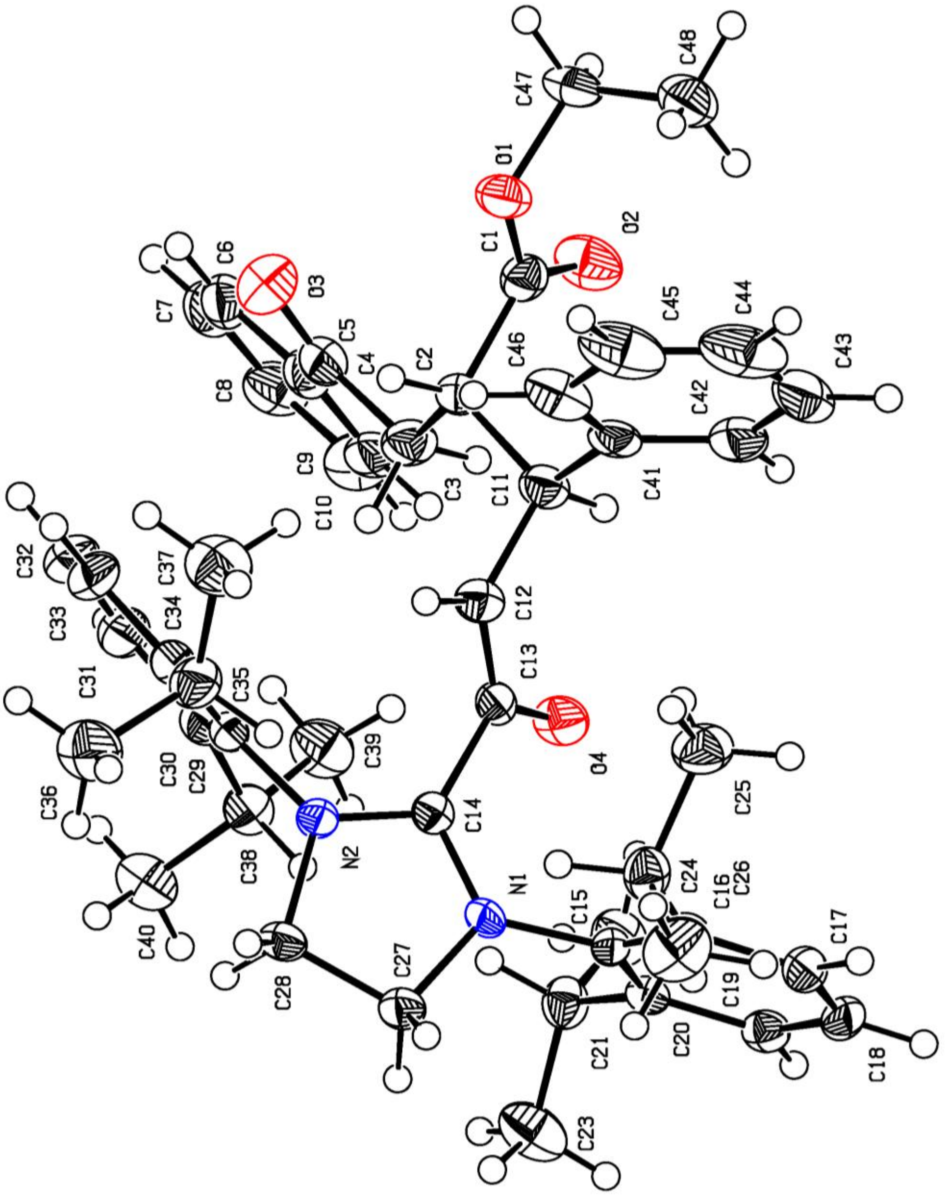

Figure S98. ORTEP of the X-ray crystal structure of **4b-Et**. Thermal ellipsoids are drawn at 50% probability level (solvent molecules are omitted for clarity).

13.2 X-ray data of 4b-Me

Table 3. Crystal data and structure refinement.

Identification code	yvr1072	
Empirical formula	C47 H56 N2 O4,2(C4 H8 O1)	
Formula weight	857.15	
Temperature	100(2) K	
Wavelength	0.71073 Å	
Crystal system	Monoclinic	
Space group	P21/c	
Unit cell dimensions	a = 12.3090(19) Å	α = 90°.
	b = 37.444(7) Å	β = 91.780(8)°.
	c = 10.6821(17) Å	γ = 90°.
Volume	4921.0(14) $Å^3$	
Z	4	
Density (calculated)	1.157 Mg/m^3	
Absorption coefficient	0.074 mm^{-1}	
F(000)	1856	
Crystal size	.3 x .3 x .15 mm^3	
Theta range for data collection	1.66 to 25.00°.	
Index ranges	-14<=h<=9, -44<=k<=24, -12<=l<=4	
Reflections collected	8764	
Independent reflections	6351 [R(int) = 0.0642]	
Completeness to theta = 25.00°	73.2 %	
Absorption correction	None	
Refinement method	Full-matrix least-squares on F^2	
Data / restraints / parameters	6351 / 0 / 578	
Goodness-of-fit on F^2	0.917	
Final R indices [I>2sigma(I)]	R1 = 0.0678, wR2 = 0.1658	
R indices (all data)	R1 = 0.1409, wR2 = 0.1922	
Extinction coefficient	0.0077(10)	
Largest diff. peak and hole	0.494 and -0.264 e.$Å^{-3}$	

Table 4. Selected geometric data for **4b-Me**.

**Bond**	**Bond length [Å]**
O4-C14	1.271(5)
N1-C15	1.345(5)
N1-C28	1.478(5)
N1-C16	1.434(5)
N2-C15	1.316(5)
N2-C29	1.482(5)
N2-C30	1.442(5)
C14-C15	1.510(6)
C14-C13	1.353(6)
C13-C12	1.503(6)
**Angle**	**Angle [°]**
N1-C15-N2	110.6(4)
**Torsion angles**	**Torsion angles [°]**
N2-C15-C14-O4	47.8(5)
N1-C15-C14-C13	52.7(5)

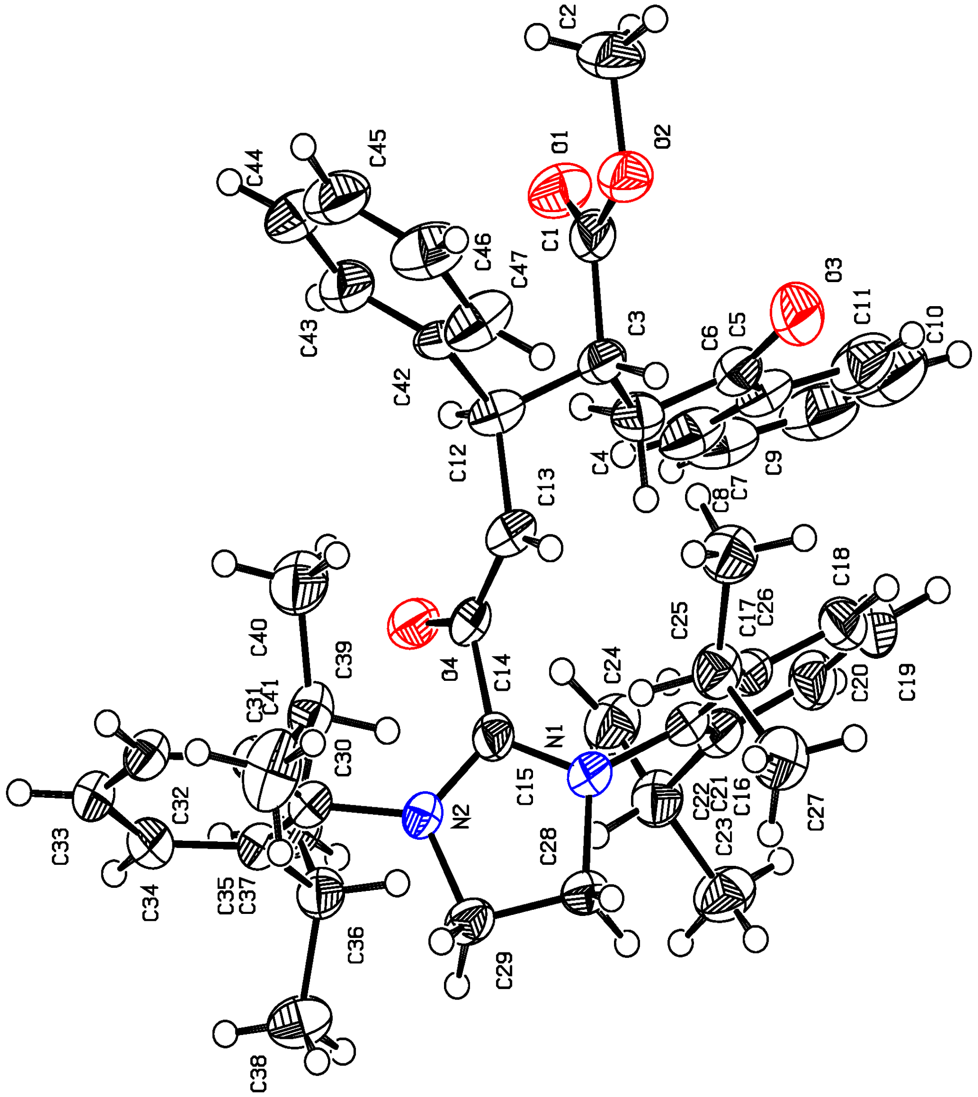

Figure S99. ORTEP of the X-ray crystal structure of **4b-Me**. Thermal ellipsoids are drawn at 50% probability level (solvent molecules are omitted for clarity).

14 References

1. A. Schmidt, G. Hilt, *Org. Lett.* **2013**, *15*, 2708-2711.
2. B. J. Drakulić et al., *Eur. J. Med. Chem*, 2011, 46, 3265-3273.
3. P.-C. Chiang, M, Rommel, J.W. Bode, *J. Am. Chem. Soc.* **2009**, *131*, 8714-8718.
4. V. Nair, S. Vellalath, M. Poonoth, E. Suresh, *J. Am. Chem. Soc.* **2006**, *128*, 8736-8737.
5. C. Burstein, F. Glorius, *Angew. Chem. Int. Ed.* **2004**, *43*, 6205-6208.
6. D. J. Pippel, M. D. Curtis, H. Du, P. Beak, *J. Org. Chem.***1998**, *63*, 2-3.
